# Precision-activated T-cell engagers targeting HER2 or EGFR and CD3 mitigate on-target, off-tumor toxicity for immunotherapy in solid tumors

**DOI:** 10.1038/s43018-023-00536-9

**Published:** 2023-03-30

**Authors:** Fiore Cattaruzza, Ayesha Nazeer, Milton To, Mikhail Hammond, Caitlin Koski, Lucas Y. Liu, V. Pete Yeung, Deena A. Rennerfeldt, Angela Henkensiefken, Michael Fox, Sharon Lam, Kari M. Morrissey, Zachary Lange, Vladimir N. Podust, Mika K. Derynck, Bryan A. Irving, Volker Schellenberger

**Affiliations:** Amunix Pharmaceuticals, a Sanofi Company, South San Francisco, CA USA

**Keywords:** Drug discovery, Cancer, Cancer, Cancer immunotherapy

## Abstract

To enhance the therapeutic index of T-cell engagers (TCEs), we engineered masked, precision-activated TCEs (XPAT proteins), targeting a tumor antigen (human epidermal growth factor receptor 2 (HER2) or epidermal growth factor receptor (EGFR)) and CD3. Unstructured XTEN polypeptide masks flank the N and C termini of the TCE and are designed to be released by proteases in the tumor microenvironment. In vitro, unmasked HER2-XPAT (uTCE) demonstrates potent cytotoxicity, with XTEN polypeptide masking providing up to 4-log-fold protection. In vivo, HER2-XPAT protein induces protease-dependent antitumor activity and is proteolytically stable in healthy tissues. In non-human primates, HER2-XPAT protein demonstrates a strong safety margin (>400-fold increase in tolerated maximum concentration versus uTCE). HER2-XPAT protein cleavage is low and similar in plasma samples from healthy and diseased humans and non-human primates, supporting translatability of stability to patients. EGFR-XPAT protein confirmed the utility of XPAT technology for tumor targets more widely expressed in healthy tissues.

## Abstract (web summary)

Schellenberger and colleagues engineer masked, conditionally active T-cell engagers targeting tumor antigens and CD3 and show potent, protease-dependent antitumor activity in mouse models, as well as safety in non-human primates.

## Main

Therapies exploiting the endogenous immune system to enhance tumor regression are providing important advances in oncology^1^. Immune-checkpoint inhibitors promote T-cell-mediated responses across many solid tumor types but require tumor-specific T-cell immunity; thus, large patient subsets and tumor types do not respond^2,3^. TCEs are highly potent modalities directing T-cell cytotoxicity toward tumors expressing a selected tumor-associated antigen (TAA) while bypassing T-cell recognition requirements of major histocompatibility complex (MHC)-bound tumor antigen peptides^1^. TCE potency arises from cytotoxicity induction and cytokine-driven actions downstream of T-cell activation that enhance and amplify the antitumor immune response^4^. TCEs demonstrate impressive clinical activity in hematologic cancers and ‘cold’ solid tumors unresponsive to other immune therapies^1,5,6^.

**Fig. 1:**
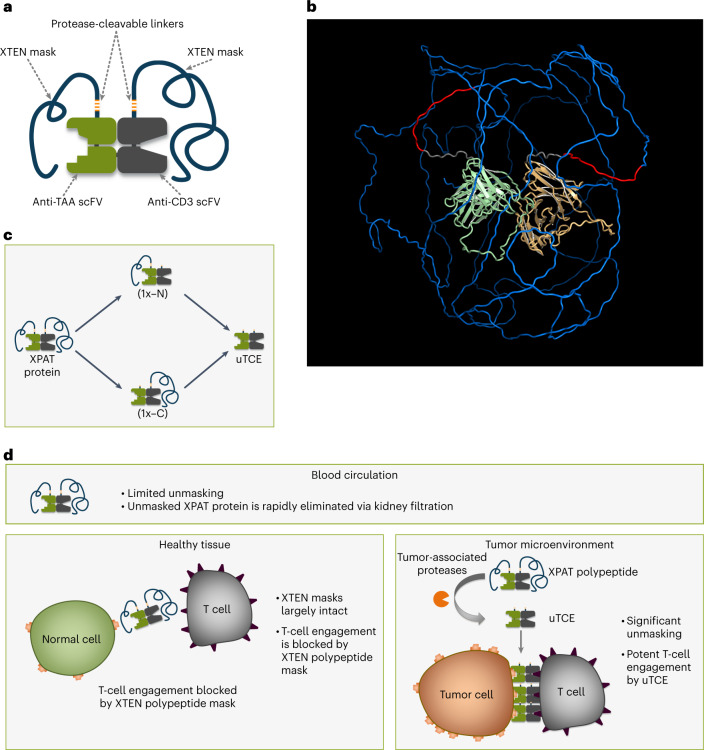
Structure and mechanism of action of XPAT therapeutics. **a**, An XPAT protein comprises a TCE core with two scFvs, one targeting CD3 and the other, a TAA. Each scFv is masked by a protease-releasable XTEN mask, unstructured, hydrophilic polypeptides that act as modular, tunable masks, in addition to extending the half-life of the TCE. Each XTEN mask connects to the TCE core via a protease-cleavable linker, designed to be cleavable by any of eight proteases from three different classes (matrix metalloproteinases, serine proteases and cysteine proteases) involved in cancer progression^41^. **b**, Predicted structure of HER2-XPAT protein visualized using AlphaFold2 v.2.0, a machine-learning-based computational method for predicting protein structures with reasonable accuracy^42^. Colors indicate anti-HER2 domain, pale green; anti-CD3 domain, light orange; XTEN masks, blue; protease-cleavable linker, red; linkers, gray. The model represents a static picture showing a plausible conformation of the unstructured XTEN masks and the length of unstructured XTEN relative to the folded antibody domains in an XPAT protein. **c**, XPAT proteins are expected to remain largely intact in healthy tissues, where protease activity is well controlled by protease inhibitors. XPAT protein unmasking occurs in two steps via one of two potential paths to the fully unmasked state. The two requisite cleavage events can occur in either order and each sequence (either the top or bottom paths shown) is equally likely. In aggregate, both 1x-N and 1x-C partially unmasked forms will exist, depending on the cleavage path. Removal of both XTEN masks liberates the unmasked HER2-TCE (uTCE). **d**, XPAT proteins are designed to exploit the dysregulated protease activity present in tumors versus healthy tissues and expand the therapeutic index of TCEs through preferential unmasking in the TME. The active uTCE promotes the formation of immunologic synapses between tumor and T cells, resulting in potent cytotoxicity. Notably, the uTCE has a short half-life and should be rapidly cleared, thereby sparing healthy tissues when the uTCE diffuses away from the TME. By design, the molecular weight of the uTCE (∼59 kDa) is sufficiently small to allow rapid kidney filtration^43^.

The clinical utility of current bispecific TCEs for the treatment of solid tumors is hampered by on-target, off-tumor toxicity, which limits the achievement of a favorable therapeutic index^1,7–9^. To expand the therapeutic index of bispecific TCEs and mitigate off-tumor toxicity, we developed a protease-releasable masking technology (Pro-XTEN) to create precision-activated TCEs attached to XTEN proteins (XPAT proteins). An XPAT protein comprises a TCE core with two single-chain variable fragments (scFv) targeting a TAA and CD3. Each scFv is linked to an XTEN mask via a protease-cleavable linker, designed to be cleaved by three protease classes (matrix metalloproteinases, serine proteases and cysteine proteases). XTEN masks are unstructured, hydrophilic polypeptides that act as modular, tunable masks and extend the TCE half-life. The structure and proposed mechanism of action of XPAT proteins are shown in Fig. 1 (Supplementary Tables 1 and 2 provide further details). XTEN masks were used previously as an alternative to PEGylation to extend the half-life of human growth hormones and clotting factor VIII (FVIII). Clinical testing of these applications has demonstrated favorable safety and low immunogenicity potential of XTEN sequences^10–14^.

We report the design and nonclinical characterization of XPAT proteins targeting well-validated TAAs: HER2 and EGFR. HER2-XPAT protein demonstrates potent in vivo antitumor activity similar to that of its uTCE counterpart, while achieving >400-fold higher tolerated maximum concentration (C_max_) in non-human primates (NHPs). Preferential cleavage of the XPAT protein to the uTCE form occurs within tumor xenografts in mice with minimal cleavage in healthy tissues. EGFR-XPAT protein achieved in vivo antitumor activity and >200-fold higher tolerated C_max_ in NHPs versus its uTCE. Key to the safety of XPAT proteins, HER2-XPAT protein stability with minimal cleavage in vitro in plasma from healthy and diseased humans and NHPs and in vivo in NHPs is demonstrated. These results provide nonclinical proof of concept for the XPAT protein mechanism of action and support phase I clinical evaluation of HER2-XPAT protein in solid tumors.

## Results

### XPAT protein design and production

XPAT proteins are produced as single contiguous polypeptides in *Escherichia* *coli* in a secreted and fully folded form. *E.* *coli* production precludes glycosylation that commonly occurs in Fc-containing TCEs produced in mammalian expression systems. Therefore, XPAT proteins can be purified as highly homogeneous proteins observed as single-peak species by intact mass spectrometry analysis. The XPAT protein design relies on the small size of scFvs (and other antibody fragments) to form the two binding sites of the TCE. scFvs are prone to form multimers due to intermolecular pairing of the multiple immunoglobulin domains; however, the large size and hydrophilic nature of XTEN polypeptides ssubstantially reduces the tendency of XPAT proteins to form such multimers and other aggregates. The net negative charge of XTEN polypeptides enables efficient purification of fully masked monomeric XPAT molecules. The XPAT protein prototypes evaluated in these studies targeted TAAs that are well-established clinical targets in oncology (HER2, EGFR and epithelial cell adhesion molecule (EpCAM); structural details of the XPAT proteins evaluated are provided in Supplementary Tables 1 and 2). Further details about the design and production of XPAT proteins are provided in the Methods.

### HER2-XPAT binding affinities and cytotoxicity

**Fig. 2:**
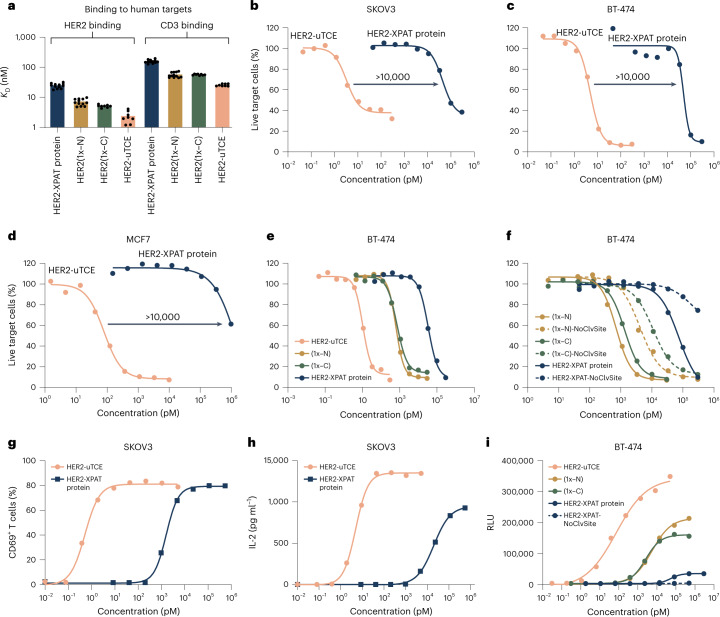
Binding and in vitro activity of HER2-XPAT protein, its partially unmasked metabolites and unmasked HER2-TCE. **a**, Binding affinities to human HER2 and CD3 at 37 °C by surface plasmon resonance. Data are *K*_D_ (*n* = 12 technical replicates of a single experiment for HER2-XPAT protein and HER2(1x-N); *n* = 8 for HER2(1x-C) and uTCE). Surface plasmon resonance sensorgrams for these data are provided in Supplementary Figs. 3–10. **b**–**d**, In vitro tumor cytotoxicity of HER2-XPAT protein and its metabolites following a 48-h incubation with co-cultures of huPBMCs and the high HER2-expressing human tumor cell lines (1:1 effector–target ratio) SKOV3 (**b**), BT-474 (**c**) or the medium-low HER2-expressing MCF7 cell line (**d**). **e**, In vitro cytotoxicity of HER2-XPAT protein and its metabolites against BT-474 cells co-cultured with huPBMCs (1:1 effector–target ratio). **f**, Impact of the protease-cleavable linker on in vitro cytotoxicity versus BT-474 cells co-cultured with huPBMCs. **g**,**h**, CD69-positive T cells (**g**) and IL-2 secretion (**h**) following 72-h incubation of huPBMC/SKOV3 co-cultures with HER2-XPAT protein or its unmasked form (uTCE). **i**, Target-dependent T-cell activation with HER2-XPAT protein and its metabolites. CD3-expressing Jurkat reporter T cells were incubated with BT-474 cells at a 5:1 effector–target ratio for 6 h, followed by quantification of *NFAT*-induced luciferase activity and measured in relative luminescence units (RLUs). Mean data for *n* = 2 technical replicates within one single experiment (**b**–**i**). Extended Data Table 1 provides a summary of EC_50_ values for the different forms of the HER2-XPAT proteins in the cytotoxicity and reporter T-cell activation assays. Source data

**Extended Data Fig. 1:**
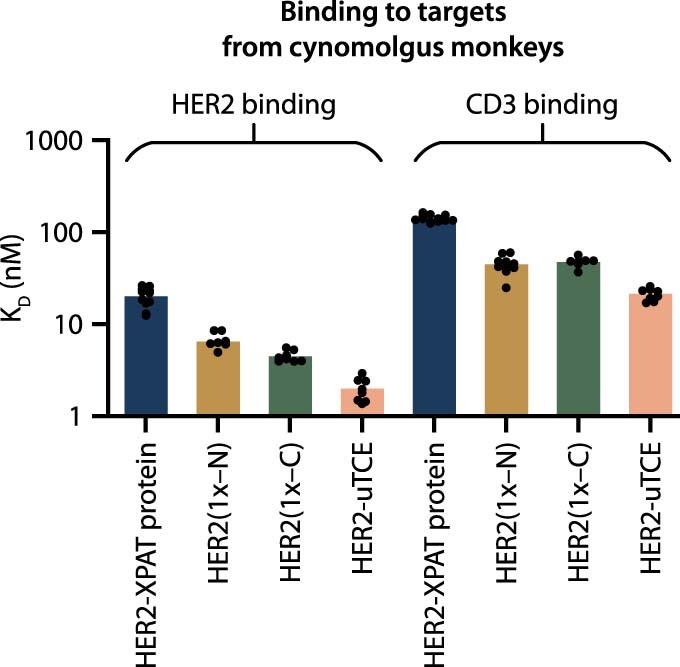
Binding affinity of HER2-XPAT protein and its partially and fully unmasked metabolites to cynomolgus monkey HER2 and CD3. Binding was assessed *in vitro* at 37°C by surface plasmon resonance. Data are K_D_ (*n*=8 to 12 technical replicates of a single experiment; individual data points are displayed on the graph). Surface plasmon resonance sensorgrams for these data are provided in Supplementary Figs. 11–18. HER2, human epidermal growth factor receptor 2; uTCE, unmasked HER2 T-cell engager; XPAT proteins, TCEs fused to XTEN polypeptides. Source data

The effect of XTEN polypeptide masking on binding and in vitro activity was investigated via HER2-targeted XPAT protein. Kinetic analysis by surface plasmon resonance evaluated the affinity of HER2-XPAT protein and its active metabolites for human HER2 and CD3ε (Fig. 2a). The presence of XTEN masks reduced target affinities tenfold for HER2 and approximately sixfold for CD3 compared to unmasked HER2-TCE. The affinity (*K*_D_) of HER2-XPAT protein for human HER2 was 24.9 ± 4.3 nM and for human CD3 was 160.0 ± 19.5 nM. The affinity of the unmasked HER2-TCE for HER2 was 2.5 ± 0.9 nM and for CD3 was 26.3 ± 2.2 nM. The affinities of HER2-XPAT protein and its metabolites were similar for cynomolgus monkey HER2 and CD3 (Extended Data Fig. 1).

In vitro cytotoxicity of HER2-XPAT protein and its metabolites was investigated using co-cultures of human peripheral blood mononuclear cells (huPBMCs) with HER2-expressing human tumor cell lines. The proteolytically activated, unmasked HER2-TCE demonstrated highly potent cytotoxicity against high HER2-expressing tumor cells, SKOV3 (∼650,000 HER2 receptors per cell; Fig. 2b) and BT-474 (∼975,000 HER2 receptors per cell; Fig. 2c), showing nearly complete target cell killing with 50% effective concentrations (EC_50_) in the single-digit picomolar range. Cytotoxicity was strongly attenuated with fully masked HER2-XPAT protein (average EC_50_ shifted by ~4 logs), demonstrating the robust functional protection provided by the two XTEN polypeptide masks. Unmasked HER2-TCE was also cytotoxic in the medium-low HER2-expressing cell line, MCF7 (∼12,500 receptors per cell), although with a 7–16-fold higher EC_50_ than seen with high HER2-expressing cells (Fig. 2d). With MCF7, a similar >4-log attenuation of cytotoxicity was observed with masked HER2-XPAT protein versus unmasked HER2-TCE. A single mask on HER2-XPAT protein (at the C- or N-terminal end of the TCE) was associated with an intermediate cytotoxicity level in the BT-474 cell line (Fig. 2e).

The low cytotoxicity level with HER2-XPAT protein in HER2-expressing tumor cell lines was partly due to a low degree of proteolytic cleavage during the in vitro assay. This is supported by further reduction in cytotoxicity seen with HER2-XPAT protein lacking one or both of its protease cleavage sites (HER2-XPAT-NoClvSite variants) (Fig. 2f).

### Target-dependent T-cell activation with HER2-XPAT protein

T-cell activation by HER2-XPAT protein and its proteolytically activated unmasked HER2-TCE was characterized utilizing primary huPBMCs co-cultured with SKOV3 tumor cells (HER2-high). Incubation with unmasked HER2-TCE for 72 h led to dose-dependent upregulation of the activation marker CD69 on T cells (Fig. 2g) and secretion of the pro-inflammatory cytokine interleukin (IL)-2 (Fig. 2h). CD69 expression and IL-2 secretion were markedly attenuated with HER2-XPAT protein versus unmasked HER2-TCE.

T-cell activation by HER2-XPAT protein and its proteolytically activated metabolites was characterized using Jurkat *NFAT*-luciferase reporter T cells co-cultured with BT-474 tumor cells (Fig. 2i). Unmasked HER2-TCE activated Jurkat *NFAT*-luciferase reporter T cells in the presence of BT-474 cells with EC_50_ values in the sub-nanomolar range (accurate EC_50_ values were not obtained due to incomplete saturation of the response). A 4-log differential in the concentrations required to initiate signaling was observed between HER2-XPAT protein and unmasked HER2-TCE. HER2-XPAT protein at 3 μM achieved maximal activation of only ∼10% of that observed with unmasked HER2-TCE, confirming the ability of XTEN polypeptide masking to reduce T-cell receptor (TCR) activation. The presence of a single mask (at the C- or N-terminal end of the TCE) showed intermediate activation. HER2-XPAT-NoClvSite induced no detectable T-cell activation, indicating the minimal response with HER2-XPAT protein was likely driven by cleavage resulting from active proteases released during the assay.

**Extended Data Fig. 2:**
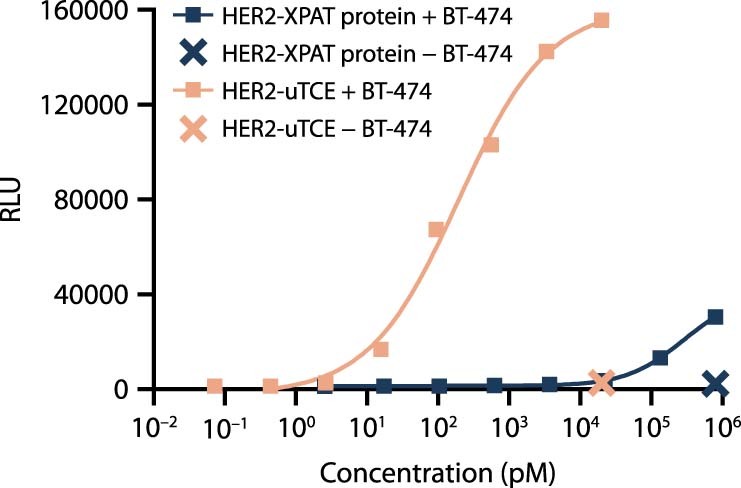
Target-dependent T-cell activation with HER2-XPAT protein and unmasked HER2-TCE. CD3^+^ Jurkat reporter T cells were incubated with or without BT-474 cells at a 5ː1 effector-target ratio for 6 hours, followed by quantification of *NFAT*-induced luciferase activity. The graph shows individual data points from a single representative experiment. There were *n* = 2 biological repeats at each concentration tested within the same experiment for each cell line. HER2, human epidermal growth factor receptor 2; RLU, relative luminescence unit; uTCE, unmasked T-cell engager; XPAT proteins, TCE fused to XTEN polypeptides. Source data

T-cell activation by unmasked HER2-TCE was negligible in the absence of HER2-expressing BT-474 tumor cells, demonstrating that monovalent engagement of CD3 was insufficient for activation (see Extended Data Fig. 2).

### HER2-XPAT protein in HER2-positive human tumor xenograft models

In vivo antitumor activity of HER2-XPAT protein was assessed in the HER2-high BT-474 human breast tumor model (∼975,000 HER2 receptors per cell) and HER2-low HT-55 colorectal cancer model (∼25,000 HER2 receptors per cell) inoculated subcutaneously (s.c.) into immunodeficient mice and engrafted with huPBMCs as a source of T cells.

**Fig. 3:**
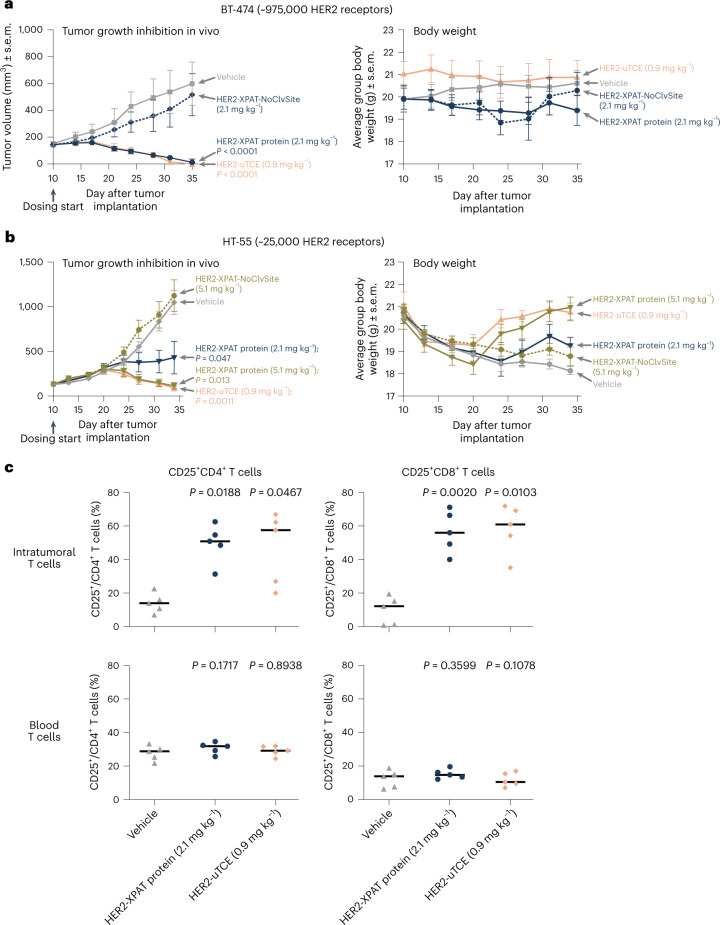
Effects of HER2-XPAT protein and unmasked HER2-TCE in HER2-high BT-474 human breast cancer and HER2-low HT-55 colorectal cancer xenografts, engrafted with huPBMCs. **a**, TGI ± s.e.m. (*n* = 8 mice for each concentration tested within one single experiment) promoted by the i.v. administration of equimolar doses (every week (QW) for 3 weeks) of HER2-XPAT protein (2.1 mg kg^−1^) or uTCE to NOG mice bearing established (maximum tolerated volume (MTV) ~185 mm^3^) BT-474 human tumors. The dependence of tumor-resident proteases for activity was demonstrated by the lack of significant TGI in mice treated with HER2-XPAT-NoClvSite. The average body weight of the mice bearing tumor xenografts remained generally stable for the duration of the experiment following dosing with the HER2-XPAT proteins or the vehicle control. **b**,TGI ± s.e.m. (*n* = 8 mice for each concentration tested within one single experiment) in established human HT-55 xenografts (MTV ∼150 mm^3^) following i.v. administration of HER2-XPAT protein (QW for 4 weeks) and HER2-uTCE (0.9 mg kg^−1^ three times a week (TIW) for 4 weeks). HER2-XPAT-NoClvSite (QW for 4 weeks) had no impact on tumor growth. The average body weight ± s.e.m. of the mice bearing tumor xenografts remained generally stable for the duration of the experiment following dosing with the HER2-XPAT proteins or the vehicle control. **c**, T-cell activation in BT-474 human tumor xenografts and peripheral blood evaluated by flow cytometry on day 18 following equimolar TIW i.v. dosing with HER2-XPAT protein and HER2-uTCE (day 40 following tumor inoculation). HER2-XPAT and HER2-uTCE induced robust and comparable activation of intratumoral CD4^+^ and CD8^+^ T cells, whereas no trends for T-cell activation were apparent in blood samples in which HER2 was not present. Statistical differences in TGI and T-cell activation for test compounds versus vehicle were assessed using mixed-effects multiple comparison analyses followed by Tukey’s post hoc test. Source data

Equimolar HER2-XPAT protein (2.1 mg kg^−1^) or unmasked HER2-TCE-induced robust and complete tumor regression within 35 d of dosing (*P* < 0.001 for both versus vehicle control at day 35) (Fig. 3a) in HER2-high BT-474-bearing mice. Tumor regression was protease activity-dependent, evidenced by the lack of efficacy with HER2-XPAT-NoClvSite versus vehicle. The average body weight of the mice remained generally stable for the experiment duration following dosing with HER2-XPAT proteins or vehicle control (Fig. 3a).

In mice harboring HER2-low HT-55 xenografts, equimolar doses of HER2-XPAT protein (5.1 mg kg^−1^) and unmasked HER2-TCE (0.9 mg kg^−1^) led to significant tumor growth inhibition (TGI) (*P* = 0.013 versus vehicle control for both) (Fig. 3b). HER2-XPAT protein at 2.1 mg kg^−1^ resulted in an intermediate antitumor response (*P* = 0.047 versus vehicle control). All eight vehicle-treated mice developed large tumors >1,000 mm^3^ by day 39, whereas only 38% (3 of 8) and 0% (0 of 8) in the 2.1 mg kg^−1^ and 5.1 mg kg^−1^ HER2-XPAT protein groups, respectively, had tumors >500 mm^3^. HER2-XPAT-NoClvSite had no impact on tumor growth relative to vehicle. The average body weight of the mice remained generally stable during the experiment, following dosing with HER2-XPAT proteins or vehicle control (Fig. 3b).

T-cell activation in BT-474 tumor-bearing mice was assessed on day 18 after treatment with equimolar doses of HER2-XPAT protein and unmasked HER2-TCE. HER2-XPAT protein and unmasked HER2-TCE promoted similar T-cell activation in the tumor microenvironment (TME), indicated by increased expression of activation marker CD25 on both helper and cytotoxic T cells (Fig. 3c). T cells were not activated in peripheral blood where human HER2 is not expressed, consistent with the requirement for HER2 and CD3 dual engagement to activate T cells and redirect their killing.

### In vivo cleavage of XPAT proteins: tumor and healthy organs

**Fig. 4:**
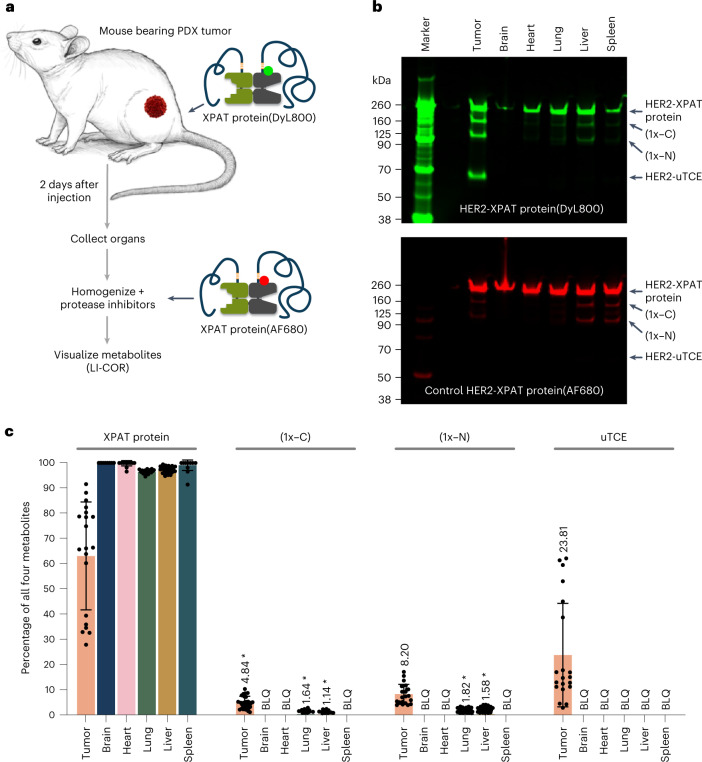
Preferential XPAT protein unmasking in tumor tissue. **a**, Fluorescent dye-labeled HER2-XPAT or EpCAM-XPAT protein was used to track proteolytic cleavage in PDX tumor-bearing mice. **b**, A representative SDS–PAGE gel showing XPAT protein cleavage forms arising in a single PDX-bearing mouse, after imaging by LI-COR Biosciences. Four bands representing HER2-XPAT protein and its three unmasked forms are visible in the tumor sample (green channel) while the other (red channel) predominantly shows the XPAT protein form. **c**, Concentration of XPAT protein forms in tumors and healthy tissues. Results are the mean ± s.d. from 20 mice within one single experiment, with tumors consisting of ten different PDX (Supplementary Table 3); mice were injected with fluorescent dye-labeled HER2-XPAT or EpCAM-XPAT protein. Note that tissues for which ≥14 samples were below the limit of quantification (BLQ) were reported as ‘BLQ.’ Tissues with averages calculated using some samples with BLQ readings are marked with an asterisk. Source data

Two fluorophore-labeled XPAT proteins, XPAT(DyLight 800 (DyL800)) and XPAT(Alexa Fluor 680 (AF680)), were prepared to quantify unmasking in vivo in immunodeficient mice bearing patient-derived xenografts (PDX) (Fig. 4a). Two days after dosing with XPAT protein(DyL800), tumors and other organs were excised and prepared, with XPAT protein(AF680) added to monitor cleavage during sample processing, followed by analysis.

A typical sodium dodecyl sulfate-polyacrylamide gel electrophoresis (SDS–PAGE) analysis of fluorophore-labeled cleavage products from HER2-XPAT is shown in Fig. 4b. Four bands representing HER2-XPAT protein and three unmasked forms are clearly visible in the tumor sample (XPAT protein(DyL800) green channel) while the other (XPAT protein(AF680) red channel) showed minimal unmasking, suggesting limited cleavage ex vivo during sample processing. Limited unmasking was detected in healthy organs, with fully unmasked HER2-TCE undetectable in healthy tissues.

In vivo XPAT protein unmasking was assessed in five PDX tumor models with HER2-XPAT protein and an additional five PDX models with an XPAT prototype targeting human EpCAM (EpCAM-XPAT) (Supplementary Table 3). Unmasked TCE in tumors averaged 23.8% (s.d. 20.6%; s.e.m. 4.6%; range 2.4–61.7%) of all XPAT protein forms; while in healthy tissues, uTCE was below the limit of detection (Fig. 4c). Tumor tissues had substantial levels of partially unmasked XPAT protein, (1x-C) and (1x-N), with lower or undetectable levels in healthy organs.

### Toxicity of HER2-XPAT protein and unmasked HER2-TCE in NHPs

**Fig. 5:**
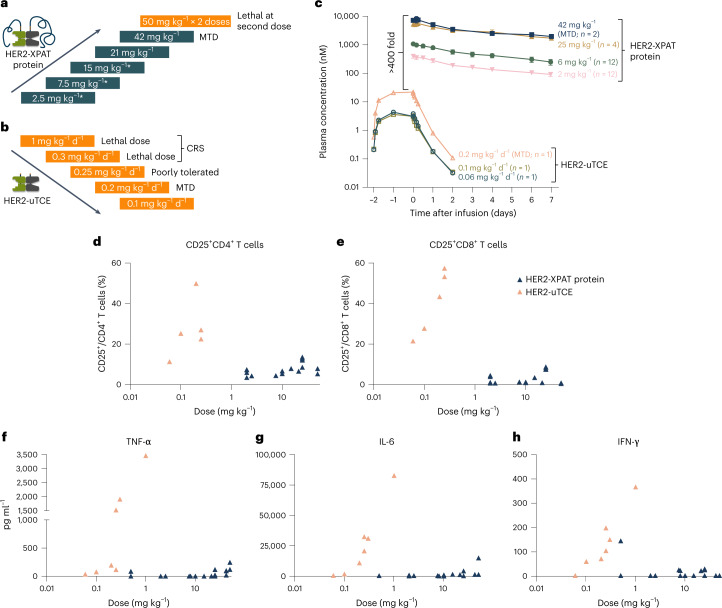
Evaluation of the toxicological, PK and PD characteristics of HER2-XPAT protein and unmasked HER2-uTCE in NHPs. HER2-XPAT protein was administered via short i.v. infusions (∼1–3 h), whereas the unmasked counterpart, due to its short half-life, was administered via 48-h continuous i.v. infusion to provide prolonged systemic exposure. **a**, A dose-escalation study with a single dose of HER2-XPAT protein administered to each cynomolgus monkey. *At doses below 21 mg kg^−1^, a HER2-XPAT prototype was used. **b**, Dose de-escalation study of uTCE in NHPs administered as a 48-h continuous infusion due to its short half-life. **c**, Plasma concentrations in NHPs following administration of a single i.v. dose of HER2-XPAT protein (42 mg kg^−1^, *n* = 2 NHPs; 25 mg kg^−1^, *n* = 4 NHPs; 6 mg kg^−1^, *n* = 12 NHPs; 2 mg kg^−1^, *n* = 12 NHPs) or a 48-h continuous infusion of uTCE (0.2 mg kg^−1^ d^−1^, *n* = 1 NHP; 0.1 mg kg^−1^ d^−1^, *n* = 1 NHP; 0.06 mg kg^−1^ d^−1^, *n* = 1 NHP). Data represent mean plasma concentration (±s.d. when *n* > 3 NHP treated) for the NHPs treated at each dose within one single experiment. **d**,**e**, Activation of circulating CD4^+^ T cells (**d**) and CD8^+^ T cells (**e**) 24 h after drug administration (HER2-XPAT protein, *n* = 16 NHPs; HER2-uTCE, *n* = 5 NHPs). **f**–**h**, Highest plasma levels of TNF-α (**f**), IL-6 (**g**) and IFN-γ (**h**) measured 0–24 h following drug administration (HER2-XPAT protein, *n* = 23 NHPs; HER2-uTCE, *n* = 8 NHPs). Note that the normal ranges for cytokine levels in NHP are as follows: IL-6 0.3–1.2 pg ml^−1^, TNF-α 1–10 pg ml^−1^, IFN-γ 1.1–8.0 pg ml^−1^ (ref. ^44^). Data in **d**–**f** represent a single sample from each NHP receiving the test material within each single experiment. TNF, tumor necrosis factor; IFN, interferon. Source data

HER2-XPAT protein binds to human and NHP HER2 and CD3 with comparable affinities. An NHP dose-escalation study assessed the pharmacokinetics (PK), tolerability and maximum tolerated dose (MTD) of HER2-XPAT protein and its unmasked counterpart. HER2-XPAT protein showed minimal toxicity at a dose ≤42 mg kg^−1^ (defined as the MTD, single dose), with severe toxicity (unrelated to cytokine release syndrome (CRS)) evident at 50 mg kg^−1^ (Fig. 5a). A second dose of HER2-XPAT protein 50 mg kg^−1^ led to toxicity in one of two NHPs dosed, manifesting as multiple findings, including evidence of decreased cellularity in lymphoid tissues, moderate-to-severe colonic inflammation associated with infection with intralesional protozoa and bacterial pneumonia. Lymphoid depletion, resulting in the severe infections, was considered related to HER2-XPAT protein.

No major CRS-associated clinical symptoms or toxicity were seen at any HER2-XPAT protein dose administered (including MTD 42 mg kg^−1^). Low-dose unmasked HER2-TCE (0.3 mg kg^−1^ d^−1^) induced CRS-associated death (Fig. 5b), consistent with literature showing that most TCEs induce significant toxicity in NHPs at doses <1 mg kg^−1^ (ref. ^9^). With HER2-XPAT protein, we observed transient and dose-dependent evidence of decreased lymphocytes, increased neutrophils and small increases in C-reactive protein (CRP) and CD4^+^ T-cell activation in plasma. With HER2-XPAT protein doses ≤42 mg kg^−1^, the only adverse histopathological finding was thymic atrophy. No gross or microscopic findings were found in HER2-expressing tissues, including the heart, where other HER2-targeted agents demonstrated toxicity^15^.

Plasma concentrations over time of HER2-XPAT protein (single intravenous (i.v.) infusion) and unmasked HER2-TCE (48-h i.v. infusion) are shown in Fig. 5c. HER2-XPAT protein demonstrated a biphasic pharmacokinetic (PK) profile, with dose-proportional exposure increases observed across all evaluated dose levels. The estimated half-life of HER2-XPAT protein in a 3-kg NHP was ~3 d, similar to that of other proteins fused to XTEN polypeptides in NHPs^16^. The estimated half-life of unmasked HER2-TCE was ~2 h in a 3-kg NHP. At their MTDs, peak concentrations of HER2-XPAT protein (42 mg kg^−1^) were >400-fold higher than unmasked HER2-TCE (0.2 mg kg^−1 ^d^−1^).

HER2-XPAT protein and unmasked HER2-TCE induce different pharmacodynamic (PD) responses in NHPs (Fig. 5d–h). NHPs receiving unmasked HER2-TCE beyond the MTD indicated overt CRS described with other TCEs^17,18^, including elevated inflammatory cytokines, CRP and blood markers of liver and kidney damage (aspartate transaminase, alanine transaminase, bilirubin and blood urea nitrogen). HER2-XPAT protein, even at the highest doses tested, induced limited systemic T-cell activation (Fig. 5d,e) and cytokine responses (Fig. 5f–h). At its toxic dose (50 mg kg^−1^), HER2-XPAT protein exerted local toxicity and systemic release of cytokines remained low.

### Stability of HER2-XPAT protease-cleavable linker in plasma

**Fig. 6:**
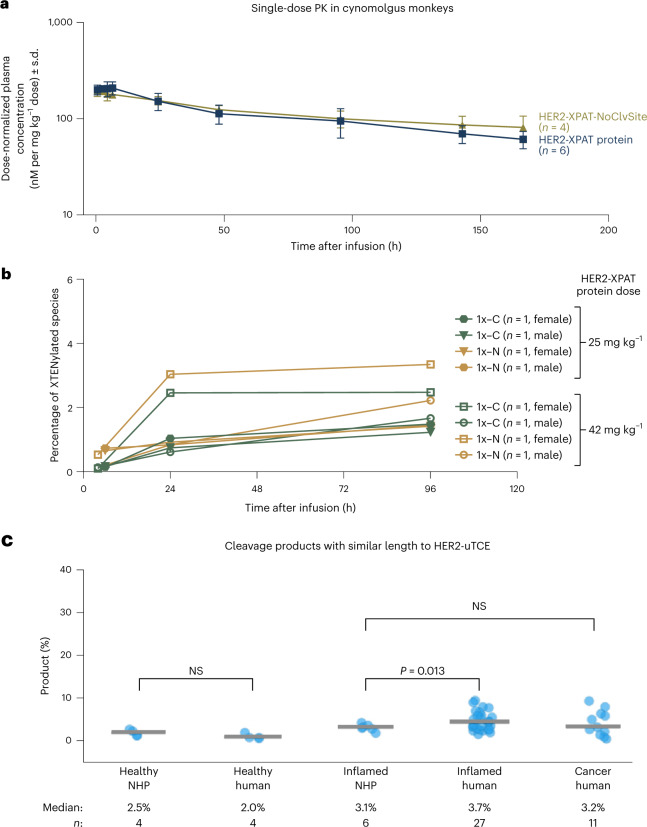
XPAT protein protease-cleavable linkers show minimal cleavage in vivo in healthy NHPs. **a**, Mean plasma concentrations (±s.d.) following a single i.v. administration of HER2-XPAT protein (single plasma samples from *n* = 6 NHPs) and HER2-XPAT-NoClvSite (*n* = 4 NHPs) within one single experiment. **b**, Plasma concentrations of HER2-XPAT(1x−N) and HER2XPAT(1x−C) relative to the entire HER2-XPAT protein, following a single i.v. dose of HER2-XPAT protein (25 mg kg^−1^, *n* = 4 NHPs; 42 mg kg^−1^, *n* = 4 NHPs) within one single experiment. **c**, Quantification of cleavage products with a similar molecular weight to the uTCE generated following incubation of fluorescent-labeled HER2-XPAT protein in plasma from cancer patients (*n* = 11), patients with inflammatory diseases (*n* = 27), healthy donors (*n* = 4) and NHPs (with (*n* = 6) and without (*n* = 4) drug-induced inflammation). The plasma incubation experiment represents a closed system with no clearance mechanisms and will therefore overestimate the accumulation of cleavage products occurring in vivo. Horizontal bars represent median values; dots represent individual observations. *P* values are based on unpaired *t*-tests. Source data

In vivo proteolytic stability was evaluated by comparing the PK profiles of HER2-XPAT protein and HER2-XPAT-NoClvSite in NHPs (Fig. 6a). Concentration–time profiles of HER2-XPAT protein and HER2-XPAT-NoClvSite were similar: dose-normalized area under the curve from day 0 to day 7 (AUC_0–7_) was 757 ± 158 day•nΜ/(mg kg^−1^ dose) for HER2-XPAT protein and 804 ± 79 day•nM/(mg kg^−1^ dose) for HER2-XPAT-NoClvSite. Considerable cleavage of HER2-XPAT protein would accelerate elimination versus its non-cleavable counterpart. The comparable PK curves indicate that HER2-XPAT protein is predominately stable in vivo, with very low cleavage levels at its protease-cleavable linkers in systemic circulation.

After high doses of HER2-XPAT protein (25 mg kg^−1^ and 42 mg kg^−1^), concentrations of partially unmasked metabolites increased over time (Fig. 6b). Molar concentrations of (1x-N) and (1x-C) reached only ≤3.3% and ≤2.5%, respectively, of total material fused to XTEN polypeptides. Fully unmasked HER2-TCE was below the limit of detection (3 nM).

HER2-XPAT protein stability was evaluated in spiked plasma samples from humans (healthy volunteers; patients with cancer or inflammatory disease) and NHPs (healthy monkeys and monkeys with drug-induced systemic inflammation). After 1-week incubation in human plasma (37 °C), fluorophore-labeled HER2-XPAT protein remained predominantly intact. Reduced-length metabolites suggest cleavage occurs at the protease-cleavable linkers and throughout the XTEN mask. Metabolites with molecular weight similar to unmasked HER2-TCE were evident (Fig. 6c). Cleavage products represented only ~2–4% of the spiked fluorophore-labeled HER2-XPAT protein in samples from healthy subjects, cancer patients and patients with inflammatory diseases, respectively. Notably, the metabolite profile was similar following incubation in plasma from humans and NHPs, supporting NHPs as a relevant species for assessing peripheral cleavage and toxicology of HER2-XPAT protein.

### PD and PK evaluation of an EGFR-XPAT prototype

**Fig. 7:**
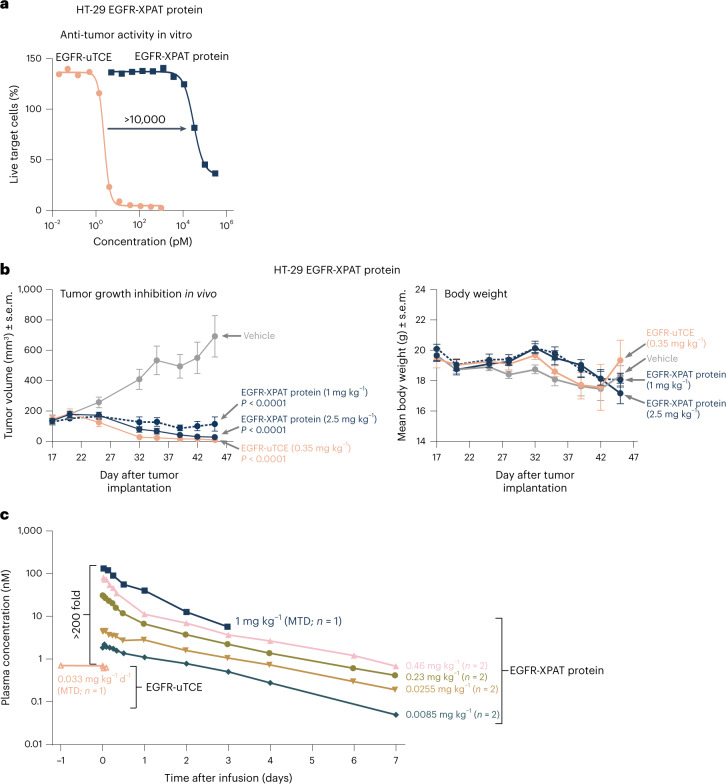
Evaluation of the antitumor activity and *in vivo* PK of EGFR-XPAT prototype. **a**, In vitro activity of the EGFR-XPAT protein and its corresponding unmasked EGFR-TCE (EGFR−uTCE) against HT-29 (*BRAF*^mut^) cells (effector–target ratio, 5:1; *n* = 2 technical replicates at each concentration tested within one single experiment). Extended Data Table 1 provides a summary of EC_50_ values for unmasked EGFR-TCE and EGFR-XPAT protein in the cytotoxicity assays. **b**, TGI in the HT-29 huPBMC-engrafted xenograft model following i.v. administration of EGFR-XPAT protein (TIW dosing starting on day 14 post-tumor implantation; *n* = 6 mice for each concentration tested within one single experiment) and EGFR-uTCE (TIW dosing starting on day 14 post-tumor implantation; *n* = 6 mice for each concentration tested within one single experiment); *P* < 0.0001 for both versus vehicle at day 26 (mixed-effects multiple comparison analyses followed by Tukey’s post hoc test). The average body weight of the mice bearing tumor xenografts remained generally stable for the duration of the experiment following dosing with the EGFR-XPAT proteins or the vehicle control. **c**, PK following a single dose of EGFR-XPAT protein and EGFR-uTCE in NHPs. Sparse data were available for the unmasked EGFR-TCE due to PK assay sensitivity (lower limit of quantification ∼540 pM). Data represent mean plasma concentration based on a single plasma sample collected per time point from each NHP following administration of EGFR-XPAT protein (1 mg kg^−1^, *n* = 1 NHP; 0.46 mg kg^−1^, *n* = 2 NHPs; 0.23 mg kg^−1^, *n* = 2 NHPs; 0.0255 mg kg^−1^, *n* = 2 NHPs; 0.0085 mg kg^−1^, *n* = 2 NHPs) or EGFR-uTCE (0.033 mg kg^−1^ d^−1^, *n* = 1 NHP) within one single experiment. Source data

**Extended Data Fig. 3:**
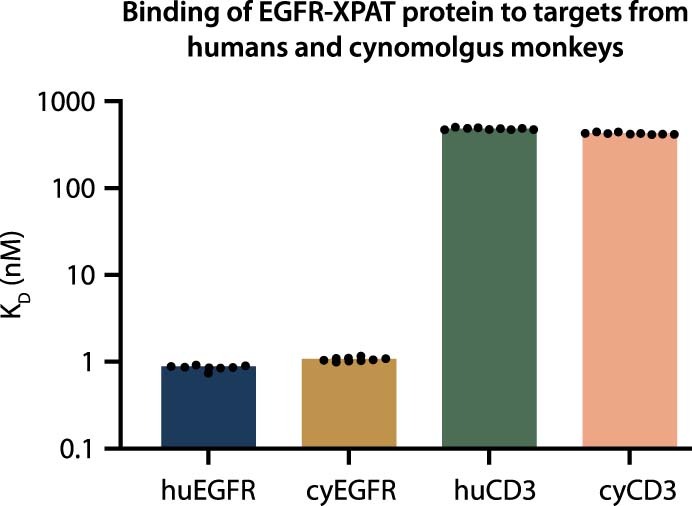
Binding affinity of EGFR-XPAT protein to human and cynomolgus monkey EGFR and CD3. Binding was assessed *in vitro* at 37°C by surface plasmon resonance. Data are K_D_ (*n*=12 technical replicates of a single experiment). Surface plasmon resonance sensorgrams for these data are provided in Supplementary Figs. 19–22. cy, cynomolgus; hu, human; EGFR, epidermal growth factor receptor; XPAT proteins, TCEs fused to XTEN polypeptides. Source data

An EGFR-XPAT prototype was generated based on the variable sequences of panitumumab and humanized SP34 (CD3-binding domain). EGFR-XPAT protein binding affinities suggest cross-reactivity between EGFR and CD3 from humans and cynomolgus monkeys (Extended Data Fig. 3). Strong masking of in vitro T-cell killing by >4 logs was observed with EGFR-XPAT protein versus unmasked EGFR-TCE (Fig. 7a), with XTEN polypeptide masking showing similar protection as with HER2-XPAT. EGFR-XPAT protein showed potent in vivo antitumor activity in huPBMC-engrafted mice bearing HT-29 (*BRAF*^mut^) human colorectal tumors (Fig. 7b).

In NHPs, EGFR-XPAT protein 0.46 mg kg^−1^ (single i.v. dose) triggered minor toxicity and cytokine release; 1 mg kg^−1^ (MTD) induced a much stronger response (Fig. 7c). The MTD of unmasked EGFR-TCE (48-h continuous i.v. infusion) was 33 μg kg^−1 ^d^−1^; a 1-h i.v. infusion of 8 μg kg^−1^ followed by continuous infusion of 66 μg kg^−1 ^d^−1^ (terminated after 25 h) resulted in severe CRS-associated toxicities. At their MTDs, EGFR-XPAT protein (1 mg kg^−1^) had a ≥ 200-fold higher C_max_ versus unmasked EGFR-TCE (0.033 mg kg^−1^), demonstrating that XTEN masks can improve the safety margin (and potentially widen the therapeutic window) for TCEs targeting broadly expressed targets.

## Discussion

We developed bispecific TCEs with protease-releasable masking technology (XTEN polypeptides) to create XPAT proteins, precision-activated TCEs designed for preferential activation in solid tumors versus healthy tissues. XTEN masks reduce binding of unmasked TCE to the CD3 and TAA targets via steric hindrance and prolong the masked TCE half-life. Proteolytic unmasking restores potency and the short half-life in vivo of the unmasked TCE, minimizing systemic exposure risk should cleavage occur outside the tumor or if the unmasked TCE diffuses away from the TME. Fully masked XPAT proteins provided ≤4-log protection against TCE cytotoxicity in vitro versus their unmasked TCE counterparts. Mouse xenograft studies confirmed that XPAT proteins penetrate tumors, where they are preferentially unmasked. Exposure to fully cleaved, active uTCE was limited in the healthy tissues of mice injected with XPAT proteins. The anti-TAA and anti-CD3 scFvs in the XPAT protein do not cross-react with the mouse analogs. In mouse models, human TAAs were only present in the transplanted tumor tissue, not in healthy mouse tissue. The in vivo localization of XPAT proteins could differ in human patients. The XPAT protein anti-TAA and anti-CD3 scFvs cross-react with their targets in NHPs, so the toxicology of XPAT proteins was evaluated in NHPs. In NHPs, the presence of the masks on TCEs provided a strong safety margin, showing 200- and 400-fold increases in the tolerated C_max_ at the MTDs of HER2-XPAT and EGFR-XPAT proteins relative to their unmasked TCE forms, respectively. These are impressively wide predicted safety margins in a relevant toxicology species, with HER2 having more potential given a more limited expression profile in normal tissues versus EGFR. Clinical studies are needed to understand whether these safety margins are adequate for HER2 and EGFR. XPAT proteins demonstrated high proteolytic stability, with minimal cleavage in plasma in vitro and in vivo in humans and NHPs. XPAT proteins show potential to substantially widen therapeutic index by retaining antitumor efficacy with marked reduction of toxicity, a major barrier for unmasked TCEs in clinical development.

Progress applying bispecific TCEs as treatments for solid tumors was hampered by on-target, off-tumor toxicity in healthy tissues expressing even low levels of TAA^1^. Ertumaxomab (non-humanized HER2-TCE) demonstrated two partial responses and one complete response, with an MTD of 100 μg QW for three doses^19^. CRS symptoms were dose-limiting and formation of anti-drug antibodies hampered further development^19^. Another HER2-TCE in clinical development, GBR 1302, was also associated with dose-limiting CRS at doses ≥100 ng kg^−1^ (ref. ^20^).

Application of XTEN polypeptide masks to improve the half-life and therapeutic index of a TCE is based on a clinically validated approach. XTEN masks were initially engineered as an alternative to PEGylation for extending the half-life of biologics^11,16,21,22^. The utility of XTEN polypeptides (without a protease-cleavable linker) for prolonging plasma half-life and pharmacologic effects was demonstrated with growth hormone-XTEN and exendin-4-XTEN in preclinical and clinical studies^23–25^. The utility of XTEN polypeptide masks with protease-cleavable linkers was demonstrated for FVIII and factor IX^26,27^. Both XTEN polypeptide fusion products contain sequences cleaved by a protease during coagulation activation. A phase 3 clinical trial (XTEND-1; ClinicalTrials.org identifier NCT04161495) suggests that efanesoctocog alfa (recombinant FVIII Fc-von Willebrand factor-XTEN) has a class-leading plasma half-life in patients with hemophilia A^13^. The low immunogenicity potential of XTEN masks was confirmed after ≤3 years of treatment with growth hormone-XTEN in children with growth hormone deficiency^14,28^.

With TCEs, the cross-linking of TCR-drug complexes by anti-drug antibodies (ADAs) can stimulate receptor activation and cytokine release^29^. TCEs designed with affinity-based masks contain blocking peptides that mask the antibody-binding sites^30,31^. These affinity-based masks utilize blocking domains uniquely developed for each binding domain and each has a unique risk for eliciting an immune response. The low immunogenicity of the XTEN polypeptide-masked XPAT protein may reduce the risk of ADA formation. The hydrophilic, unstructured nature of the XTEN polymer minimizes interactions with other proteins, including the peptide-binding pockets of MHC class II molecules^32,33^, supporting reduced immunogenicity risk.

**Fig. 8:**
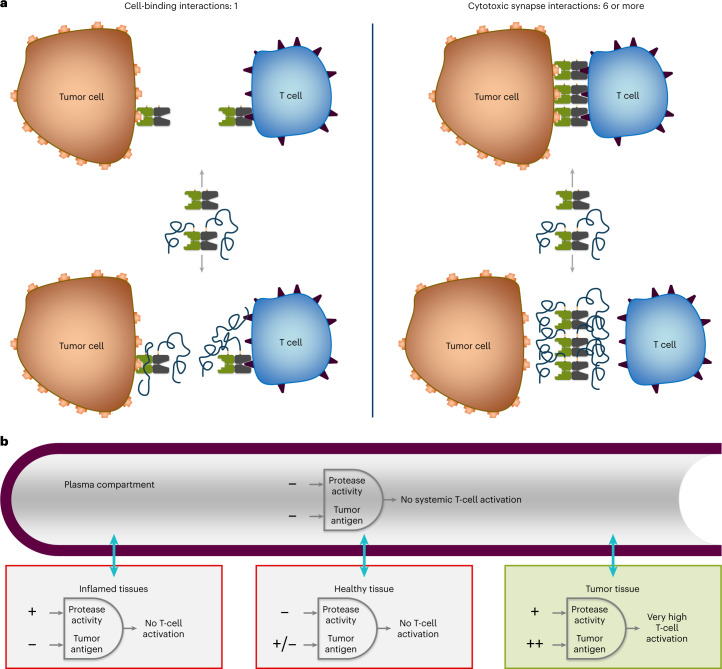
Regulation of XPAT protein activity. **a**, Mechanism for XPAT protein binding to cells (left) and promoting T-cell engagement. Binding of XPAT protein to cells is a bimolecular event in which equilibrium should be directly proportional to the binding affinities of the component molecules. In contrast, synapse formation (right) is a high-order molecular process that involves many individual binding events. **b**, Proposed mechanisms for differential regulation of XPAT protein activity in different tissue compartments are depicted. By harnessing the potency of bispecific TCEs with an enhanced therapeutic window, XPAT proteins may provide an opportunity to improve clinical outcomes beyond those achieved with TAA-targeted monoclonal antibodies or antibody–drug conjugates, enabling patients to safely mobilize T cells independent of their antigen specificity.

There was an apparent discordance in the degree of protection provided by XPAT proteins in reducing target-directed cytotoxicity versus the lesser impact of XTEN polypeptide masking on XPAT protein binding to each target. In vitro HER2-XPAT protein studies show that XTEN polypeptide masks reduced cytotoxic activity (>4-logs reduction in BT-474 tumor cells) by a much larger magnitude than their effect on target binding (tenfold and sixfold reduction in affinity for human HER2 and CD3, respectively). In the absence of proteolytic cleavage, the functional masking is associated with even greater (>4 logs) attenuation of cytotoxic activity versus uTCE, as seen when protease cleavage sites are removed from HER2-XPAT protein. This discordance may be explained by the multivalent nature of the immune synapse that triggers T-cell activation and cytotoxicity (Fig. 8a). In binding experiments, the equilibrium between bound and unbound forms should be directly proportional to the association/dissociation rate constants (Fig. 8a). Mediation of cytotoxicity requires ligand-driven coalescence of ≥3–10 TCRs to form a competent immune synapse between a T cell and target cell^34,35^. Formation of each bridge is likely hindered by the XTEN polypeptide mask and compounded by each TCR-bound XPAT protein on the T-cell membrane. The minimal number of engaged TCRs required for activation also explains why unmasked TCEs with modest affinities in the nanomolar range can have EC_50_ values in the single-digit picomolar range, as confirmed by the binding and cytotoxicity data with unmasked HER2-TCE and unmasked EGFR-TCE.

XPAT proteins were designed as ‘AND’ gates that require two orthogonal triggers for activity (Fig. 8b). If a TAA is expressed in healthy tissue, T-cell activation should be minimal as protease activity is likely to be low. In inflamed non-cancerous tissue with elevated extracellular protease activity, XPAT protein activity is also expected to be low due to low TAA expression. In principle, tumor tissue should uniquely meet both conditions for XPAT protein activity: high TAA expression and high extracellular protease activity.

Our results support that XPAT proteins function as ‘AND’ gates. HER2-XPAT protein efficacy depended on cleavage at its protease release site and was associated with intratumoral T-cell activation. Circulating HER2-XPAT protein remained proteolytically stable and T cells remained quiescent. Preferential unmasking of fluorophore-labeled HER2-XPAT and EpCAM-XPAT proteins was evident in tumors 48 h after administration to BT-474 and PDX tumor-bearing mice versus heart, brain, liver and spleen tissues. This supports the key tenets of localized protease dysregulation in tumors and the dominance of protease inhibition in normal tissues.

HER2-XPAT protein and EGFR-XPAT prototype showed robust safety margins versus their unmasked TCE counterparts in NHPs. XPAT protein unmasking in circulation and normal tissues resulted in limited quantities of the (1x-C) and (1x-N) intermediates in plasma, with fully unmasked TCE below the level of detection. Overt CRS was not seen with HER2-XPAT protein, even at (or above) the 42 mg kg^−1^ MTD. Unmasked HER2-TCE at its MTD (0.2 mg kg^−1^) resulted in high plasma cytokine levels, with CRS-associated death occurring with a low dose (0.3 mg kg^−1^). Plasma stability data for HER2-XPAT protein suggest that leakage of active proteases from tumors in patients is unlikely to result in systemic unmasking of HER2-XPAT protein.

In conclusion, XPAT proteins are TCEs preferentially activated in the TME to drive potent tumor cell killing while minimizing on-target, off-tumor toxicity damaging to healthy tissues or causing CRS. By harnessing the potency of bispecific TCEs with an enhanced therapeutic window, XPAT proteins may provide an opportunity to improve clinical outcomes beyond those achieved with TAA-targeted monoclonal antibodies or antibody–drug conjugates, enabling patients to safely mobilize T cells independent of their antigen specificity. The adaptability of the XPAT protein format for targeting different TAAs was demonstrated by the EGFR-XPAT prototype, which showed potent in vivo efficacy in the HT-29 xenograft model while using the same protease-cleavable linkers and XTEN masks as HER2-XPAT protein. A clinical study of HER2-XPAT protein (ClinicalTrials.org identifier NCT05356741) has been initiated to evaluate XPAT technology in humans. Additional XPAT proteins are being designed to address the unmet need for targeted immunotherapy across different solid tumors.

## Methods

The studies reported here were conducted to characterize the specificity and binding characteristics of HER2-XPAT protein and an EGFR-XPAT prototype (and their metabolites), as well as their PK, PD and toxicity profiles in vivo in mice and NHPs. Further information on research design is available in the Nature Portfolio Reporting Summary linked to this article. Informed consent was obtained from all human research participants.

### Ethical regulations and animal studies

All studies involving animals were conducted at specialist research centers, which were accredited by the Association for Assessment and Accreditation of Laboratory Animal Care. The study protocols were approved by The Institutional Animal Care and Use Committees (IACUC) of BioDuro Sundia (IACUC protocol FFS-001), Crown Bioscience (IACUC protocol AN-1903-05-1759) and Explora BioLabs (IACUC protocol EB17-010).

All mice used were female, aged 6–8 weeks and housed in microisolator cages with a maximum of five mice per cage. For tumor xenograft experiments, the maximal tumor size/burden was prespecified as 2,000 mm^3^; if the tumor burden reached this maximal volume, the individual animal was humanely killed. The genetic strains and source of the mice used are specified below in the descriptions of experimental methods.

Single-dose toxicokinetic studies evaluating XPAT proteins and their equivalent unmasked TCEs were conducted in NHPs at specialist centers (Charles River Laboratories; WuXi AppTec (Suzhou)). Experimentally naive male and female cynomolgus monkeys of Cambodian or Chinese origin were used in the studies. The age of the animals at the initiation of dosing was between 35 and 43 months. The animals were acclimated to laboratory housing for at least 7 d before the initiation of dosing. Housing setup was as specified in the USDA Animal Welfare Act (Code of Federal Regulations, Title 9) and as described in the Guide for the Care and Use of Laboratory^36^.

### Design and production of XPAT proteins

A series of XPAT protein prototypes were evaluated in these studies, which targeted HER2, EGFR and EpCAM. HER2-XPAT protein includes a tumor-binding anti-HER2 domain that is based on the variable domains of pertuzumab, a domain that has been extensively studied in clinical trials^37^. The CD3-binding domain of HER2-XPAT protein is based on the sequence of SP34 (ref. ^38^), which is the parent domain for many TCEs that are currently undergoing clinical evaluation^8^. The CD3-binding domain in HER2-XPAT protein is a proprietary variant of SP34 that was engineered for increased stability and reduced affinity for CD3.

The clinical candidate HER2-XPAT protein contains XTEN mask lengths of 306 and 586 residues at its N and C termini, respectively. The XTEN mask lengths include an N-terminal polyhistidine tag, a C-terminal E-P-E-A tag (C-tag), as well as the portion of the release site undergoing protease cleavage. HER2-XPAT protein contains two identical protease-cleavable linkers that were optimized for cleavability by multiple proteases, including matrix metalloproteinases (MMPs), the serine proteases urokinase plasminogen activator and matriptase and the cysteine protease legumain. The length of the released XTEN mask varies slightly depending on which class of protease releases the mask.

An EGFR-XPAT prototype was designed to incorporate the variable sequences of humanized SP34 and the anti-EGFR monoclonal antibody panitumumab. Some of the in vivo unmasking studies in PDX models utilized an EpCAM-XPAT prototype due to the high prevalence of EpCAM expression in human carcinomas. An EpCAM-XPAT prototype was engineered based on the EpCAM-binding scFv described previously^39^.

### Production of XPAT products in *Escherichia coli*

XPAT proteins were expressed in *E.* *coli*, which was transformed with the expression vector and grown in fermentation. Fermentation cultures were grown with animal-free complex medium at 37 °C and temperature shifted to 26 °C before phosphate depletion, which triggered induction (PhoA). Target protein was partitioned into the periplasm via an N-terminal secretory leader sequence (MKKNIAFLLASMFVFSIATNAYA), which was cleaved during translocation. During collection, fermentation whole broth was centrifuged to pellet the product-containing cells, which were retained and frozen at ≤−70 °C. The frozen cell pellet was resuspended and, once homogenous, the resuspension was mechanically lysed. The chilled flocculate was centrifuged (12,000 RCF, 10 °C, 30 min) and the supernatant was decanted and retained, while the pellet was discarded. The following day, centrifugation was performed again (12,000 RCF, 10 °C, 30 min) and the supernatant was decanted, submicron filtered and purified via a chromatographic process comprising an Anion Exchange (AEX) chromatography step.

XPAT proteins and their derivatives were prepared as aqueous solutions and stored frozen at ≤−70 °C and, after thawing, at temperatures between 2 °C and 8 °C.

### Kinetic analysis using surface plasmon resonance

Kinetic studies were performed by surface plasmon resonance at 37 °C to determine binding *K*_D_ of HER2-XPAT protein and its metabolites to human and cynomolgus monkey HER2 and CD3.

A Biacore 4000 SPR biosensor (Cytiva) was used to measure binding kinetics of HER2-XPAT protein and its metabolites to the Fc-tag of the target ligands, human and cynomolgus monkey HER2 and CD3. Data were collected by capturing each ligand at four surface densities onto a Protein A sensor chip, prepared by coupling Protein A to ∼1,000 response units onto a C1 (flat) sensor chip using standard activation with *N*-hydroxysuccinimide/(1-ethyl-3-(3-dimethylamino) propyl carbodiimide. Protein A was diluted to 50 μg ml^−1^ into 10 mM NaAc pH 4.5 and injected for 300 s. Surfaces were blocked with a 300-s injection of 1 M ethanolamine.

Ligands and test samples were diluted in running buffer (Dulbecco’s PBS containing 1% bovine serum albumin and 0.05% surfactant p20, pH 7.4). Human and cynomolgus HER2-Fc and CD3ε-Fc samples were diluted to 100 nM as the highest concentration and captured in a threefold dilution series over four sensor spots, resulting in capture densities ranging from ∼100 to ∼800 resonance units. Test samples of HER2-XPAT protein and metabolites were diluted in a threefold titration series up to 1 μM. Response data for each sample were globally fitted to a 1:1 interaction model to determine binding kinetics.

Binding affinities of EGFR-XPAT protein were measured by using Biacore T200 (Cytiva). C1 chip (Cytiva) was immobilized with ProA (Thermo Fisher) with response ≤150 response units. EGFR-XPAT protein was titrated in threefold from 37 nM to 1.37 nM and 1,000 nM to 1.37 nM against CD3ε and EGFR, respectively. The study was performed at 37 °C in a running buffer (PBS-P with 0.1% BSA) with association and disassociation times of 60 s and 360 s, respectively.

### In vitro cytotoxicity assays

huPBMCs from healthy donors were used as effector cells to determine the in vitro cytotoxicity of XPAT protein and its metabolites. huPBMCs were isolated from either whole blood or lymphocyte-enriched buffy coat preparations (obtained from BioIVT).

Human tumor cell lines (American Type Culture Collection; ATCC) were cultured in recommended growth medium containing 10% fetal bovine serum (FBS) and l-glutamine at 37 °C in 5% CO_2_ in a humidified incubator.

For HER2-XPAT protein, target cells were the high HER2-expressing tumor cell lines, BT-474 (ATCC HTB-20) and SKOV3 (ATCC HTB-77) and the medium-low HER2-expressing cell line MCF7 (ATCC HTB-22). A total of 20,000 tumor cells were incubated overnight in 96-well, flat, clear-bottom plates. huPBMCs were thawed, rested overnight and then added to the assay plates at an effector–target ratio of 1:1 (except for assays with HER2-XPAT-NoClvSite, in which the effector−target ratio was 5:1), followed by serially diluted HER2-XPAT protein or its metabolites.

For in vitro cytotoxicity assays, huPBMCs and the HT-29 (*BRAF*^mut^; ATCC HTB-38), with an effector–target ratio of 5:1, were incubated for 48 h. Co-cultures were treated with increasing concentrations of the EGFR-XPAT prototype or unmasked EGFR-TCE.

After a 48-h incubation with XPAT protein, cells were washed and CellTiter-Glo luminescent substrate solution was added to quantitate the number of viable cells present in the wells. The mean of the signal from all non-treatment wells was calculated and used to determine the percentage of live cells from treatment wells ((Treatment Signal/Mean of Non-Treatment Signal) × 100 = % live). The percentage of live target cells was plotted by concentration.

### HER2 receptors on tumor cell lines: flow cytometry

A phycoerythrin (PE)-labeled detection antibody against HER2 (CD340 (erbB2/HER2) antibody, BioLegend, 324406) was prepared at different dilutions in fluorescence-activated cell sorter (FACS) buffer. A diluted detection antibody of 100 μl was added to 1 × 10^6^ tumor cells and incubated on ice for 45 min at 4 °C, after which the cells were washed and centrifuged (1,200 r.p.m., 5 min). A tube of BD QuantiBRITE PE beads (PE Fluorescence Quantitation kit, 340495, BD Biosciences) was reconstituted in 0.5 ml FACS buffer and run on a Cytoflex flow cytometer (Beckman-Coulter) to determine setting thresholds for forward and side scatter. The stained tumor cells were acquired using the same settings and 10,000 events were collected. Linear regression analysis was used to determine receptor density of the cell lines from the geometric mean fluorescence intensity of the bead populations.

### In vitro activation of *NFAT*-luciferase Jurkat reporter cells

The activation of CD3^+^ Jurkat T cells (Promega, J1625, J1601) by HER2-XPAT protein and unmasked HER2-TCE was assessed. Jurkat T cells genetically engineered to express a luciferase reporter driven by an *NFAT*-response element were used to determine the level of T-cell activation via measurement of luciferase present in the wells after treatment. HER2-expressing BT-474 tumor target cells (20,000 cells per well) were incubated overnight at 37 °C, in 5% CO_2_. Control wells with only cell medium were included to assess activation of Jurkat cells in the absence of HER2-expressing cells. Before the end of the overnight incubation, serial dilutions of HER2-XPAT protein and its proteolytic metabolites were prepared, including a non-treatment control. Jurkat cells were seeded at an effector−target ratio of 5:1. After 6 h incubation, Bio-Glo Luciferase (Promega, G7940) substrate solution was added to quantify the number of viable Jurkat cells present.

### Human tumor cell line xenograft models

Mice were maintained under a 12-h light/dark cycle and kept in individual ventilation cages at a temperature of 24 ± 2 °C and humidity of 55 ± 10% with a maximum of five animals per cage. Animal viability and behavior were observed daily. Body weights were measured twice a week. The length and width of the tumor were measured twice a week with calipers. Individual mice were terminated if the tumor size reached the 2,000 mm^3^ humane limit, or if a tumor was ulcerated, necrotic, bleeding or impaired the nutrition or health of the mice. Other clinical signs of animal stress, illness or impaired mobility were additional end points requiring euthanasia.

For the BT-74 model, non-obese diabetic (NOD)/Shi^scid^ IL2rgamma^null^ (NOG) mice (Taconic) were inoculated s.c. with 2 × 10^7^ BT-474 tumor cells in 200 μl RPMI 1640 culture medium with Matrigel (1:1) on day 0. On day 6, mice were engrafted i.v. with 1 × 10^7^ huPBMCs. Treatment with HER2-XPAT protein or one of its metabolites began when MTV reached 185 mm^3^. The test substances were injected via the tail vein at equimolar doses QW for 3 weeks.

For the HT-55 model, 7−8-week-old NOD-Prkdc^scid^Il2rg^null^ (NPSG; Phenotek Biotechnologies) mice were inoculated s.c. with 5 × 10^6^ HT-55 tumor cells (Cobioer, CBP60012) and engrafted with 1 × 10^7^ huPBMCs on day 6. On day 10 (at MTV of ∼130 mm^3^), mice were injected via the tail vein with HER2-XPAT protein (QW), unmasked HER2-TCE (TIW; due to its shorter half-life) and HER2-XPAT-NonClv (QW), with dosing continued for 4 weeks.

For the HT-29 model, female 6–8-week-old NOG mice were inoculated s.c. with 1 × 10^7^ HT-29 tumor cells and engrafted with 1 × 10^7^ huPBMCs i.v. on day 15. Once tumors reached ∼150 mm^3^, test articles were administered i.v. via the tail vein, either QW or TIW (as indicated in Results) for 3 weeks.

### T-cell activation in HER2-high BT-474 human tumor xenografts

T-cell activation was assessed in NOG mice with HER2-high BT-474 xenografts, which underwent similar procedures as described in the previous section and were treated with equimolar doses of HER2-XPAT protein (2.1 mg kg^−1^) QW and unmasked HER2-TCE (0.9 mg kg^−1^) TIW. Mice were killed on day 18 following dosing (day 40 following tumor inoculation) to measure T-cell activation in tumors and blood by flow cytometry. Tumors and blood were collected and processed to measure surface activation markers CD25 and CD69 on CD4^+^ and CD8^+^ T-cell subsets by flow cytometry.

Blood was collected from mice by intracardiac puncture, as a terminal procedure under deep isoflurane gas anesthesia, into collection tubes with anticoagulant (K_2_-ethylenediaminetetraacetic acid (K_2_EDTA)) for flow cytometry analysis. Excised tumors were dissected into smaller fragments using scalpels, further dissociated into single cell suspensions in a non-enzymatic cell dissociation buffer, incubated at 37 °C for 30 min and mechanically separated through a 70-µm cell strainer. Viable cells were then enriched using Ficoll-based gradient centrifugation.

Blood (50 µl if possible) was directly distributed per staining well per tube. Before staining, red blood cells were lysed with Versalyse lysing buffer. Before cell-surface staining, cell suspensions were enumerated and one million cells (when possible) were distributed per well per tube. Cells were stained in a final staining volume of 100 μl per well (unless specified).

The first step performed was staining with a viability dye to allow dead cell exclusion at the analysis. Nonspecific binding was minimized using the Fc receptor blocking reagent. Fluorescence-labeled surface target antibodies were added, according to the procedure described by the supplier for each antibody. The mixture was incubated for 20 min at room temperature protected from light, washed and then fixed with 200 µl 1% formaldehyde in PBS containing PKH26 beads. All samples were stored at +4 °C and protected from light until acquisition on cytometer.

For identification of positive and negative populations, the fluorescence minus one (FMO) principle was used to account for background antibody fluorescence. FMO controls were used for controls, for each organ, using mice from group 0 (residual mice). Compensation was performed using compensation beads and/or single stained cells.

Details about the antibodies used for flow cytometry are included in the Reporting Summary and Supplementary Fig. 1 shows the gating strategy applied to each panel in the flow cytometry immunophenotyping analyses in mice.

### Evaluation of in vivo unmasking of XPAT proteins

Variants of the HER2-XPAT and EpCAM-XPAT proteins were constructed containing an additional unpaired cysteine between the TCE and the C-terminal protease release site. Maleimide derivatives of fluorescent dyes, either AF680 or DyL800, were conjugated to the XPAT protein variants with a stoichiometry of ∼1:1. Limited proteolysis of HER2-XPAT protein(DyL800) showed the expected metabolites on SDS–PAGE gel, with the same bands detected by Coomassie Blue staining and the LI-COR Odyssey CLx Imager (with Image Studio Lite v.2 software; data not shown). Dye-labeling had a negligible impact on the rate of HER2-XPAT protein cleavage by MMP-9 (data not shown).

XPAT protein(DyL800) was injected into mice bearing PDX via the tail vein (these animal studies were performed at Champions Oncology). The details of the PDX models evaluated are provided in Supplementary Table 3. After 2 d, mice were perfused with PBS via the heart. Tumors and organs (brain, heart, lung, liver and spleen) were then collected and homogenized in the presence of protease inhibitors and the XPAT protein(AF680)-labeled internal control (red).

### Fluorophore-labeled XPAT metabolites in mice bearing PDX

Collected mouse organs and tumor tissue were weighed, thawed and homogenized using an approximate ratio of 1:5 tissue mass:volume homogenization buffer. Homogenization was performed in a Precellys Evolution with Cryolys, using CKMix lysing tubes (Bertin Corp) at 3 × 5,800 r.p.m. for 30 s with a 15-s pause. Insoluble material was cleared by centrifugation at 15,000*g* for 10 min and protein extracts were taken from the supernatant.

Clarified protein extracts were diluted 1:10 in radioimmunoprecipitation buffer (Thermo Fisher Scientific), mixed with 4× Protein Sample Loading Buffer (LI-COR) to a final concentration of 1× and run on NuPAGE 4‒12% Bis-Tris protein gels in NuPAGE MES running buffer (Thermo Fisher Scientific). A six-point standard curve was run on each gel containing XPAT protein(DyLight 800 (DyL800)) and XPAT protein(Alexa Fluor 680 (AF680)) using a fivefold dilution series from a concentration of 1,000 pM to 0.32 pM. Gels were directly scanned on a LI-COR Odyssey CLx Imager using automatic image intensity (Image Studio Lite v.2 software).

Bands corresponding to the XPAT protein and its metabolites, XPAT(1x-C), XPAT(1x-N) and uTCE were quantified using a weighted linear regression, which used a weighting formula of 1 / *Y*^2^ against the concentration of the known full-length standards. To account for unmasking during processing, the XPAT protein(AF680)-labeled internal control was used to calculate a correction factor for the full-length XPAT protein and for each metabolite. The calculated concentrations were multiplied by the respective correction factors and the lower limit of quantification was applied to the calculated concentrations after the correction factor. Relative percent unmasking was calculated using the concentrations of each metabolite as a percentage of total XPAT protein-derived material present in each organ. Averages for tissues with <14 samples BLQ were calculated by using the lower limit of quantification concentration for the BLQ samples. Thus, the actual average concentration may be even lower than shown in the graph. Tissues with averages calculated using some samples with BLQ readings are marked with an asterisk.

### Dose-escalation and toxicokinetic single-dose studies in NHPs

For a single-dose-escalation study in NHPs, HER2-XPAT protein was administered as a single 1-h i.v. infusion on study day 1 (PK day 0). Unmasked HER2-TCE was administered as a 48-h continuous i.v. infusion through a surgically implanted catheter inserted into a femoral vein. For HER2-XPAT protein studies, an HER2-XPAT prototype was used for initial dose levels (≤15 mg kg^−1^); the clinical candidate HER2-XPAT protein was used for doses ≥21 mg kg^−1^.

Animals were observed during the 1-week period following dosing. Study end points were clinical signs, body weight, food consumption, body temperature, clinical pathology parameters (hematology and clinical chemistry), creatine kinase isoenzyme analysis, cytokines, immunophenotyping, gross necropsy findings and histopathological examination.

Blood for PK analysis was collected into tubes with anticoagulant sodium heparin or K_2_EDTA. Test article concentrations were measured in plasma using an electrochemiluminescence immunoassay. Raw data were analyzed using a weighted(1 / signal^2^) four-parameter logistic non-linear regression curve fit on SoftMax Pro v.7.0 data analysis software.

Blood for cytokine analysis was collected into tubes with anticoagulant sodium heparin or K_2_EDTA. Concentrations of TNF-α, IL-6 and IFN-γ were measured in plasma using a magnetic bead-based multiplex assay for the Luminex platform on the MAGPIX system. Raw data were analyzed using a weighted (1 / signal^2^) five-parameter logistic non-linear regression curve fit on xPONENT v.4.3.229.0 data analysis software.

Changes in immune profiles in peripheral blood were analyzed by flow cytometry in blood collected pre-dose and at 24 h and 48 h after infusion. Further details of the flow cytometry methods are below.

### Quantitative western blots in NHP plasma

The concentration of the singly cleaved metabolites, HER2-XPAT(1x-C) and HER2-XPAT(1x-N), in the plasma of NHPs administered HER2-XPAT protein was measured using a quantitative western blot method. A sample of 10 μl, standard (known concentrations of HER2-XPAT(1x-C) and HER2-XPAT(1x-N) diluted in plasma) or quality control in plasma was combined on ice with 20.0 μl of PBS with 2× concentration of protease inhibitors (HALT Protease Inhibitors, Pierce 78438). Ten microliters of 4× NuPAGE LDS (Invitrogen NP0007) with 50 mM dithiothreitol was added to each diluted sample on ice and thoroughly mixed. For SDS–PAGE, 5 μl of the final mixture was loaded to each lane of a 26-well NuPAGE 4–12% Bis-Tris Midi protein gel (Invitrogen WG1403A). The gel was run at 200 V for 50 min. SDS–PAGE gels were incubated in NuPAGE Transfer Buffer (Invitrogen NP00061) before blotting onto nitrocellulose membranes using the Invitrogen iBlot 2 system (Invitrogen IB23001). The nitrocellulose membranes were then dried, re-hydrated with Tris-buffered saline (TBS) and blocked using LI-COR Protein-Free Blocking Buffer (LI-COR 927-80001). A monoclonal mouse antibody recognizing the anti-HER2 scFv (aaHER2-M7) was conjugated to XPAT protein(DyL800) (XPAT tagged with the fluorescent dye DyL800) for detection of HER2-XPAT protein metabolites. The detection antibody was prepared in LI-COR Intercept T20 (TBS) antibody diluent (LI-COR, 927-650001) at 0.1 μg ml^−1^. The blocked blots were briefly rinsed with TBS before incubating with 35 ml detection antibody at room temperature, rocking and protected from light. Blots were thoroughly washed with TBS containing 0.1% polysorbate 20 and rinsed with TBS before scanning on the LI-COR Odyssey CLx. Image Studio Lite (with Image Studio Lite v.2 software) was used to quantitate relative fluorescence signal for the HER2-XPAT(1x-C) and HER2-XPAT(1x-N) bands. Relative fluorescence signal for standards were processed in SoftMax Pro using a four-parameter logistic standard curve regression with 1 / *Y*^2^ weighting to establish a dose–response curve. HER2-XPAT(1x-C) and HER2-XPAT(1x-N) concentrations for quality control and study samples were determined by interpolation to the dose–response curve. Samples with concentrations below the lowest standard point of 7.81 nM were reported as BLQ.

### Flow cytometry: immune profiles in NHPs

Changes in immune profiles in peripheral blood were analyzed by flow cytometry in the single-dose toxicokinetic studies evaluating XPAT proteins and their equivalent unmasked TCEs in NHPs. Blood was collected pre-dose and at 24 h and 48 h after infusion. The cellular antigens and cell populations shown in Supplementary Table 4 were quantified, using specific antibodies against the marker antigens.

Blood samples were incubated with blocking buffer (CRL, Envol Biomedical), labeled with fluorochrome-labeled surface antibodies (Reporting Summary). Red blood cells were lysed and, after centrifugation the cell pellet was resuspended in FBS-containing buffer. Centrifugation and resuspension in FBS-containing buffer was repeated. The samples were mixed, the cell suspension was transferred to a 96-well v-bottom plate and acquired utilizing the BD FACSCanto II flow cytometer and BD FACSDiva software v.9.0.

Absolute lymphocyte counts for each cell subset population were derived by applying the values derived from a hematology analyzer to the relative percentage output from the flow cytometer.

The gating strategy for the flow cytometry analyses is shown in Supplementary Fig. 2.

### Stability of HER2-XPAT protease-cleavable linker in plasma

To evaluate the in vivo stability and proteolytic cleavage of HER2-XPAT protein, the PK profiles of fully masked HER2-XPAT protein and HER2-XPAT-NoClvSite (a version of HER2-XPAT protein without the proteolytic cleavage sites) were compared following administration of high doses (HER2-XPAT protein 25 mg kg^−1^ or 42 mg kg^−1^ (*n* = 6); HER2-XPAT-NoClvSite 25 mg kg^−1^ or 42 mg kg^−1^ (*n* = 4)) as single i.v. infusions. Heparin-treated or K_2_EDTA-treated plasma samples were collected at different time points after infusion. The concentration of HER2-XPAT protein singly cleaved metabolites, HER2-XPAT(1x-C) and HER2-XPAT(1x-N), in NHP plasma was measured using a quantitative western blot method (as described above).

The stability of HER2-XPAT protein was evaluated in plasma from humans (four healthy volunteers, seven patients with lung cancer, three patients with breast cancer, one patient with colon cancer, eight patients with rheumatoid arthritis, six patients with multiple sclerosis, eight patients with systemic lupus erythematosus and five patients with inflammatory bowel disease; Supplementary Table 5) and NHPs (healthy monkeys and monkeys with drug-induced systemic inflammation). Fluorophore-labeled HER2-XPAT protein was spiked into plasma, incubated for 7 d at 37 °C and HER2-XPAT a protein and its cleavage products were quantified by SDS–PAGE using LI-COR technology. Products with a similar length to HER2-XPAT protein and each of its metabolites were quantified according to their size range on SDS–PAGE gels. The percentage of product is presented as relative to the total fluorescent signal of HER2-XPAT-derived species present in the plasma samples. Plasma samples from NHPs with systemic inflammation were collected from NHPs with drug-induced CRS from high doses of unmasked EGFR-TCE or unmasked HER2-TCE.

### Statistics and reproducibility

The number of replicates in each experiment are noted on the figures or in the figure legends. Sample sizes for animal experiments were selected based on the animal model used and the predicted variability of the parameters being evaluated. The method used for determining the sample size for the mouse tumor xenograft models was as described previously^40^.

For tumor xenograft studies, randomization was performed using a tumor volume-stratified randomization method. Each test within an in vitro experiment was conducted under identical test conditions and no randomization or blinding was applied. No animals or data points were excluded from data analyses for any of the experiments. Data distribution was assumed to be normal, but this was not formally tested.

Statistical analyses were performed using GraphPad Prism software v.9.5.0. Results were determined to be significant at *P* < 0.05. EC_50_ values were derived with a four-parameter logistic regression equation using GraphPad Prism software. Mixed-effects multiple comparison analyses followed by Tukey’s post hoc test were used to evaluate statistical differences between the treatment groups versus vehicle in the mouse in vivo studies. Unpaired *t*-tests were used to evaluate differences between XPAT protein cleavage products following incubation in healthy human and healthy cynomolgus plasma and plasma from humans and NHPs with inflammation.

### Reporting summary

Further information on research design is available in the Nature Portfolio Reporting Summary linked to this article.

### Supplementary information

Supplementary Information Supplementary Figs. 1–22 and Supplementary Tables 1–5.

Reporting Summary

### Source data

Source Data Fig. 2 Statistical source data.

Source Data Fig. 3 Statistical source data.

Source Data Fig. 4 Statistical source data.

Source Data Fig. 5 Statistical source data.

Source Data Fig. 6 Statistical source data.

Source Data Fig. 7 Statistical source data.

Source Data Extended Data Fig. 1 Statistical source data.

Source Data Extended Data Fig. 2 Statistical source data.

Source Data Extended Data Fig. 3 Statistical source data.

## Figures and Tables

**Extended Data Table. 1:**
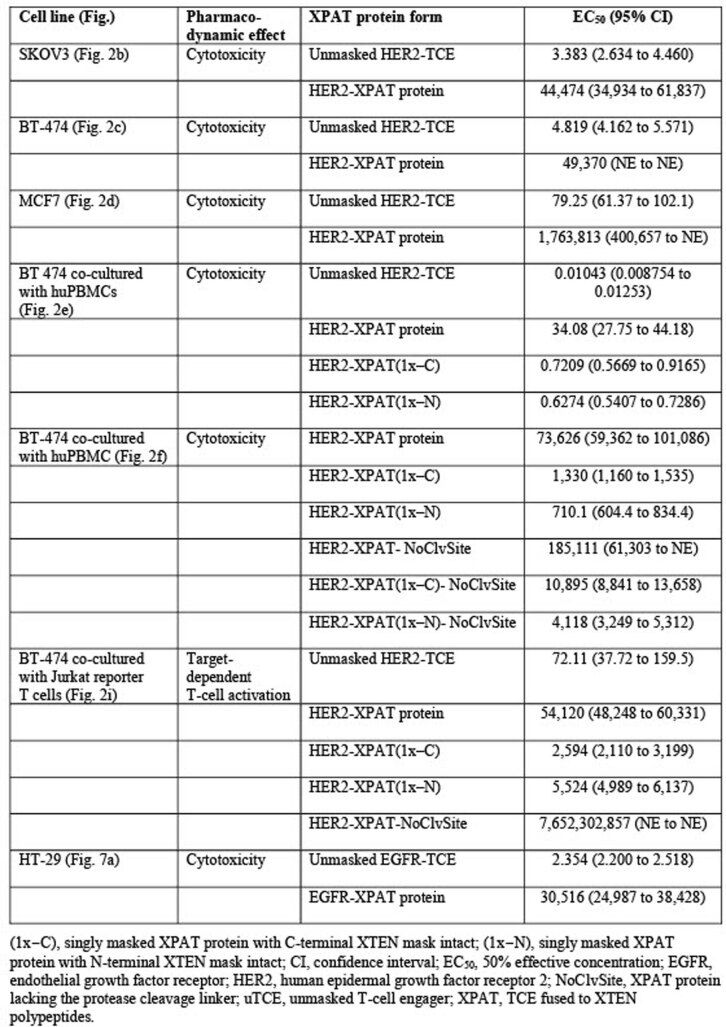
EC_50_ values from cell culture experiments with HER2-XPAT and EGFR-XPAT proteins

*In vitro* tumor cytotoxicity of HER2-XPAT protein and its metabolites was investigated using co-cultures of huPBMCs with HER2-expressing human tumor cell lines. The impact of the protease-cleavable linker on *in vitro* cytotoxicity was investigated in BT-474 cells co-cultured with huPBMCs. T-cell activation by HER2-XPAT protein and its proteolytically activated metabolites was characterized using Jurkat *NFAT*-luciferase reporter T cells co-cultured with BT-474 tumor cells. *In vitro* cytotoxicity of the EGFR-XPAT protein and EGFR-uTCE was evaluated using HT-29 (*BRAF*^mut^) cells. This table presents the EC_50_ values for these cytotoxicity and reporter T-cell activation assays. (1x–C), singly masked XPAT protein with C-terminal XTEN mask intact; (1x–N), singly masked XPAT protein with N-terminal XTEN mask intact; CI, confidence interval; EC50, 50% effective concentration; EGFR, endothelial growth factor receptor; HER2, human epidermal growth factor receptor 2; NoClvSite, XPAT protein lacking the protease cleavage linker; uTCE, unmasked T-cell engager; XPAT, TCE fused to XTEN polypeptides.

## Data Availability

Raw data for Figs. 2–7 and Extended Data Figs. 1–3 have been provided within the source data file. All other data supporting the findings of this study are available from the corresponding author on reasonable request. In response to reasonable requests, noncommercially available materials and experimental protocols that Amunix Pharmaceuticals has the right to provide will be made available to not-for-profit or academic requesters upon completion of a material transfer agreement. Requests can be made at bd@amunix.com. Source data are provided with this paper.

## References

[1] Goebeler M-E, Bargou RC (2020). T cell-engaging therapies—BiTEs and beyond. Nat. Rev. Clin. Oncol..

[2] Havel JJ, Chowell D, Chan TA (2019). The evolving landscape of biomarkers for checkpoint inhibitor immunotherapy. Nat. Rev. Cancer.

[3] Ribas A, Wolchok JD (2018). Cancer immunotherapy using checkpoint blockade. Science.

[4] Clynes RA, Desjarlais JR (2019). Redirected T cell cytotoxicity in cancer therapy. Ann. Rev. Med..

[5] Lowe KL (2019). Novel TCR-based biologics: mobilising T cells to warm ‘cold’ tumours. Cancer Treat. Rev..

[6] Ochoa de Olza M, Navarro Rodrigo B, Zimmermann S, Coukos G (2020). Turning up the heat on non-immunoreactive tumours: opportunities for clinical development. Lancet Oncol..

[7] Klinger M, Benjamin J, Kischel R, Stienen S, Zugmaier G (2016). Harnessing T cells to fight cancer with BiTE antibody constructs—past developments and future directions. Immunol. Rev..

[8] Vafa O, Trinklein ND (2020). Perspective: designing T-cell engagers with better therapeutic windows. Front. Oncol..

[9] Saber H, Del Valle P, Ricks TK, Leighton JK (2017). An FDA oncology analysis of CD3 bispecific constructs and first-in-human dose selection. Regul. Toxicol. Pharmacol..

[10] Kantarjian H (2017). Blinatumomab versus chemotherapy for advanced acute lymphoblastic leukemia. N. Engl. J. Med..

[11] Schellenberger V (2009). A recombinant polypeptide extends the in vivo half-life of peptides and proteins in a tunable manner. Nat. Biotechnol..

[12] Podust VN (2016). Extension of in vivo half-life of biologically active molecules by XTEN protein polymers. J. Control. Release.

[13] von Drygalski, A. et al. Efficacy, safety, and pharmacokinetics of once-weekly efanesoctocog alfa (BIVV001) prophylaxis in previously treated patients with severe hemophilia a: results from the phase 3 XTEND-1 study. https://abstracts.isth.org/abstract/efficacy-safety-and-pharmacokinetics-of-once-weekly-efanesoctocog-alfa-bivv001-prophylaxis-in-previously-treated-patients-with-severe-hemophilia-a-results-from-the-phase-3-xtend-1-study/ (ISTH, 2022).

[14] Yuen KCJ (2013). A long-acting human growth hormone with delayed clearance (VRS-317): results of a double-blind, placebo-controlled, single ascending dose study in growth hormone-deficient adults. J. Clin. Endocrinol. Metab..

[15] Florido R, Smith KL, Cuomo KK, Russell SD (2017). Cardiotoxicity from human epidermal growth factor receptor-2 (HER2) targeted therapies. J. Am. Heart Assoc..

[16] Alters SE (2012). GLP2-2G-XTEN: a pharmaceutical protein with improved serum half-life and efficacy in a rat Crohn’s disease model. PLoS ONE.

[17] Lee DW (2019). ASTCT consensus grading for cytokine release syndrome and neurologic toxicity associated with immune effector cells. Biol. Blood Marrow Transplant..

[18] Khadka RH, Sakemura R, Kenderian SS, Johnson AJ (2019). Management of cytokine release syndrome: an update on emerging antigen-specific T cell engaging immunotherapies. Immunotherapy..

[19] Kiewe P (2006). Phase I trial of the trifunctional anti-HER2 x anti-CD3 antibody ertumaxomab in metastatic breast cancer. Clin. Cancer Res..

[20] Wermke M (2018). Preliminary results from a phase I study of GBR 1302, a bispecific antibody T-cell engager, in HER2-positive cancers. Ann. Oncol..

[21] Podust VN (2013). Extension of in vivo half-life of biologically active peptides via chemical conjugation to XTEN protein polymer. Protein Eng. Des. Sel..

[22] Ding S (2014). Multivalent antiviral XTEN-peptide conjugates with long in vivo half-life and enhanced solubility. Bioconjug. Chem..

[23] Cleland JL (2012). A novel long-acting human growth hormone fusion protein (VRS-317): enhanced in vivo potency and half-life. J. Pharm. Sci..

[24] Cleland J (2012). Safety, pharmacokinetics, and pharmacodynamics of a single subcutaneous dose of VRS-859 in patients with type 2 diabetes. Diabetologia.

[25] Cleland JL, Silverman J, Schellenberger V (2009). An extended half-life exenatide construct for weekly administration in the treatment of diabetes mellitus. Diabetes..

[26] Chhabra ES (2020). BIVV001, a new class of factor VIII replacement for hemophilia A that is independent of von Willebrand factor in primates and mice. Blood..

[27] van der Flier A (2015). Prolonged half-life and improved recovery of recombinant factor IX-XTEN fusion proteins in hemophilia B mouse model. Blood..

[28] Miller BS (2017). Bright, 3-year safety and efficacy update of the VERTICAL & VISTA trials of somavaratan (VRS-317), a long-acting rhGH, in children with growth hormone deficiency (GHD). Endocr. Abstr..

[29] Zhou Y (2022). Immunogenicity assessment of bispecific antibody-based immunotherapy in oncology. J. Immunother. Cancer.

[30] Autio KA, Boni V, Humphrey RW, Naing A (2020). Probody therapeutics: an emerging class of therapies designed to enhance on-target effects with reduced off-tumor toxicity for use in immuno-oncology. Clin. Cancer Res..

[31] Johnson AM (2021). Phase I, first-in-human study of the probody therapeutic CX-2029 in adults with advanced solid tumor malignancies. Clin. Cancer Res..

[32] Kropshofer H (1992). Self-peptide released from class II HLA-DR1 exhibits a hydrophobic two-residue contact motif. J. Exp. Med..

[33] Andreatta M, Nielsen M (2012). Characterizing the binding motifs of 11 common human HLA-DP and HLA-DQ molecules using NNAlign. Immunology..

[34] Purbhoo MA, Irvine DJ, Huppa JB, Davis MM (2004). T cell killing does not require the formation of a stable mature immunological synapse. Nat. Immunol..

[35] Davis DM, Dustin ML (2004). What is the importance of the immunological synapse?. Trends Immunol..

[36] National Research Council of the National Academies. *Guide for the Care and Use of Laboratory Animals* 8th edn (National Academies Press, 2011).

[37] Eiger E, Pondé NF, de Azambuja E (2019). Pertuzumab in HER2-positive early breast cancer: current use and perspectives. Future Oncol..

[38] Blumberg RS (1990). Structure of the T-cell antigen receptor: evidence for two CD3 epsilon subunits in the T-cell receptor–CD3 complex. Proc. Natl Acad. Sci. USA.

[39] Willuda J (1999). High thermal stability is essential for tumor targeting of antibody fragments: engineering of a humanized anti-epithelial glycoprotein-2 (epithelial cell adhesion molecule) single-chain Fv fragment. Cancer Res..

[40] Charan J, Kantharia ND (2013). How to calculate sample size in animal studies?. J. Pharmacol. Pharmacother..

[41] Dudani JS, Warren AD, Bhatia SN (2018). Harnessing protease activity to improve cancer care. Ann. Rev. Cancer Biol..

[42] Jumper J (2021). Highly accurate protein structure prediction with AlphaFold. Nature.

[43] Breshears, M. A. & Confer A. W. in *Pathologic Basis of Veterinary Disease* (ed. Zachary, J. F.) 617–681 (Elsevier, 2017).

[44] Hocum Stone L (2021). Serum cytokine profiles in healthy nonhuman primates are blunted by sedation and demonstrate sexual dimorphism as detected by a validated multiplex immunoassay. Sci. Rep..

