# Supplementary material for: Precision-activated T-cell engagers targeting HER2 or EGFR and CD3 mitigate on-target, off-tumor toxicity for immunotherapy in solid tumors
Source: Nat Cancer. 2023 Mar 30;4(4):485–501. doi: 10.1038/s43018-023-00536-9 (PMC10132983; doi:10.1038/s43018-023-00536-9)
Supplement: Supplementary file 1 — Supplementary Figs. 1–22 and Supplementary Tables 1–5. [file 43018_2023_536_MOESM1_ESM.pdf]

nature cancer

Article https://doi.org/10.1038/s43018-023-00536-9

Precision-activated T-cell engagers targeting HER2 or EGFR and CD3 mitigate on-target, off-tumor toxicity for immunotherapy in solid tumors

In the format provided by the authors and unedited

SUPPLEMENTARY INFORMATION

CONTENTS

Supplementary Figures

Supplementary **Fig. 1.** Gating strategy for the immunophenotyping flow cytometry analysis (mouse studies).

Supplementary **Fig. 2.** Gating strategy for flow cytometry analyses (NHP studies).

Supplementary **Fig. 3.** SPR sensorgrams showing the binding of HER2-XPAT protein to human HER2.

Supplementary **Fig. 4.** SPR sensorgrams showing the binding of HER2-XPAT(1x-N) protein to human HER2.

Supplementary **Fig. 5.** SPR sensorgrams showing the binding of HER2-XPAT(1x-C) protein to human HER2.

Supplementary **Fig. 6.** SPR sensorgrams showing the binding of HER2-XPAT(uTCE) protein to human HER2.

Supplementary **Fig. 7.** SPR sensorgrams showing the binding of HER2-XPAT protein to human CD3ε.

Supplementary **Fig. 8.** SPR sensorgrams showing the binding of HER2-XPAT(1x-N) protein to human CD3ε.

Supplementary **Fig. 9.** SPR sensorgrams showing the binding of HER2-XPAT(1x-C) protein to human CD3ε.

Supplementary **Fig. 10.** SPR sensorgrams showing the binding of HER2-XPAT(uTCE) protein to human CD3ε.

Supplementary **Fig. 11.** SPR sensorgrams showing the binding of HER2-XPAT protein to cynomolgus HER2.

Supplementary **Fig. 12.** SPR sensorgrams showing the binding of HER2-XPAT(1x-N) protein to cynomolgus HER2.

Supplementary **Fig. 13.** SPR sensorgrams showing the binding of HER2-XPAT(1x-C) protein to cynomolgus HER2.

Supplementary **Fig. 14.** SPR sensorgrams showing the binding of HER2-XPAT(uTCE) protein to cynomolgus HER2.

Supplementary **Fig. 15.** SPR sensorgrams showing the binding of HER2-XPAT protein to cynomolgus CD3ε.

Supplementary **Fig. 16.** SPR sensorgrams showing the binding of HER2-XPAT(1x-N) protein to cynomolgus CD3ε.

Supplementary **Fig. 17.** SPR sensorgrams showing the binding of HER2-XPAT(1x-C) protein to cynomolgus CD3ε.

Supplementary **Fig. 18.** SPR sensorgrams showing the binding of HER2-XPAT(uTCE) protein to cynomolgus CD3ε

Supplementary **Fig. 19.** SPR sensorgrams showing the binding of EGFR-XPAT protein to human HER2.

Supplementary **Fig. 20.** SPR sensorgrams showing the binding of EGFR-XPAT protein to cynomolgus HER2.

Supplementary **Fig. 21.** SPR sensorgrams showing the binding of EGFR-XPAT protein to human CD3ε.

Supplementary **Fig. 22.** SPR sensorgrams showing the binding of EGFR-XPAT protein to cynomolgus CD3ε.

Supplementary Tables

Supplementary **Table 1.** Summary of nomenclature for XPAT® molecules evaluated in the nonclinical studies reported here.

Supplementary **Table 2.** Amino acid sequences of the HER2-XPAT protein, HER2-XPAT-NoClvSite, EGFR-XPAT protein, and EpCAM-XPAT protein.

Supplementary **Table 3.** Tumor types used for patient-derived cancer xenograft models in immunodeficient mice.

Supplementary **Table 4.** Antibodies and fluorochromes used in the flow cytometry analyses (mouse studies).

Supplementary **Table 5.** List of human samples used for the analysis of HER2-XPAT protein plasma stability.

SUPPLEMENTARY FIGURES

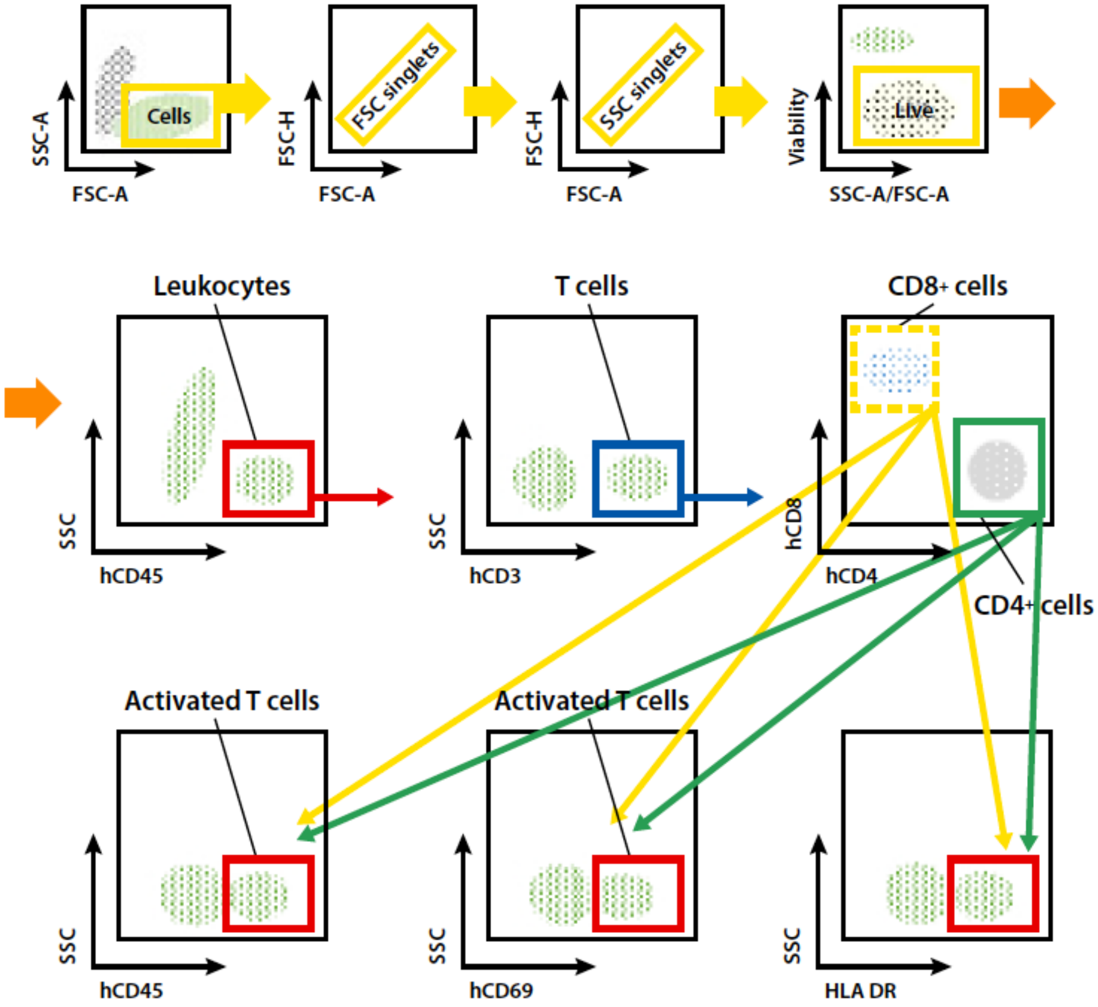

Supplementary Fig. 1. Gating strategy for the immunophenotyping flow cytometry analysis (mouse studies).

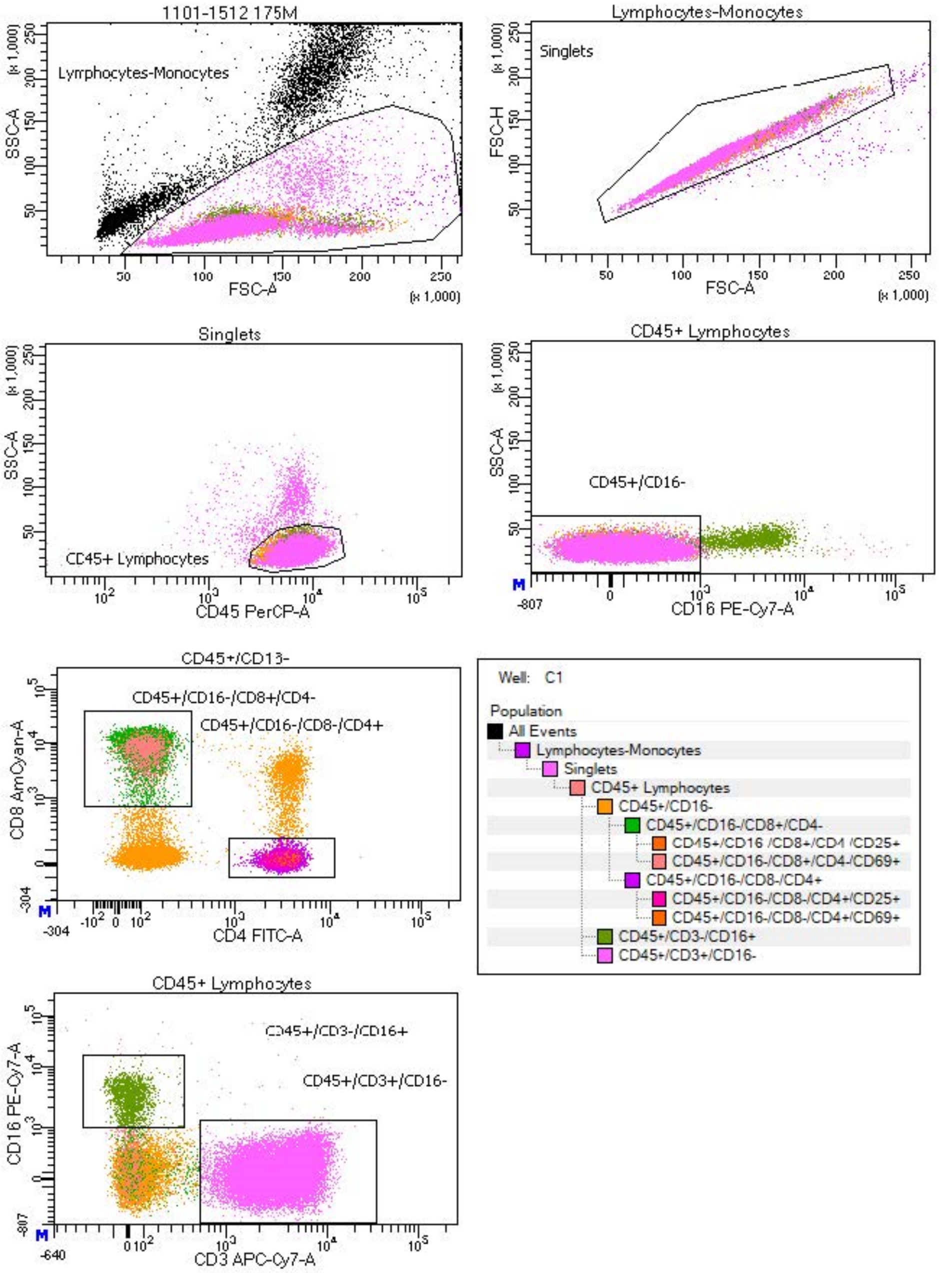

Supplementary Fig. 2. **Gating strategy for flow cytometry analyses (NHP toxicokinetic studies).**

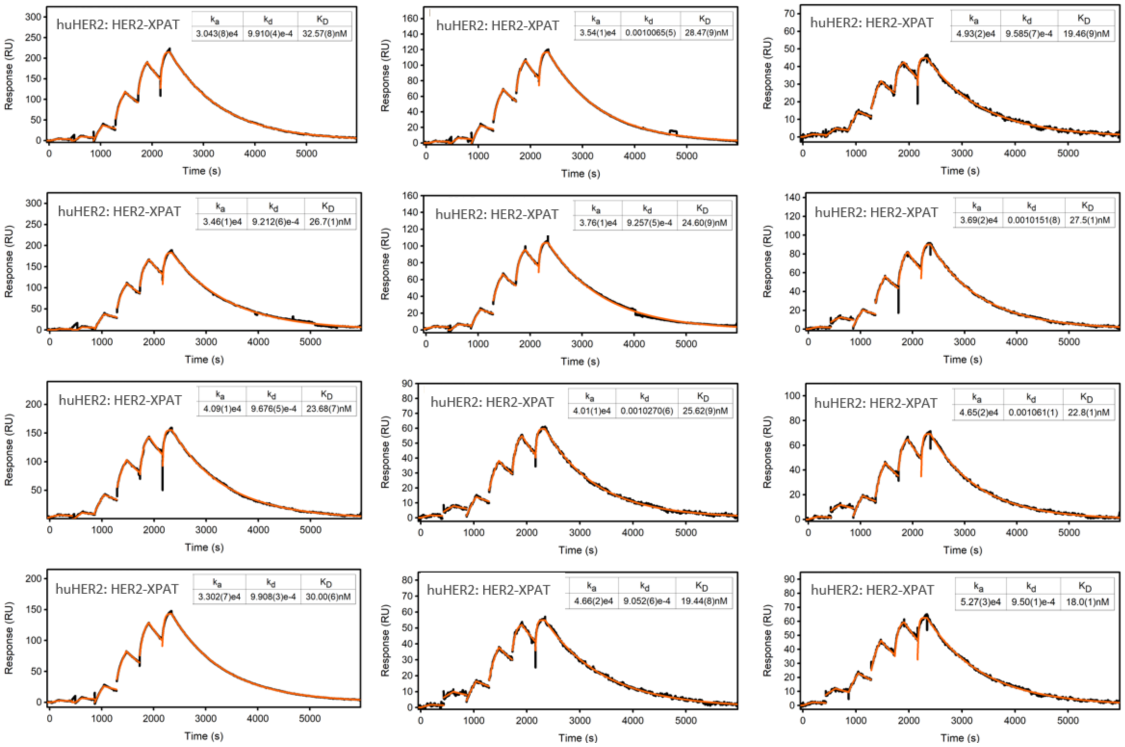

Supplementary Fig. 3. SPR sensorgrams showing the binding of HER2-XPAT protein to human HER2.

HER2, human epidermal growth factor receptor 2; hu, human; K_a, association constant; K_d, dissociation constant; K_D, equilibrium dissociation constant; s, seconds; SPR, surface plasmon resonance; TCE, T-cell engager; XPAT protein, TCE fused to XTEN polypeptides.

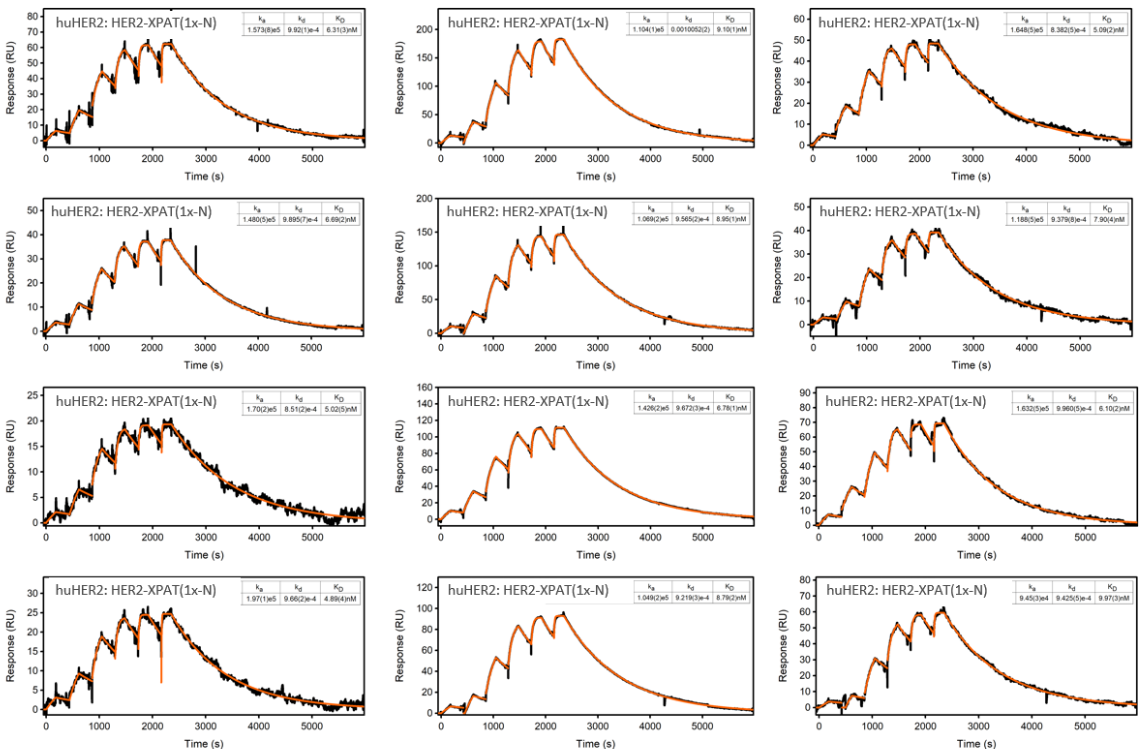

Supplementary Fig. 4. SPR sensorgrams showing the binding of HER2-XPAT(1x-N) protein to human HER2.

HER2, human epidermal growth factor receptor 2; hu, human; K_a, association constant; K_d, dissociation constant; K_D, equilibrium dissociation constant; s, seconds; SPR, surface plasmon resonance; TCE, T-cell engager; XPAT protein, TCE fused to XTEN polypeptides.

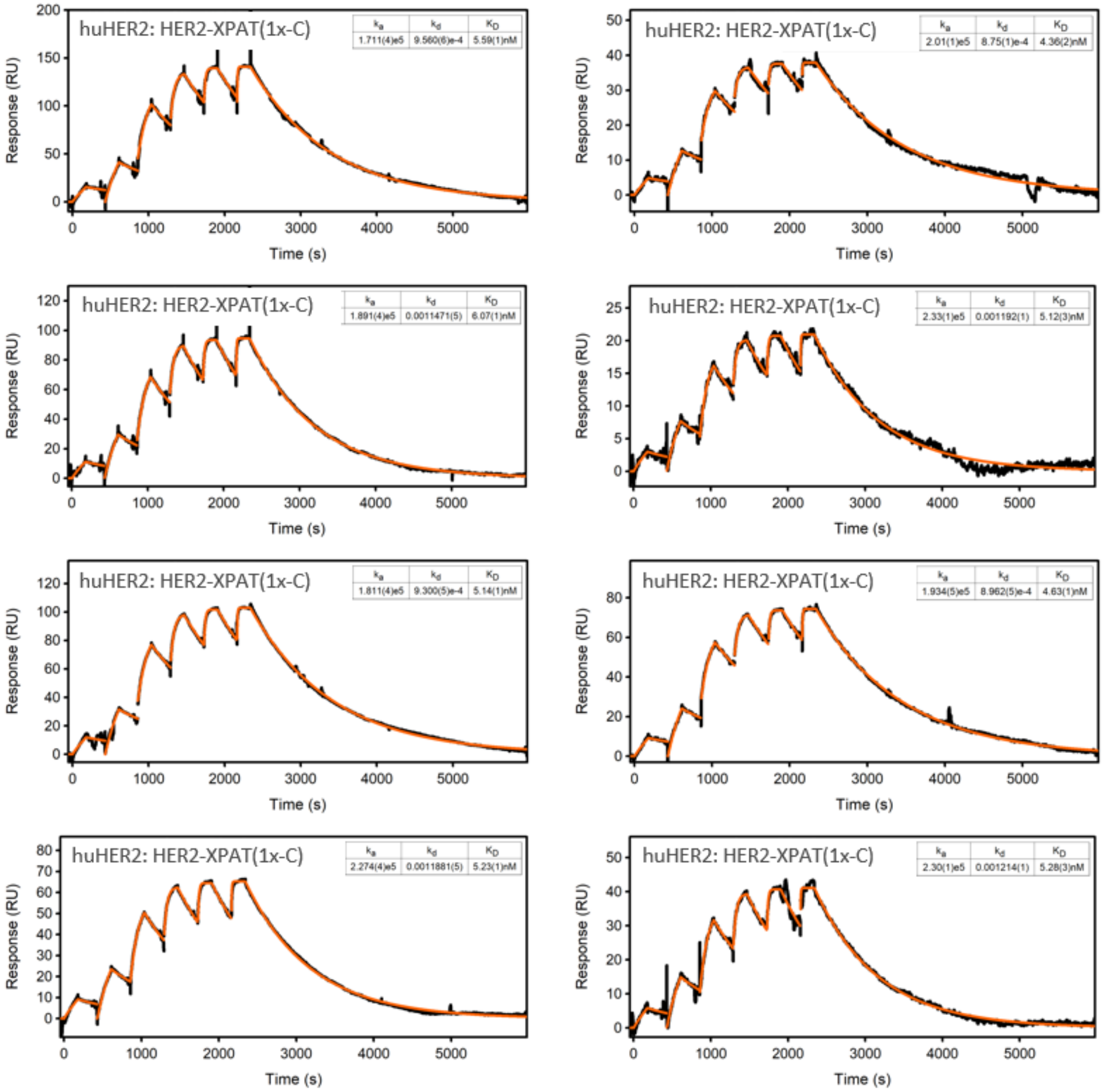

Supplementary Fig. 5. SPR sensorgrams showing the binding of HER2-XPAT(1x-C) protein to human HER2.

HER2, human epidermal growth factor receptor 2; hu, human; K_a, association constant; K_d, dissociation constant; K_D, equilibrium dissociation constant; s, seconds; SPR, surface plasmon resonance; TCE, T-cell engager; XPAT, TCE fused to XTEN polypeptides.

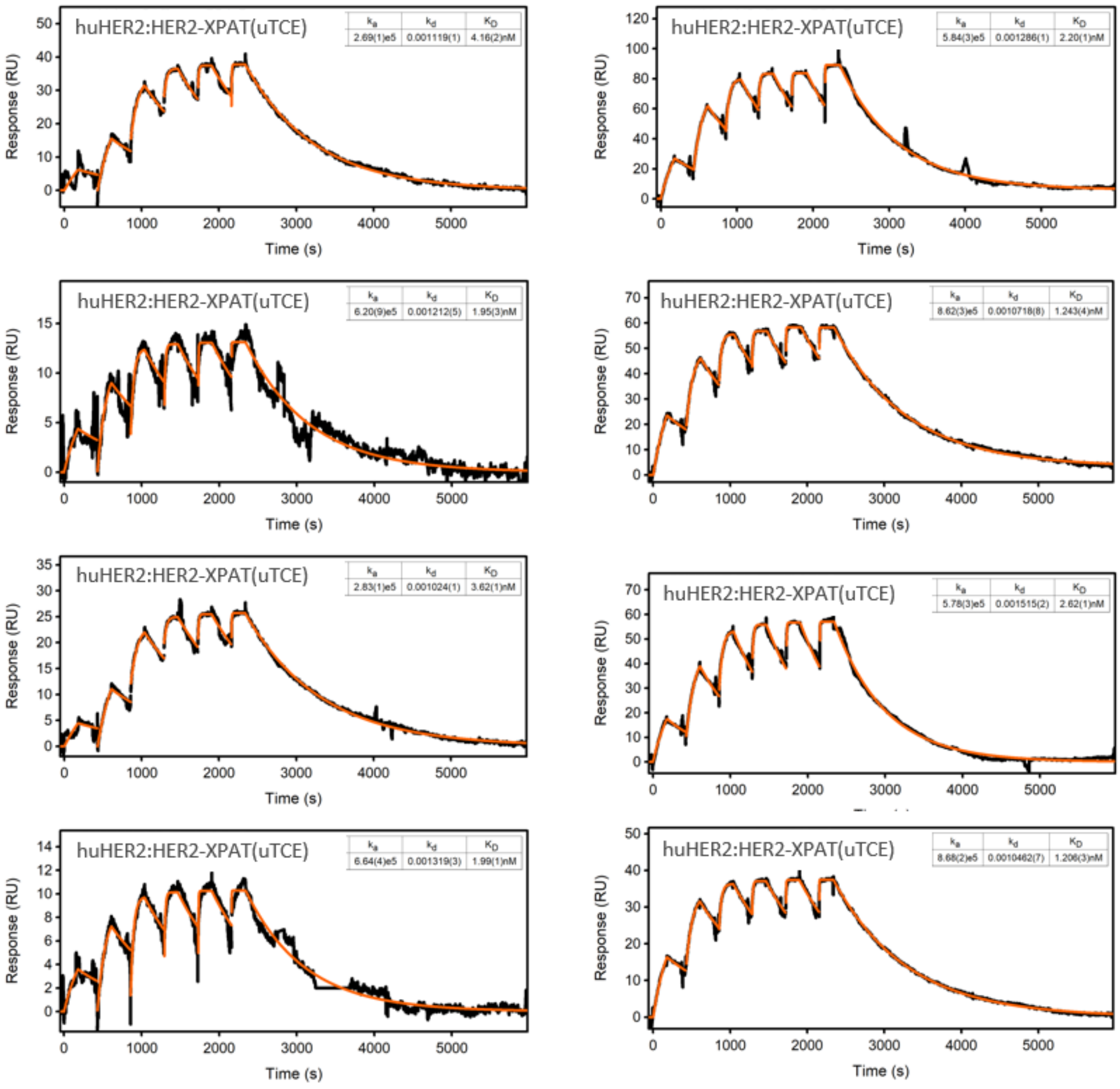

Supplementary Fig. 6. SPR sensorgrams showing the binding of HER2-XPAT(uTCE) protein to human HER2.

HER2, human epidermal growth factor receptor 2; hu, human; K_a, association constant; K_d, dissociation constant; K_D, equilibrium dissociation constant; s, seconds; SPR, surface plasmon resonance; TCE, T-cell engager; uTCE, unmasked T-cell engager; XPAT protein, TCE fused to XTEN polypeptides.

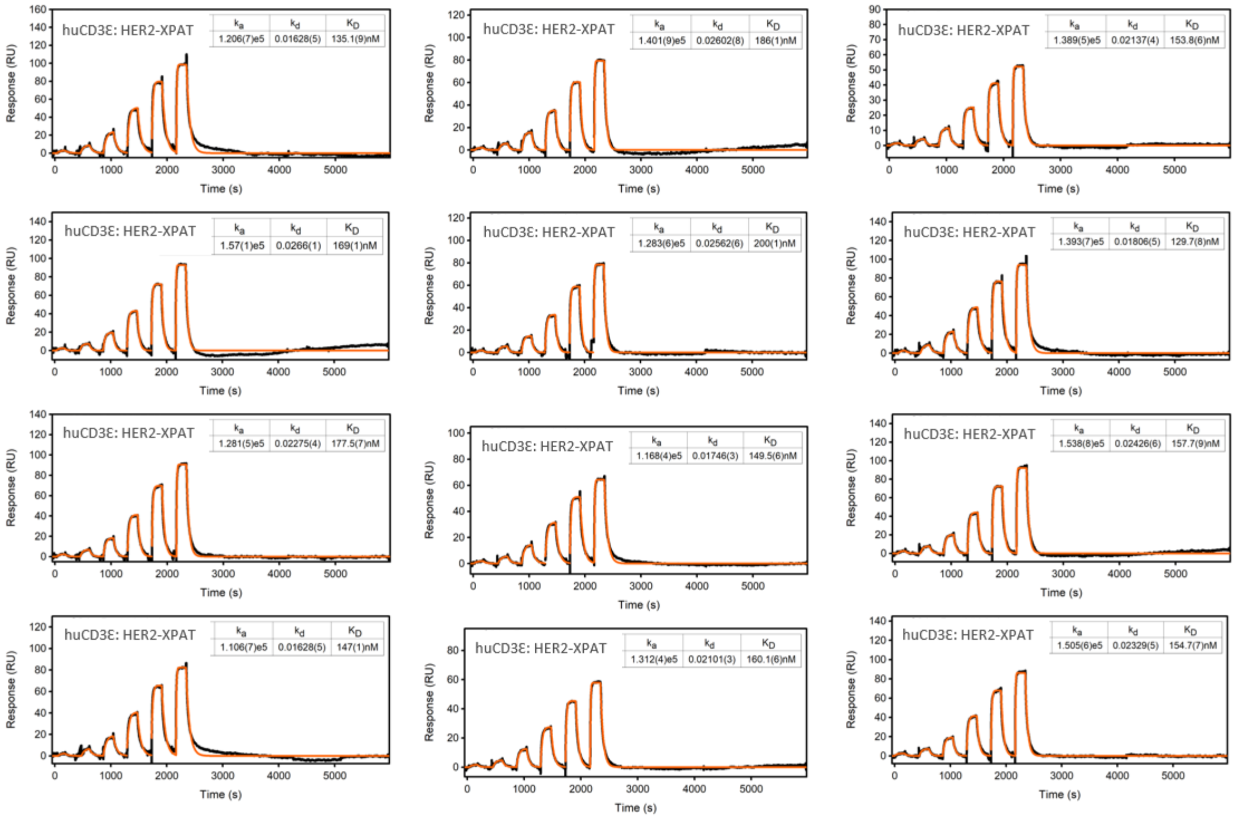

Supplementary Fig. 7. SPR sensorgrams showing the binding of HER2-XPAT protein to human CD3ε.

HER2, human epidermal growth factor receptor 2; hu, human; K_a, association constant; K_d, dissociation constant; K_D, equilibrium dissociation constant; s, seconds; SPR, surface plasmon resonance; TCE, T-cell engager; XPAT, TCE fused to XTEN polypeptides.

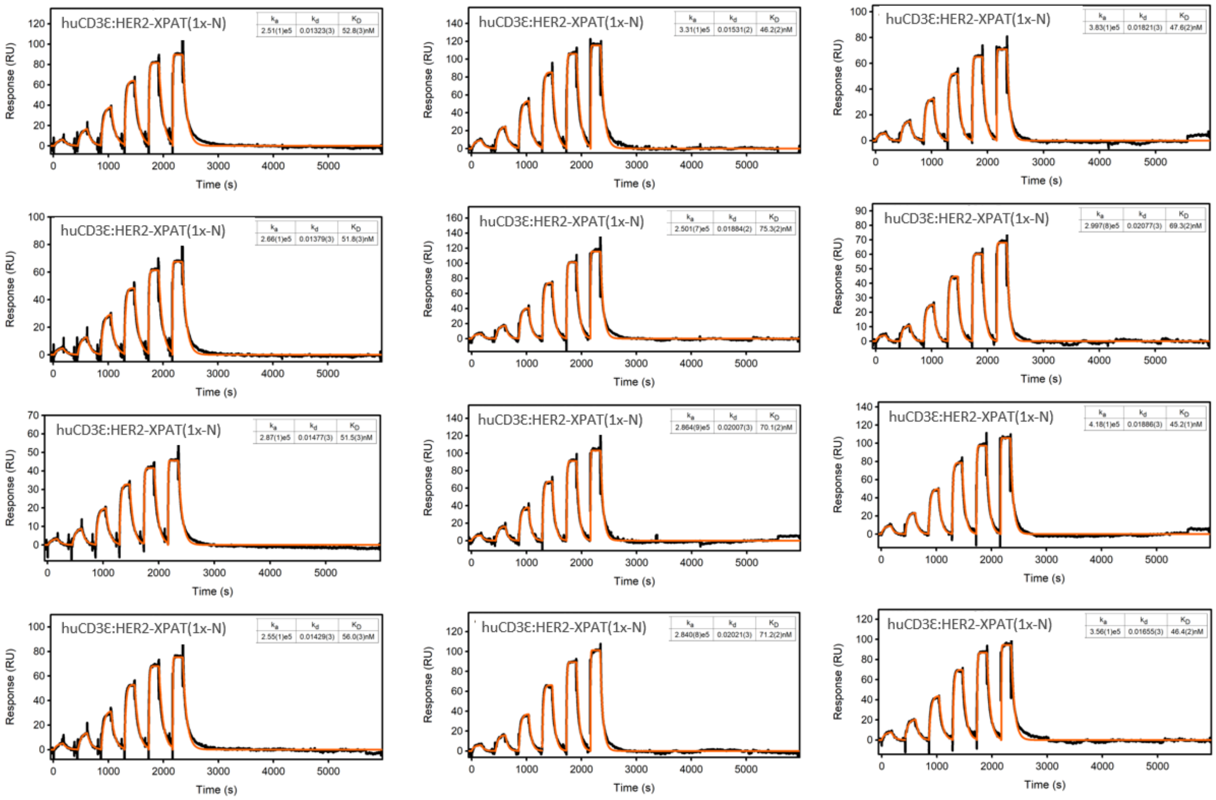

Supplementary Fig. 8. SPR sensorgrams showing the binding of HER2-XPAT(1x-N) protein to human CD3ε.

HER2, human epidermal growth factor receptor 2; hu, human; K_a, association constant; K_d, dissociation constant; K_D, equilibrium dissociation constant; s, seconds; SPR, surface plasmon resonance; TCE, T-cell engager; XPAT protein, TCE fused to XTEN polypeptides.

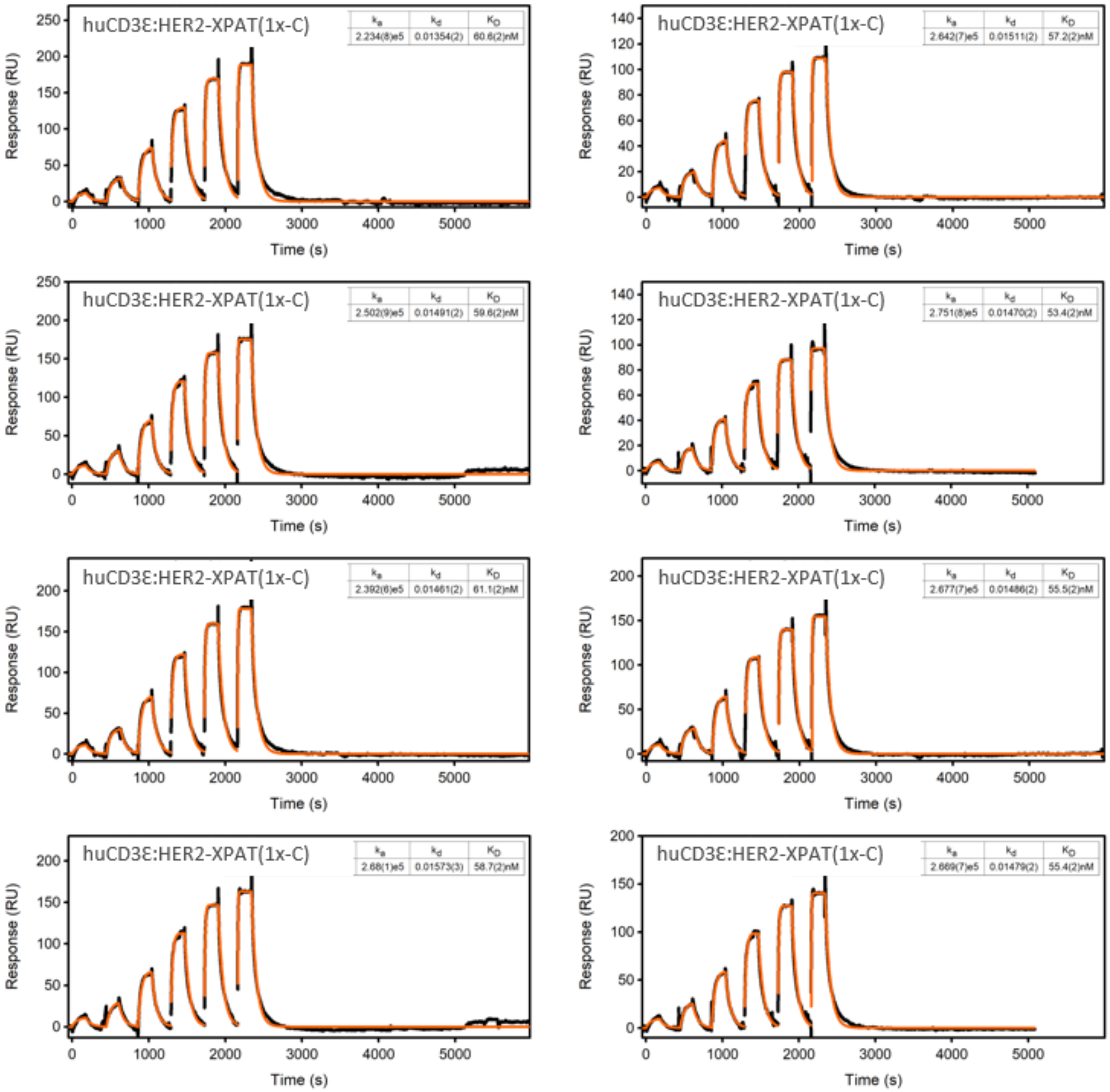

Supplementary Fig. 9. SPR sensorgrams showing the binding of HER2-XPAT(1x-C) protein to human CD3ε.

HER2, human epidermal growth factor receptor 2; hu, human; K_a, association constant; K_d, dissociation constant; K_D, equilibrium dissociation constant; s, seconds; SPR, surface plasmon resonance; TCE, T-cell engager; XPAT protein, TCE fused to XTEN polypeptides.

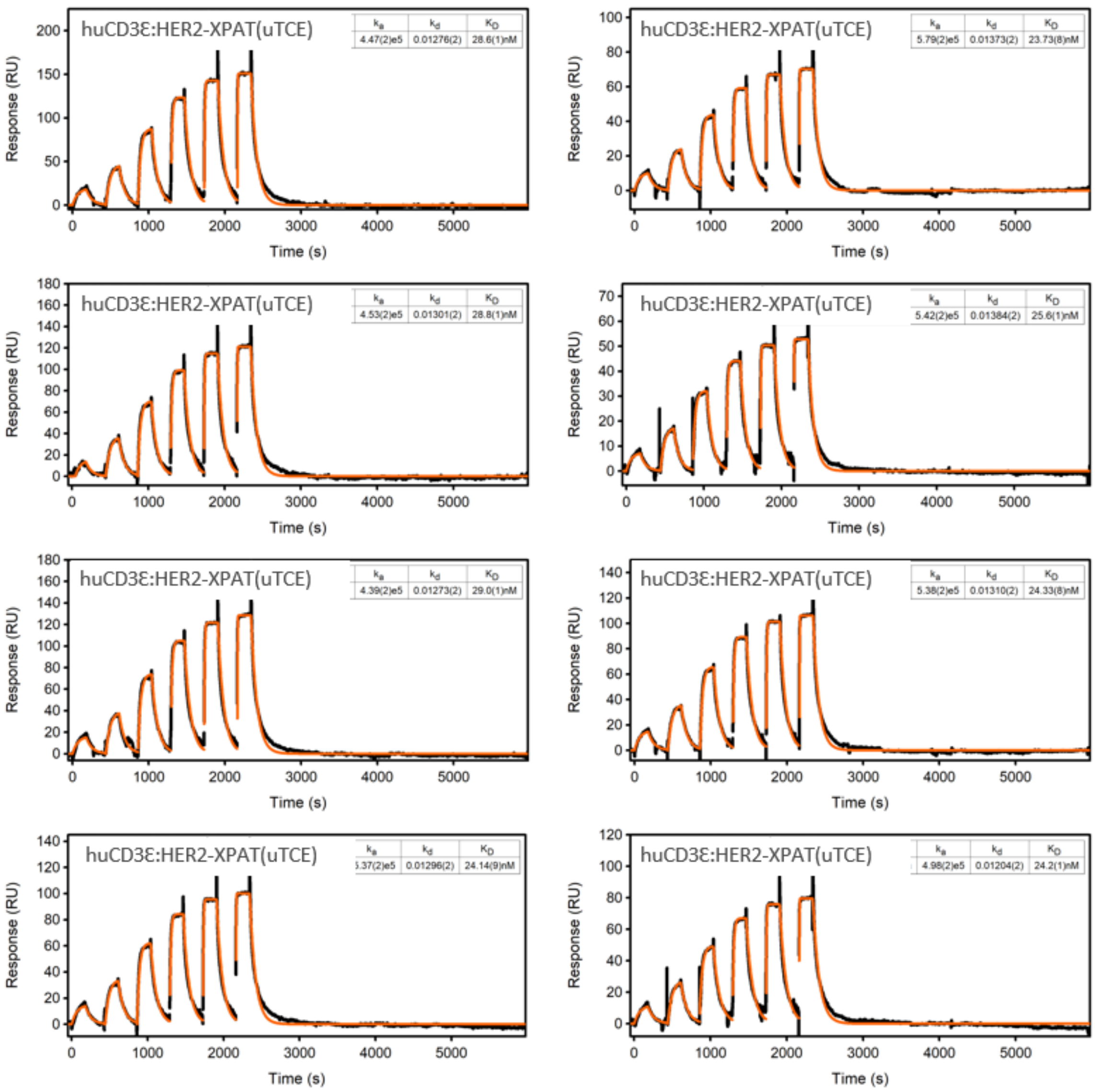

Supplementary Fig. 10. SPR sensorgrams showing the binding of HER2-XPAT(uTCE) protein to human CD3ε.

HER2, human epidermal growth factor receptor 2; hu, human; K_a, association constant; K_d, dissociation constant; K_D, equilibrium dissociation constant; s, seconds; SPR, surface plasmon resonance; TCE, T-cell engager; uTCE, unmasked T-cell engager; XPAT protein, TCE fused to XTEN polypeptides.

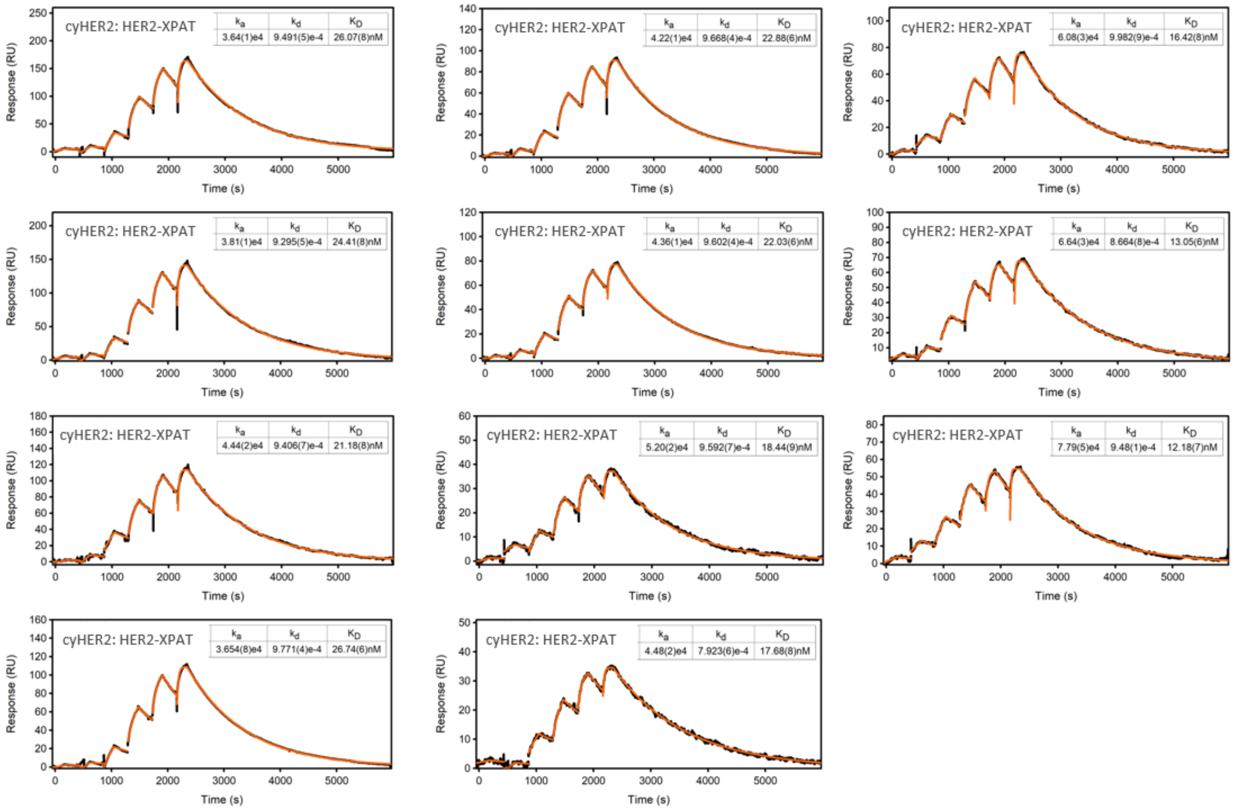

Supplementary Fig. 11. SPR sensorgrams showing the binding of HER2-XPAT protein to cynomolgus HER2.

cy, cynomolgus; HER2, human epidermal growth factor receptor 2; K_a, association constant; K_d, dissociation constant; K_D, equilibrium dissociation constant; s, seconds; SPR, surface plasmon resonance; TCE, T-cell engager; XPAT protein, TCE fused to XTEN polypeptides.

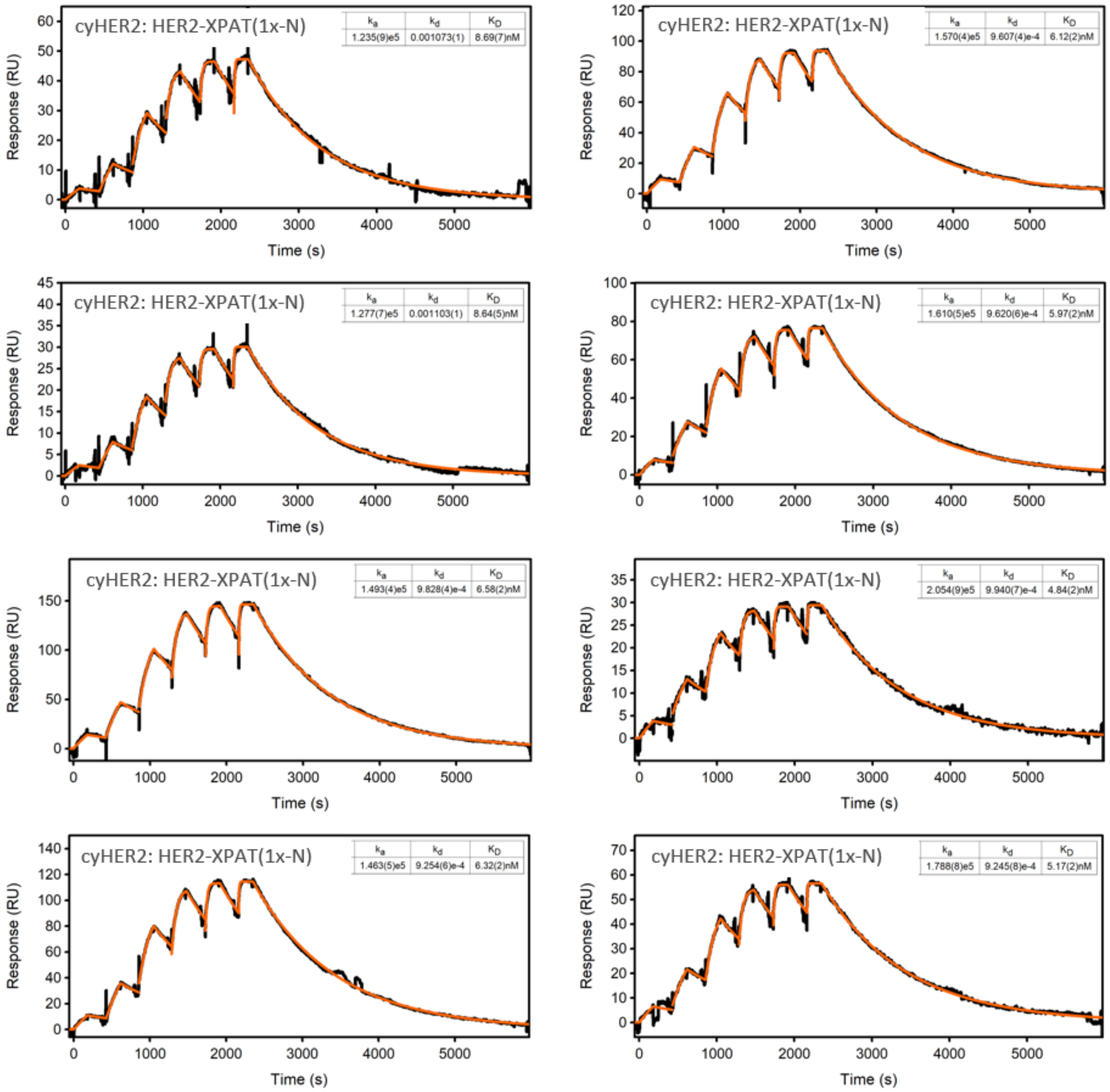

Supplementary Fig. 12. SPR sensorgrams showing the binding of HER2-XPAT(1x-N) protein to cynomolgus HER2.

cy, cynomolgus; HER2, human epidermal growth factor receptor 2; K_a, association constant; K_d, dissociation constant; K_D, equilibrium dissociation constant; s, seconds; SPR, surface plasmon resonance; TCE, T-cell engager; XPAT protein, TCE fused to XTEN polypeptides.

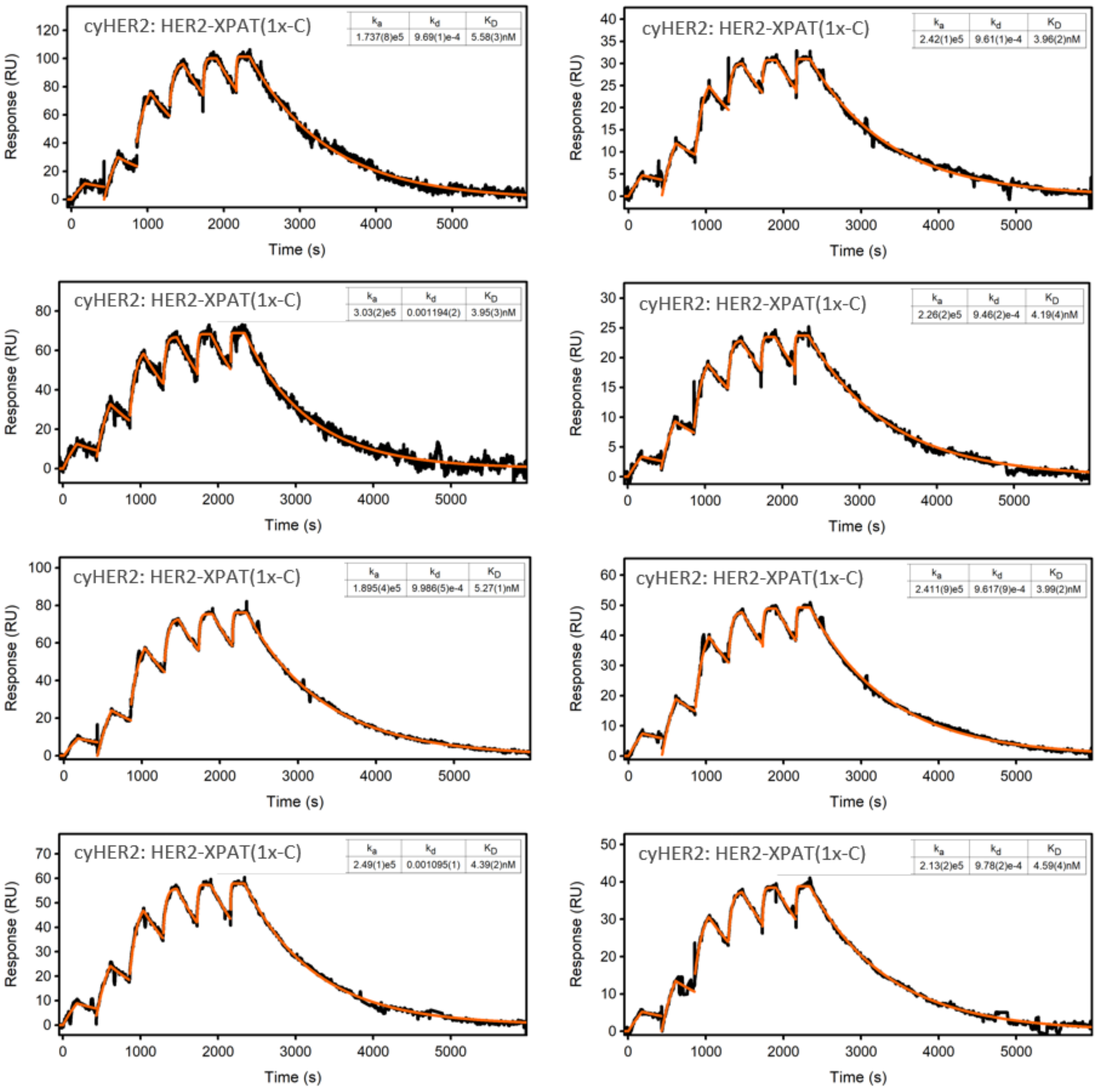

Supplementary Fig. 13. SPR sensorgrams showing the binding of HER2-XPAT(1x-C) protein to cynomolgus HER2.

cy, cynomolgus; HER2, human epidermal growth factor receptor 2; K_a, association constant; K_d, dissociation constant; K_D, equilibrium dissociation constant; s, seconds; SPR, surface plasmon resonance; TCE, T-cell engager; XPAT protein, TCE fused to XTEN polypeptides.

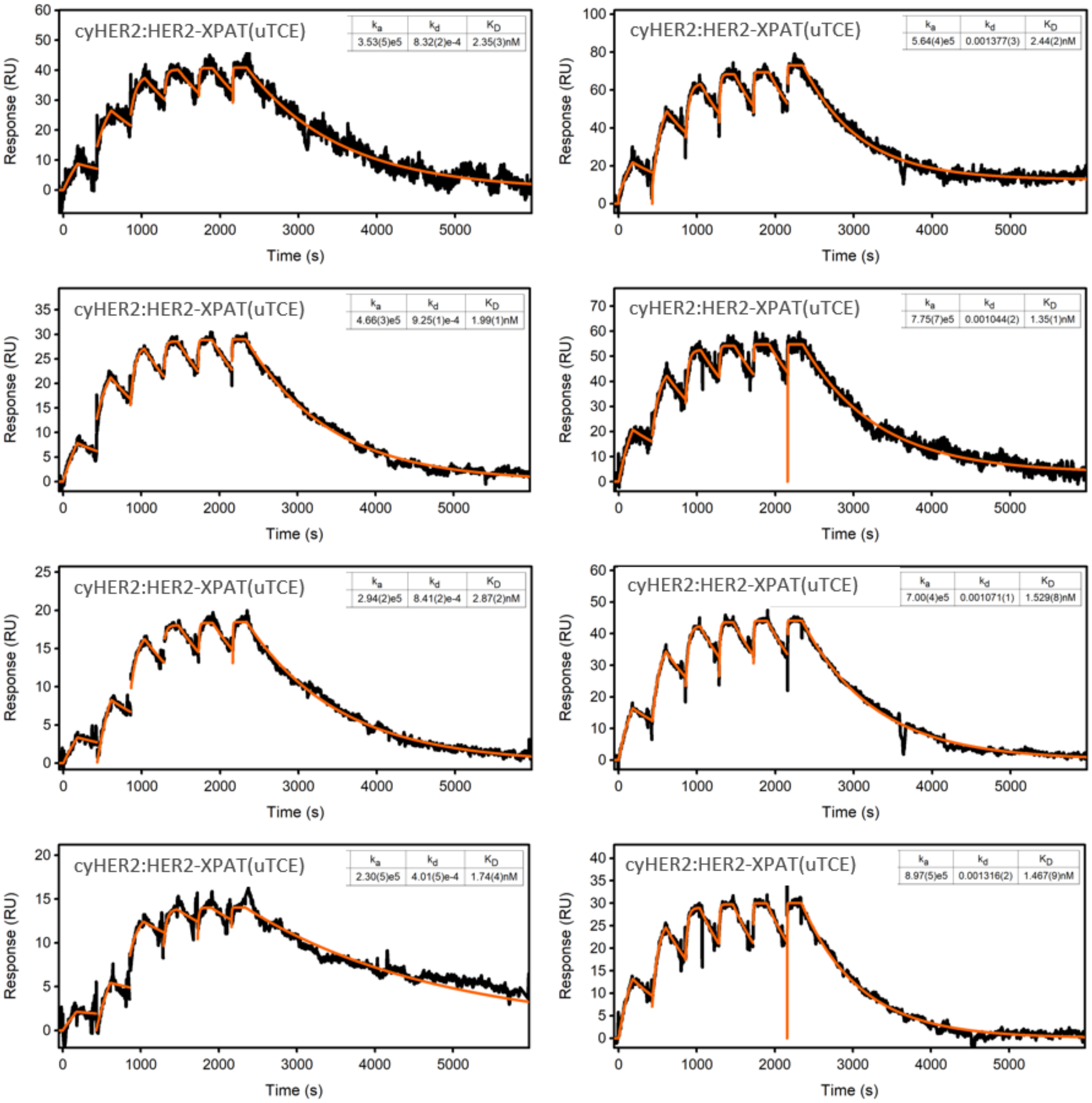

Supplementary Fig. 14. SPR sensorgrams showing the binding of HER2-XPAT(uTCE) protein to cynomolgus HER2.

cy, cynomolgus; HER2, human epidermal growth factor receptor 2; K_a, association constant; K_d, dissociation constant; K_D, equilibrium dissociation constant; s, seconds; SPR, surface plasmon resonance; TCE, T-cell engager; uTCE, unmasked T-cell engager; XPAT protein, TCE fused to XTEN polypeptides.

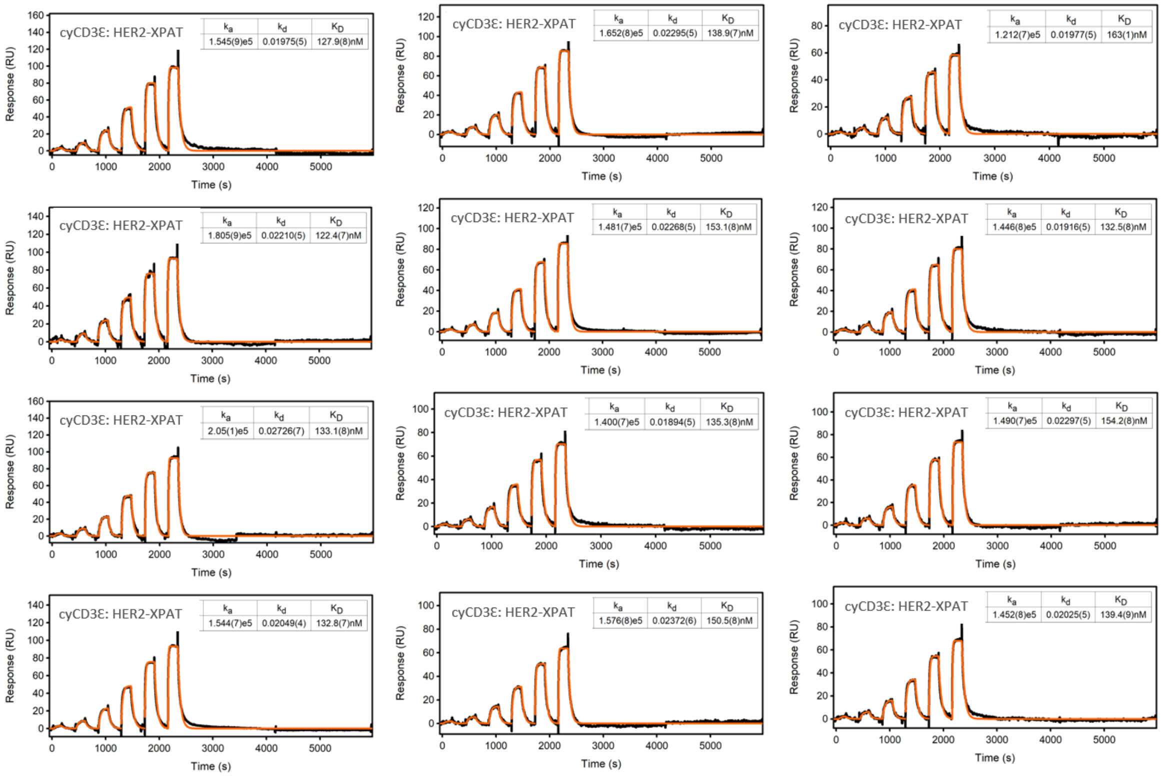

Supplementary Fig. 15. SPR sensorgrams showing the binding of HER2-XPAT protein to cynomolgus CD3ε.

cy, cynomolgus; HER2, human epidermal growth factor receptor 2; K_a, association constant; K_d, dissociation constant; K_D, equilibrium dissociation constant; s, seconds; SPR, surface plasmon resonance; TCE, T-cell engager; XPAT protein, TCE fused to XTEN polypeptides.

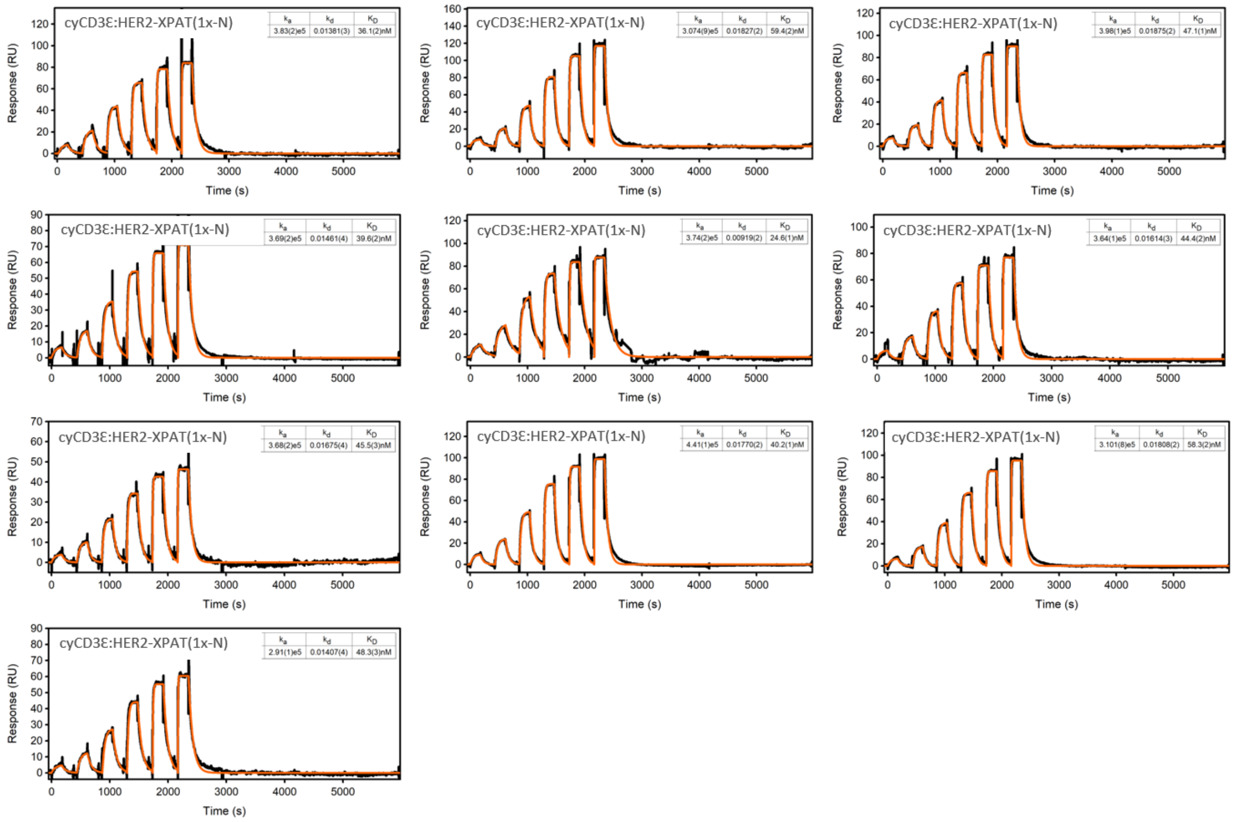

Supplementary Fig. 16. SPR sensorgrams showing the binding of HER2-XPAT(1x-N) protein to cynomolgus CD3ε.

cy, cynomolgus; HER2, human epidermal growth factor receptor 2; K_a, association constant; K_d, dissociation constant; K_D, equilibrium dissociation constant; s, seconds; SPR, surface plasmon resonance; TCE, T-cell engager; XPAT protein, TCE fused to XTEN polypeptides.

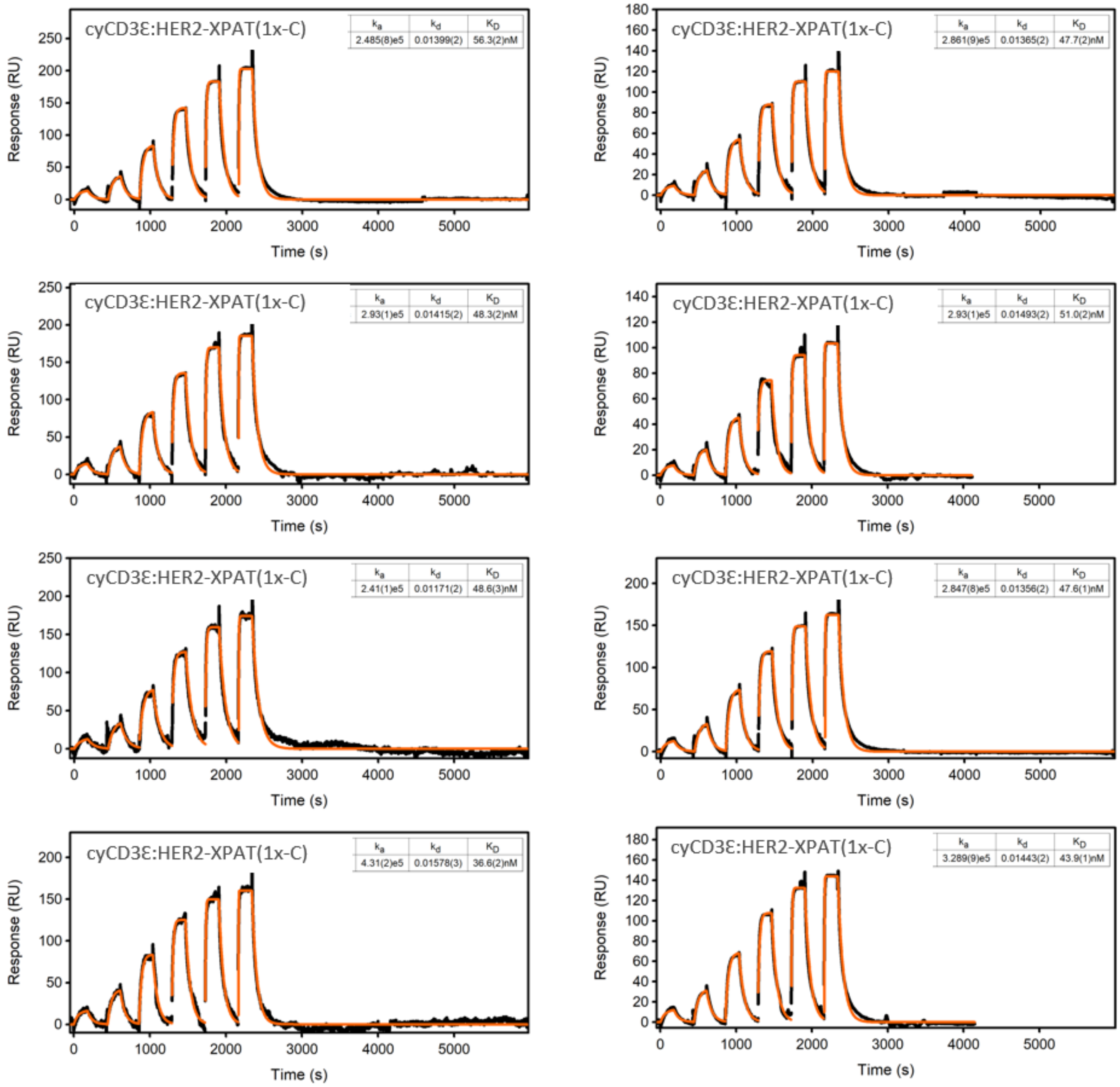

Supplementary Fig. 17. SPR sensorgrams showing the binding of HER2-XPAT(1x-C) protein to cynomolgus CD3ε.

cy, cynomolgus; HER2, human epidermal growth factor receptor 2; K_a, association constant; K_d, dissociation constant; K_D, equilibrium dissociation constant; s, seconds; SPR, surface plasmon resonance; TCE, T-cell engager; XPAT protein, TCE fused to XTEN polypeptides.

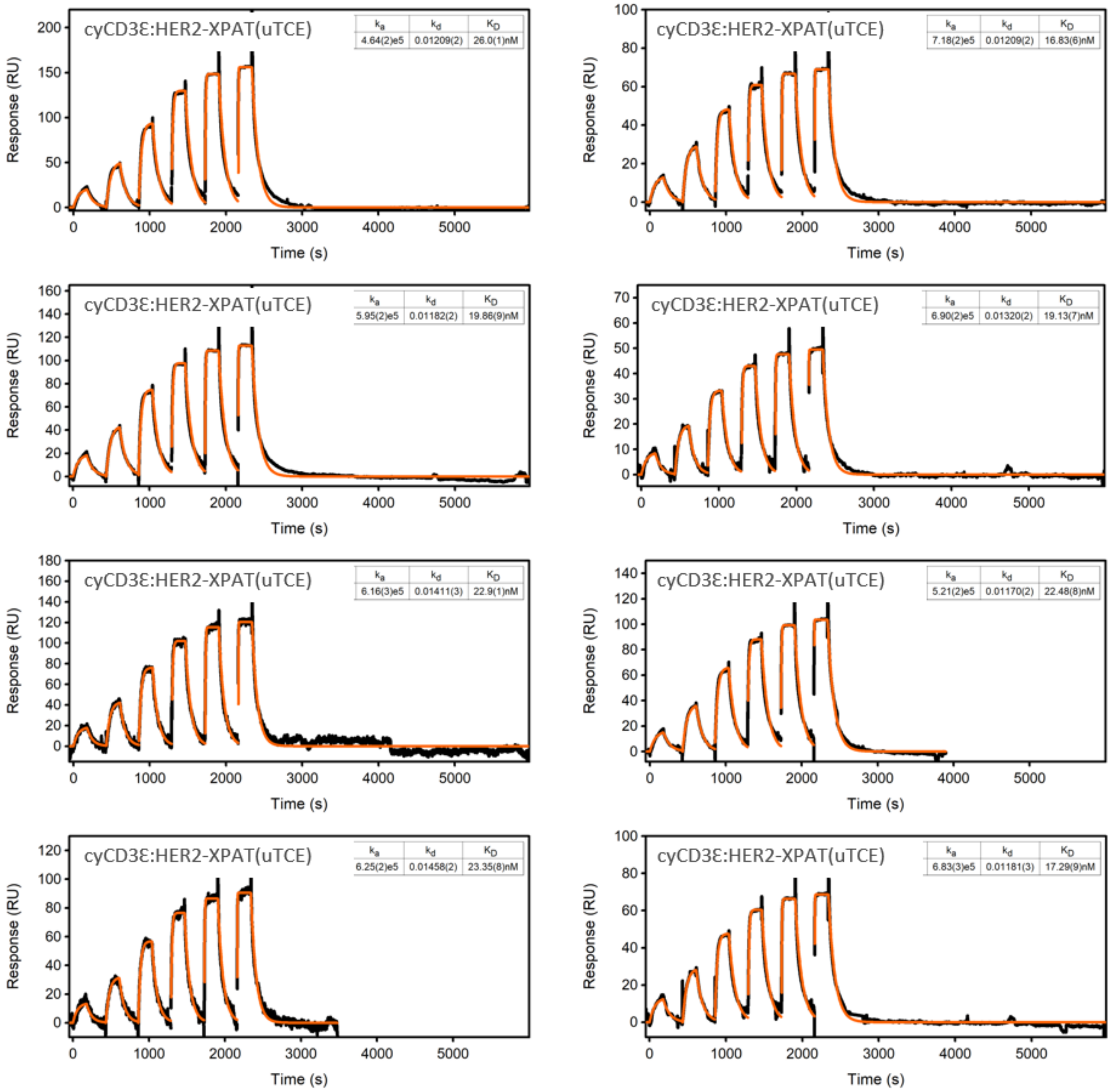

Supplementary Fig. 18. SPR sensorgrams showing the binding of HER2-XPAT(uTCE) protein to cynomolgus CD3ε.

cy, cynomolgus; HER2, human epidermal growth factor receptor 2; K_a, association constant; K_d, dissociation constant; K_D, equilibrium dissociation constant; s, seconds; SPR, surface plasmon resonance; TCE, T-cell engager; uTCE, unmasked T-cell engager; XPAT protein, TCE fused to XTEN polypeptides.

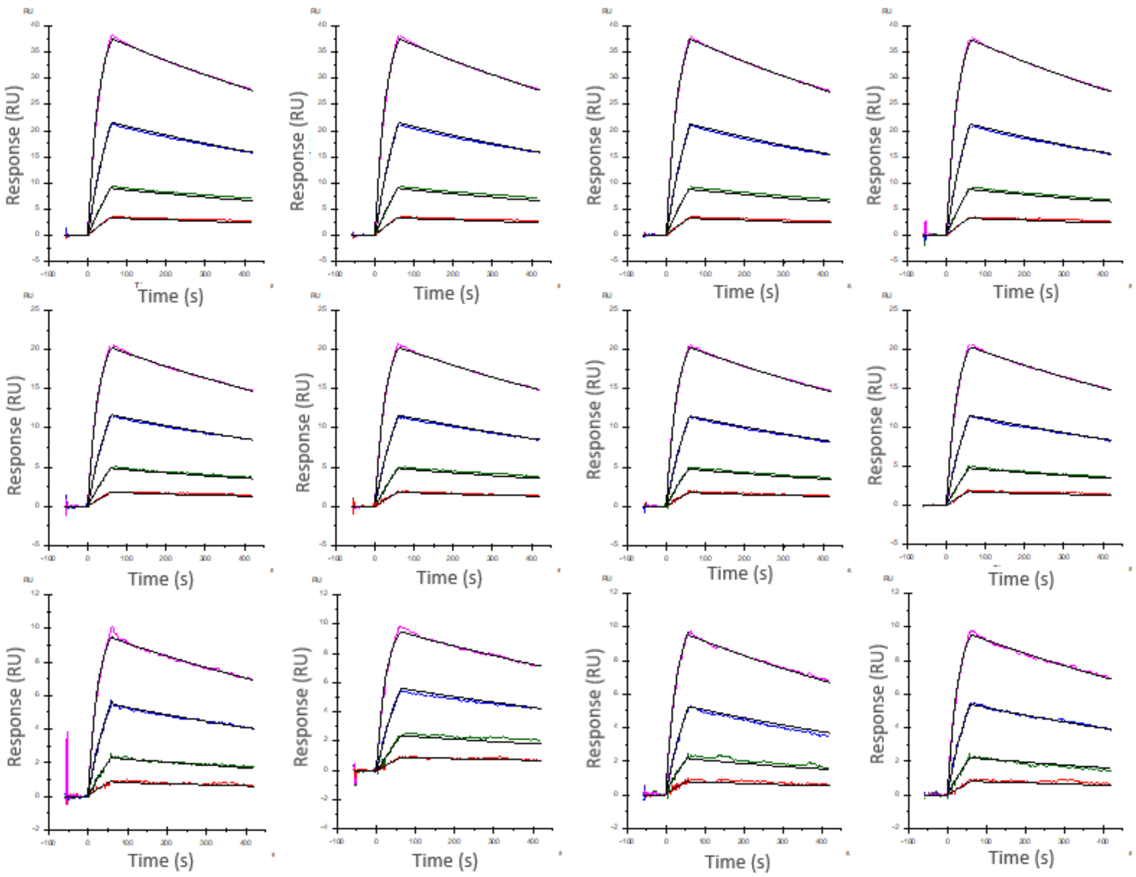

Supplementary Fig. 19. SPR sensorgrams showing the binding of EGFR-XPAT protein to human EGFR.

EGFR, epidermal growth factor receptor; s, seconds; SPR, surface plasmon resonance; XPAT protein, TCE fused to XTEN polypeptides.

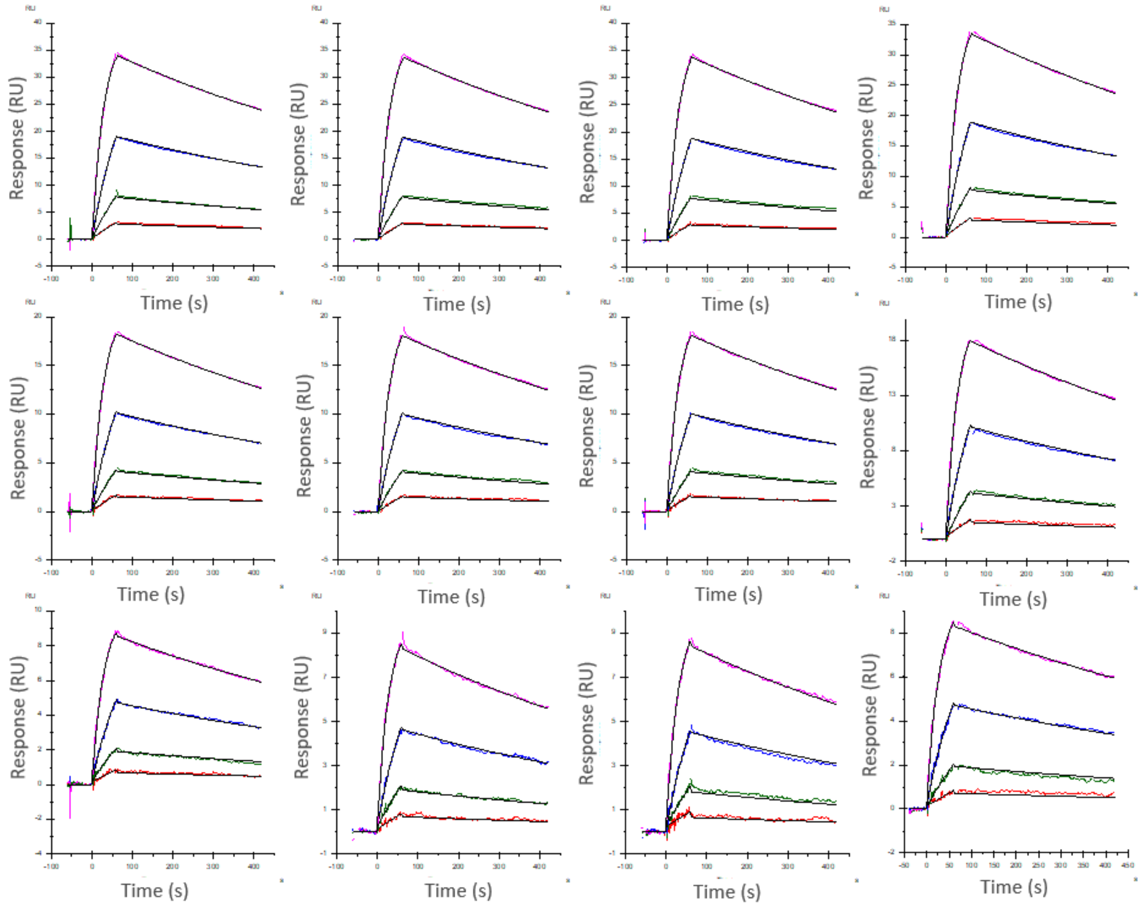

Supplementary Fig. 20. SPR sensorgrams showing the binding of EGFR-XPAT protein to cynomolgus EGFR.

cy, cynomolgus; EGFR, epidermal growth factor receptor; s, seconds; SPR, surface plasmon resonance; XPAT protein, TCE fused to XTEN polypeptides.

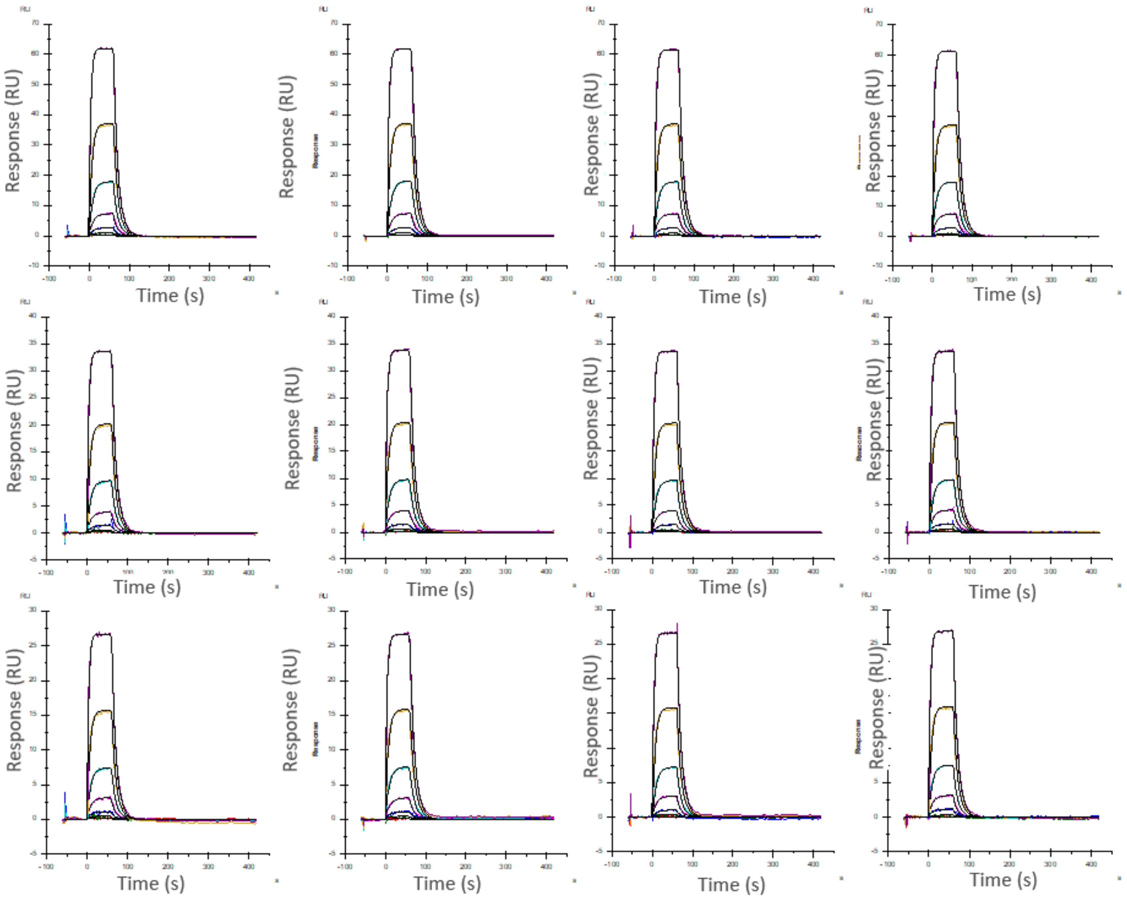

Supplementary Fig. 21. SPR sensorgrams showing the binding of EGFR-XPAT protein to human CD3ε.

EGFR, epidermal growth factor receptor; s, seconds; SPR, surface plasmon resonance; XPAT, TCE fused to XTEN polypeptides.

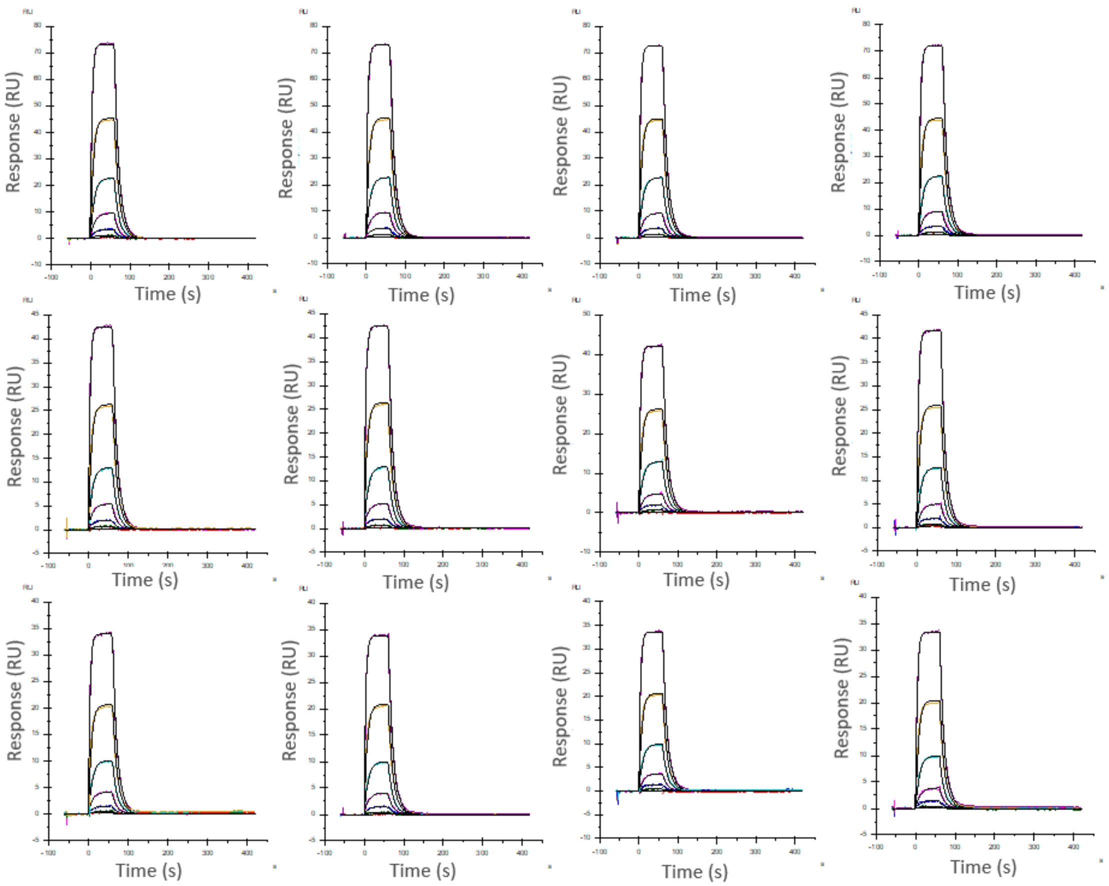

Supplementary Fig. 22. SPR sensorgrams showing the binding of EGFR-XPAT protein to cynomolgus CD3ε.

cy, cynomolgus; EGFR, epidermal growth factor receptor; s, seconds; SPR, surface plasmon resonance; XPAT protein, TCE fused to XTEN polypeptides.

SUPPLEMENTARY TABLES

Supplementary Table 1. Summary of nomenclature for XPAT molecules evaluated in the nonclinical studies reported here.

**Name**	**Description**
**HER2-XPAT protein**	
HER2-XPAT prototype	Prototype engineered with the XTEN polypeptide masks on the N- and C-termini
HER2-XPAT protein	Clinical candidate engineered with optimized XTEN polypeptide masks on the N- and C-termini
**EGFR-XPAT protein**	
EGFR-XPAT prototype	Prototype engineered with XTEN polypeptide masks on the N- and C-termini
**XPAT protein metabolites**	
XPAT(1x–C)	Singly masked XPAT protein: C-terminal XTEN mask remains intact<br>N-terminal XTEN mask cleaved off (anti-TAA scFv unmasked)
XPAT(1x–N)	Singly masked XPAT protein: N-terminal XTEN mask remains intact<br>C-terminal XTEN mask cleaved off (anti-CD3 scFv unmasked)
Unmasked XPAT (uTCE)	Fully active TCE, with both XTEN polypeptide masks cleaved off
**Reference compounds**	
XPAT-NoClvSite	XPAT protein lacking the protease cleavage linker
XPAT-NoClvSite(1x–C)	XPAT protein lacking the protease cleavage sites; anti-TAA scFV unmasked
XPAT-NoClvSite(1x–N)	XPAT protein lacking the protease cleavage sites; anti-CD3 scFV unmasked

EGFR, endothelial growth factor receptor; HER2, human epidermal growth factor receptor 2; scFv, single-chain variable domain fragment; TAA, tumor-associated antigen; uTCE, unmasked T-cell engager; XPAT protein, TCE fused to XTEN polypeptides.

Supplementary Table 2. Amino acid sequences of the HER2-XPAT, HER2-XPAT-NoClvSite, EGFR-XPAT, and EpCAM-XPAT proteins. The underlined regions are antibody domains and their linkers. The protease-cleavable linker regions are shown in bold text.

HER2-XPAT protein

1	ASHHHHHHSP	AGSPTSTEEG	TSESATPESG	PGTSTEPSEG	SAPGTSESAT	PESGPGSEPA
61	TSGSETPGTS	ESATPESGPG	SEPATSGSET	PGTSESATPE	SGPGTSTEPS	EGSAPGSPAG
121	SPTSTEEGTS	ESATPESGPG	SEPATSGSET	PGTSESATPE	SGPGSPAGSP	TSTEEGSPAG
181	SPTSTEEGTS	TEPSEGSAPG	TSESATPESG	PGTSESATPE	SGPGTSESAT	PESGPGSEPA
241	TSGSETPGSE	PATSGSETPG	SPAGSPTSTE	EGTSTEPSEG	SAPGTSTEPS	EGSAPGGSAP
301	**EAGRSANHTP**	**AGLTGP**ATSG	SETPGTDIQM	TQSPSSLSAS	VGDRVTITCK	ASQDVSIGVA
361	WYQQKPGKAP	KLLIYSASYR	YTGVPSRFSG	SGSGTDFTLT	ISSLQPEDFA	TYYCQQYYIY
421	PYTFGQGTKV	EIKGATPPET	GAETESPGET	TGGSAESEPP	GEGEVQLVES	GGGLVQPGGS
481	LRLSCAASGF	TFTDYTMDWV	RQAPGKGLEW	VADVNPNSGG	SIYNQRFKGR	FTLSVDRSKN
541	TLYLQMNSLR	AEDTAVYYCA	RNLGPSFYFD	YWGQGTLVTV	SSGGGGSELV	VTQEPSLTVS
601	PGGTVTLTCR	SSNGAVTSSN	YANWVQQKPG	QAPRGLIGGT	NKRAPGTPAR	FSGSLLGGKA
661	ALTLSGVQPE	DEAVYYCALW	YPNLWVFGGG	TKLTVLGATP	PETGAETESP	GETTGGSAES
721	EPPGEGEVQL	LESGGGIVQP	GGSLKLSCAA	SGFTFNTYAM	NWVRQAPGKG	LEWVARIRSK
781	YNNYATYYAD	SVKDRFTISR	DDSKNTVYLQ	MNNLKTEDTA	VYYCVRHENF	GNSYVSWFAH
841	WGQGTLVTVS	SGTAEAASAS	G**EAGRSANHT**	**PAGLTGP**PGS	PAGSPTSTEE	GTSESATPES
901	GPGTSTEPSE	GSAPGSPAGS	PTSTEEGTST	EPSEGSAPGT	STEPSEGSAP	GTSESATPES
961	GPGSEPATSG	SETPGSEPAT	SGSETPGSPA	GSPTSTEEGT	SESATPESGP	GTSTEPSEGS
1021	APGTSTEPSE	GSAPGSPAGS	PTSTEEGTST	EPSEGSAPGT	STEPSEGSAP	GTSESATPES
1081	GPGTSTEPSE	GSAPGTSESA	TPESGPGSEP	ATSGSETPGT	STEPSEGSAP	GTSTEPSEGS
1141	APGTSESATP	ESGPGTSESA	TPESGPGSPA	GSPTSTEEGT	SESATPESGP	GSEPATSGSE
1201	TPGTSESATP	ESGPGTSTEP	SEGSAPGTST	EPSEGSAPGT	STEPSEGSAP	GTSTEPSEGS
1261	APGTSTEPSE	GSAPGTSTEP	SEGSAPGSPA	GSPTSTEEGT	STEPSEGSAP	GTSESATPES
1321	GPGSEPATSG	SETPGTSESA	TPESGPGSEP	ATSGSETPGT	SESATPESGP	GTSTEPSEGS
1381	APGTSESATP	ESGPGSPAGS	PTSTEEGSPA	GSPTSTEEGS	PAGSPTSTEE	GTSESATPES
1441	GPGTSTEPSE	GSAPGAAEPE	A			

HER2-XPAT-NoClvSite

1	ASSPAGSPTS	TESGTSESAT	PESGPGTSTE	PSEGSAPGTS	ESATPESGPG	SEPATSGSET
61	PGTSESATPE	SGPGSTPAES	GSETPGTSES	ATPESGPGTS	TEPSEGSAPG	SPAGSPTSTE
121	EGTSESATPE	SGPGSEPATS	GSETPGTSES	ATPESGPGSP	AGSPTSTEEG	SPAGSPTSTE
181	EGTSTEPSEG	SAPGTSESAT	PESGPGTSES	ATPESGPGTS	ESATPESGPG	SEPATSGSET
241	PGSEPATSGS	ETPGSPAGSP	TSTEEGTSTE	PSEGSAPGTS	TEPSEGSAPG	GSAPTTGEAG
301	EAAGATSAGA	TGPATSGSET	PGTDIQMTQS	PSSLSASVGD	RVTITCKASQ	DVSIGVAWYQ
361	QKPGKAPKLL	IYSASYRYTG	VPSRFSGSGS	GTDFTLTISS	LQPEDFATYY	CQQYYIYPYT
421	FGQGTKVEIK	GATPPETGAE	TESPGETTGG	SAESEPPGEG	EVQLVESGGG	LVQPGGSLRL
481	SCAASGFTFT	DYTMDWVRQA	PGKGLEWVAD	VNPNSGGSIY	NQRFKGRFTL	SVDRSKNTLY
541	LQMNSLRAED	TAVYYCARNL	GPSFYFDYWG	QGTLVTVSSG	GGGSELVVTQ	EPSLTVSPGG
601	TVTLTCRSSN	GAVTSSNYAN	WVQQKPGQAP	RGLIGGTNKR	APGTPARFSG	SLLGGKAALT
661	LSGVQPEDEA	VYYCALWYPN	LWVFGGGTKL	TVLGATPPET	GAETESPGET	TGGSAESEPP
721	GEGEVQLLES	GGGIVQPGGS	LKLSCAASGF	TFNTYAMNWV	RQAPGKGLEW	VARIRSKYNN
781	YATYYADSVK	DRFTISRDDS	KNTVYLQMNN	LKTEDTAVYY	CVRHENFGNS	YVSWFAHWGQ
841	GTLVTVSSGT	AEAASASGTT	GEAGEAAGAT	SAGATGPPGS	PAGSPTSTEE	GTSESATPES
901	GPGSEPATSG	SETPGTSESA	TPESGPGTST	EPSEGSAPGT	STEPSEGSAP	GTSTEPSEGS
961	APGTSTEPSE	GSAPGTSTEP	SEGSAPGTST	EPSEGSAPGS	PAGSPTSTEE	GTSTEPSEGS
1021	APGTSESATP	ESGPGSEPAT	SGSETPGTSE	SATPESGPGS	EPATSGSETP	GTSESATPES
1081	GPGTSTEPSE	GSAPGTSESA	TPESGPGSPA	GSPTSTEEGS	PAGSPTSTEE	GSPAGSPTST
1141	EEGTSESATP	ESGPGTSTEP	SEGSAPGTSE	SATPESGPGS	EPATSGSETP	GTSESATPES
1201	GPGSEPATSG	SETPGTSESA	TPESGPGTST	EPSEGSAPGS	PAGSPTSTEE	GTSESATPES

1261	GPGSEPATSG SETPGTSESA TPESGPGSPA GSPTSTEEGS PAGSPTSTEE GTSTEPSEGS
1321	APGTSESATP ESGPGTSESA TPESGPGTSE SATPESGPGS EPATSGSETP GSEPATSGSE
1381	TPGSPAGSPT STEEGTSTEP SEGSAPGTES TPSEGSAPGS EPATSGSETP GTSESATPES
1441	GPGTSTEPSE GSAPGEPEA

EGFR-XPAT protein

1	HHHHHHSPAG SPTSTEEGTS ESATPESGPG TSTEPSEGSA PGTSESATPE SGPGSEPATS
61	GSETPGTSES ATPESGPGSE PATSGSETPG TSESATPESG PGTSTEPSEG SAPGSPAGSP
121	TSTEEGTSES ATPESGPGSE PATSGSETPG TSESATPESG PGSPAGSPTS TEEGSPAGSP
181	TSTEEGTSTE PSEGSAPGTS ESATPESGPG TSESATPESG PGTSESATPE SGPGSEPATS
241	GSETPGSEPA TSGSETPGSP AGSPTSTEEG TSTEPSEGSA PGTSTEPSEG SAPGGSAP**EA**
301	**GRSANHTPAG** **LTGP**ATSGSE TPGTDIQMTQ SPSSLSASVG DRVTITCQAS QDISNYLNWY
361	QQKPGKAPKL LIYDASNLET GVPSRFSGSG SGTDFTFTIS SLQPEDIATY FCQHFDHLPL
421	AFGGGTKVEI KGATPPETGA ETESPGETTG GSAESEPPGE GQVQLQESGP GLVKPSETLS
481	LTCTVSGGSV SSGDYYWTWI RQSPGKGLEW IGHIYYSGNT NYNPSLKSRL TISIDTSKTQ
541	FSLKLSSVTA ADTAIYYCVR DRVTGAFDIW GQGTMVTVSS GGGGSELVVT QEPSLTVSPG
601	GTVTLTCRSS TGAVTTSNYA NWVQQKPGQA PRGLIGGTNK RAPGTPARFS GSLLGGKAAL
661	TLSGVQPEDE AEYYCALWYS NLWVFGGGTK LTVLGATPPE TGAETESPGE TTGGSAESEP
721	PGEGEVQLLE SGGGLVQPGG SLKLSCAASG FTFNTYAMNW VRQAPGKGLE WVARIRSKYN
781	NYATYYADSV KDRFTISRDD SKNTAYLQMN NLKTEDTAVY YCVRHGNFGN SYVSWFAYWG
841	QGTLVTVSSG TAEAASASG**E** **AGRSANHTPA** **GLTGP**PGSPA GSPTSTEEGT SESATPESGP
901	GTSTEPSEGS APGSPAGSPT STEEGTSTEP SEGSAPGTST EPSEGSAPGT SESATPESGP
961	GSEPATSGSE TPGSEPATSG SETPGSPAGS PTSTEEGTSE SATPESGPGT STEPSEGSAP
1021	GTSTEPSEGS APGSPAGSPT STEEGTSTEP SEGSAPGTST EPSEGSAPGT SESATPESGP
1081	GTSTEPSEGS APGTSESATP ESGPGSEPAT SGSETPGTST EPSEGSAPGT STEPSEGSAP
1141	GTSESATPES GPGTSESATP ESGPGSPAGS PTSTEEGTSE SATPESGPGS EPATSGSETP
1201	GTSESATPES GPGTSTEPSE GSAPGTSTEP SEGSAPGTST EPSEGSAPGT STEPSEGSAP
1261	GTSTEPSEGS APGTSTEPSE GSAPGSPAGS PTSTEEGTST EPSEGSAPGT SESATPESGP
1321	GSEPATSGSE TPGTSESATP ESGPGSEPAT SGSETPGTSE SATPESGPGT STEPSEGSAP
1381	GTSESATPES GPGSPAGSPT STEEGSPAGS PTSTEEGSPA GSPTSTEEGT SESATPESGP
1441	GTSTEPSEGS APGTSESATP ESGPGSEPAT SGSETPGTSE SATPESGPGS EPATSGSETP
1501	GTSESATPES GPGTSTEPSE GSAPGSPAGS PTSTEEGTSE SATPESGPGS EPATSGSETP
1561	GTSESATPES GPGSPAGSPT STEEGSPAGS PTSTEEGTST EPSEGSAPGT SESATPESGP
1621	GTSESATPES GPGTSESATP ESGPGSEPAT SGSETPGSEP ATSGSETPGS PAGSPTSTEE
1681	GTSTEPSEGS APGTSTEPSE GSAPGSEPAT SGSETPGTSE SATPESGPGT STEPSEGAAE
1741	PEA

EpCAM-XPAT-Cys protein

1	ASHHHHHHSP AGSPTSTEEG TSESATPESG PGTSTEPSEG SAPGTSESAT PESGPGSEPA
61	TSGSETPGTS ESATPESGPG SEPATSGSET PGTSESATPE SGPGTSTEPS EGSAPGSPAG
121	SPTSTEEGTS ESATPESGPG SEPATSGSET PGTSESATPE SGPGSPAGSP TSTEEGSPAG
181	SPTSTEEGTS TEPSEGSAPG TSESATPESG PGTSESATPE SGPGTSESAT PESGPGSEPA
241	TSGSETPGSE PATSGSETPG SPAGSPTSTE EGTSTEPSEG SAPGTSTEPS EGSAPGGSAP
301	**EAGRSANHTP** **AGLTGP**ATSG SETPGTDIQM TQSPSSLSAS VGDRVTITCR STKSLLHSNG
361	ITYLYWYQQK PGKAPKLLIY QMSNLASGVP SRFSSSGSGT DFTLTISSLQ PEDFATYYCA
421	QNLEIPRTFG QGTKVEIKGA TPPETGAETE SPGETTGGSA ESEPPGEGQV QLVQSGPGLV
481	QPGGSVRISC AASGYTFTNY GMNWVKQAPG KGLEWMGWIN TYTGESTYAD SFKGRFTFSL
541	DTSASAAYLQ INSLRAEDTA VYYCARFAIK GDYWGQGTLL TVSSGGGGSE LVVTQEPSLT
601	VSPGGTVTLT CRSSNGAVTS SNYANWVQQK PGQAPRGLIG GTNKRAPGTP ARFSGSLLGG
661	KAALTLSGVQ PEDEAVYYCA LWYPNLWVFG GGTKLTVLGA TPPETGAETE SPGETTGGSA
721	ESEPPGEGEV QLLESGGGIV QPGGSLKLSC AASGFTFNTY AMNWVRQAPG KGLEWVARIR
781	SKYNNYATYY ADSVKDRFTI SRDDSKNTVY LQMNNLKTED TAVYYCVRHE NFGNSYVSWF
841	AHWGQGTLVT VSSGTAEAAS ACG**EAGRSAN** **HTPAGLTGP**P GSPAGSPTST EEGTSESATP
901	ESGPGTSTEP SEGSAPGSPA GSPTSTEEGT STEPSEGSAP GTSTEPSEGS APGTSESATP
961	ESGPGSEPAT SGSETPGSEP ATSGSETPGS PAGSPTSTEE GTSESATPES GPGTSTEPSE

1021	GSAPGTSTEP SEGSAPGSPA GSPTSTEEGT STEPSEGSAP GTSTEPSEGS APGTSESATP
1081	ESGPGTSTEP SEGSAPGTSE SATPESGPGS EPATSGSETP GTSTEPSEGS APGTSTEPSE
1141	GSAPGTSESA TPESGPGTSE SATPESGPGS PAGSPTSTEE GTSESATPES GPGSEPATSG
1201	SETPGTSESA TPESGPGTST EPSEGSAPGT STEPSEGSAP GTSTEPSEGS APGTSTEPSE
1261	GSAPGTSTEP SEGSAPGTST EPSEGSAPGS PAGSPTSTEE GTSTEPSEGS APGTSESATP
1321	ESGPGSEPAT SGSETPGTSE SATPESGPGS EPATSGSETP GTSESATPES GPGTSTEPSE
1381	GSAPGTSESA TPESGPGSPA GSPTSTEEGS PAGSPTSTEE GSPAGSPTST EEGTSESATP
1441	ESGPGTSTEP SEGSAPGAAE PEA
**HER2-XPAT-Cys protein**	
1	ASHHHHHHSP AGSPTSTEEG TSESATPESG PGTSTEPSEG SAPGTSESAT PESGPGSEPA
61	TSGSETPGTS ESATPESGPG SEPATSGSET PGTSESATPE SGPGTSTEPS EGSAPGSPAG
121	SPTSTEEGTS ESATPESGPG SEPATSGSET PGTSESATPE SGPGSPAGSP TSTEEGSPAG
181	SPTSTEEGTS TEPSEGSAPG TSESATPESG PGTSESATPE SGPGTSESAT PESGPGSEPA
241	TSGSETPGSE PATSGSETPG SPAGSPTSTE EGTSTEPSEG SAPGTSTEPS EGSAPGGSAP
301	**EAGRSANHTP AGLTGP**ATSG SETPGTDIQM TQSPSSLSAS VGDRVTITCK ASQDVSIGVA
361	WYQQKPGKAP KLLIYSASYR YTGVPSRFSG SGSGTDFTLT ISSLQPEDFA TYYCQQYYIY
421	PYTFGQGTKV EIKGATPPET GAETESPGET TGGSAESEPP GEGEVQLVES GGGLVQPGGS
481	LRLSCAASGF TFTDYTMDWV RQAPGKGLEW VADVNPNSGG SIYNQRFKGR FTLSVDRSKN
541	TLYLQMNSLR AEDTAVYYCA RNLGPSFYFD YWGQGTLVTV SSGGGGSELV VTQEPSLTVS
601	PGGTVTLTCR SSNGAVTSSN YANWVQQKPG QAPRGLIGGT NKRAPGTPAR FSGSLLGGKA
661	ALTLSGVQPE DEAVYYCALW YPNLWVFGGG TKLTVLGATP PETGAETESP GETTGGSAES
721	EPPGEGEVQL LESGGGIVQP GGSLKLSCAA SGFTFNTYAM NWVRQAPGKG LEWVARIRSK
781	YNNYATYYAD SVKDRFTISR DDSKNTVYLQ MNNLKTEDTA VYYCVRHENF GNSYVSWFAH
841	WGQGTLVTVS SGTAEAASAC G**EAGRSANHT PAGLTGP**PGS PAGSPTSTEE GTSESATPES
901	GPGTSTEPSE GSAPGSPAGS PTSTEEGTST EPSEGSAPGT STEPSEGSAP GTSESATPES
961	GPGSEPATSG SETPGSEPAT SGSETPGSPA GSPTSTEEGT SESATPESGP GTSTEPSEGS
1021	APGTSTEPSE GSAPGSPAGS PTSTEEGTST EPSEGSAPGT STEPSEGSAP GTSESATPES
1081	GPGTSTEPSE GSAPGTSESA TPESGPGSEP ATSGSETPGT STEPSEGSAP GTSTEPSEGS
1141	APGTSESATP ESGPGTSESA TPESGPGSPA GSPTSTEEGT SESATPESGP GSEPATSGSE
1201	TPGTSESATP ESGPGTSTEP SEGSAPGTST EPSEGSAPGT STEPSEGSAP GTSTEPSEGS
1261	APGTSTEPSE GSAPGTSTEP SEGSAPGSPA GSPTSTEEGT STEPSEGSAP GTSESATPES
1321	GPGSEPATSG SETPGTSESA TPESGPGSEP ATSGSETPGT SESATPESGP GTSTEPSEGS
1381	APGTSESATP ESGPGSPAGS PTSTEEGSPA GSPTSTEEGS PAGSPTSTEE GTSESATPES
1441	GPGTSTEPSE GSAPGAAEPE A

192

Supplementary Table 3. Tumor types used for patient-derived cancer xenograft models in immunodeficient mice.

Tumor number	Tumor type	XPAT protein tumor target
CTG-2543	NSCLC	HER2
CTG-1569	RCC	HER2
CTG-0828	NSCLC	HER2
CTG-2432	Breast	HER2
CTG-3303	Breast	HER2
CTG-0835	Colorectal	EpCAM
CTG-2647	Endometrial	EpCAM
CTG-0936	Gastric	EpCAM
CTG-0138	Esophageal	EpCAM
CTG-1680	NSCLC	EpCAM

Supplementary Table 4. Flow cytometry antigens and cell populations analyzed (NHP studies).

Antigen markers	Cell population identified[a]	Parameters reported
CD45+/CD3+/CD16-	T-lymphocytes	% Parent
CD45+/CD16-/CD8+/CD4-	T-cytotoxic lymphocytes	% Grandparent
CD45+/ CD16-/CD8+/CD4-/CD69+	CD69+ T-cytotoxic lymphocytes[b]	% Parent and CD69-BV421 MFI
CD45+/ CD16-/CD8+/CD4-/CD25+	CD25+ T-cytotoxic lymphocytes[c]	% Parent and CD25-APC MFI
CD45+/ CD16-/CD8-/CD4+	T-helper lymphocytes	% Grandparent
CD45+/ CD16-/CD8-/CD4+/CD69+	CD69+ T-helper lymphocytes[b]	% Parent and CD69-BV421 MFI
CD45+/ CD16-/CD8-/CD4+/CD25+	CD25+ T-helper lymphocytes[c]	% Parent and CD25-APC MFI
CD45+/CD3-/CD16+	Natural killer cells	% Parent

[a] Relative percentages and percent of baseline of relative percentages were calculated and reported for all parameters.

[b] MFI of CD69-BV421 gated positive was reported.

[c] MFI of CD25-APC gated positive was reported.

MFI, mean fluorescence intensity; NHP, non-human primates.

Supplementary Table 5. List of human samples used for the analysis of HER2-XPAT protein plasma stability.

Patient Characteristics	Catalog number (BioIVT)	Lot number
Healthy	HUMANPLNHU2N	HMN168642
Healthy	HUMANPLNHU2N	HMN168638
Healthy	HUMANPLNHU2N	HMN168639
Healthy	HUMANPLNHU2N	HMN168640
Lung Cancer	HMPLNH-LGCA	HMN88660
Lung Cancer	HMPLNH-LGCA	HMN88661
Lung Cancer	HMPLNH-LGCA	HMN88662
Lung Cancer	HMPLNH-LGCA	HMN88663
Lung Cancer	HMPLNH-LGCA	HMN88664
Lung Cancer	HMPLNH-LGCA	HMN88665
Lung Cancer	HMPLNH-LGCA	HMN88667
Breast Cancer	HMPLNH-BRCA	HMN88668
Breast Cancer	HMPLNH-BRCA	HMN88669
Breast Cancer	HMPLNH-BRCA	HMN88673
Colon Cancer	HMPLNH-COCA	HMN88671
Rheumatoid Arthritis	HMPLNH-RA	HMN101674
Rheumatoid Arthritis	HMPLNH-RA	HMN101675
Rheumatoid Arthritis	HMPLNH-RA	HMN101676
Rheumatoid Arthritis	HMPLNH-RA	HMN101677
Rheumatoid Arthritis	HMPLNH-RA	HMN101680
Rheumatoid Arthritis	HMPLNH-RA	HMN101681
Rheumatoid Arthritis	HMPLNH-RA	HMN101682
Rheumatoid Arthritis	HMPLNH-RA	HMN101683
Multiple Sclerosis	HMPLNH-MS	HMN101684
Multiple Sclerosis	HMPLNH-MS	HMN101685

Multiple Sclerosis	HMPLNH-MS	HMN101686
Multiple Sclerosis	HMPLNH-MS	HMN101687
Multiple Sclerosis	HMPLNH-MS	HMN101688
Multiple Sclerosis	HMPLNH-MS	HMN101689
Systemic Lupus Erythematosus	HMPLNH-SLE	HMN101691
Systemic Lupus Erythematosus	HMPLNH-SLE	HMN101692
Systemic Lupus Erythematosus	HMPLNH-SLE	HMN101693
Systemic Lupus Erythematosus	HMPLNH-SLE	HMN101694
Systemic Lupus Erythematosus	HMPLNH-SLE	HMN101695
Systemic Lupus Erythematosus	HMPLNH-SLE	HMN101697
Systemic Lupus Erythematosus	HMPLNH-SLE	HMN101698
Systemic Lupus Erythematosus	HMPLNH-SLE	HMN101699
Inflammatory Bowel Disease	HMPLNH-IBD	HMN101700
Inflammatory Bowel Disease	HMPLNH-IBD	HMN101701
Inflammatory Bowel Disease	HMPLNH-IBD	HMN101702
Inflammatory Bowel Disease	HMPLNH-IBD	HMN101703
Inflammatory Bowel Disease	HMPLNH-IBD	HMN101704
