# Supplementary material for: Precision-activated T-cell engagers targeting HER2 or EGFR and CD3 mitigate on-target, off-tumor toxicity for immunotherapy in solid tumors
Source: Nat Cancer. 2023 Mar 30;4(4):485–501. doi: 10.1038/s43018-023-00536-9 (PMC10132983; doi:10.1038/s43018-023-00536-9)
Supplement: Supplementary file 2 — Reporting Summary [file 43018_2023_536_MOESM2_ESM.pdf]

nature portfolio

Corresponding author(s): Volker Schellenberger

Last updated by author(s): Jan 17, 2023

Reporting Summary

Nature Portfolio wishes to improve the reproducibility of the work that we publish. This form provides structure for consistency and transparency in reporting. For further information on Nature Portfolio policies, see our Editorial Policies and the Editorial Policy Checklist.

Statistics

For all statistical analyses, confirm that the following items are present in the figure legend, table legend, main text, or Methods section.

n/a	Confirmed	
☐	☒	The exact sample size (*n*) for each experimental group/condition, given as a discrete number and unit of measurement
☐	☒	A statement on whether measurements were taken from distinct samples or whether the same sample was measured repeatedly
☐	☒	The statistical test(s) used AND whether they are one- or two-sided<br>*Only common tests should be described solely by name; describe more complex techniques in the Methods section.*
☒	☐	A description of all covariates tested
☒	☐	A description of any assumptions or corrections, such as tests of normality and adjustment for multiple comparisons
☐	☒	A full description of the statistical parameters including central tendency (e.g. means) or other basic estimates (e.g. regression coefficient) AND variation (e.g. standard deviation) or associated estimates of uncertainty (e.g. confidence intervals)
☐	☒	For null hypothesis testing, the test statistic (e.g. *F*, *t*, *r*) with confidence intervals, effect sizes, degrees of freedom and *P* value noted<br>*Give P values as exact values whenever suitable.*
☒	☐	For Bayesian analysis, information on the choice of priors and Markov chain Monte Carlo settings
☒	☐	For hierarchical and complex designs, identification of the appropriate level for tests and full reporting of outcomes
☒	☐	Estimates of effect sizes (e.g. Cohen's *d*, Pearson's *r*), indicating how they were calculated

Our web collection on statistics for biologists contains articles on many of the points above.

Software and code

Policy information about availability of computer code

Data collection	SoftMax Pro v7.1, MSD DISCOVERY WORKBENCH (Mesoscale Discovery) - Model 1300, Luminex xPONENT v4.3.229.0 on MAGPIX System, LI-COR Odyssey CLx, LI-COR Image Studio Lite v.2, Biacore 4000, BD FACSCanto™ II flow cytometer, BD FACSDiva® software v9.0; AlphaFold v2.0; GraphPad Prism V 9.5.0 (730; San Diego, CA)
Data analysis	Statistical analyses were performed using GraphPad Prism software V 9.5.0 (730; San Diego, CA); flow cytometry, binding kinetics and imaging data were analyzed using software associated with the equipment used for data capture (described above). No custom algorithms or software were used.

For manuscripts utilizing custom algorithms or software that are central to the research but not yet described in published literature, software must be made available to editors and reviewers. We strongly encourage code deposition in a community repository (e.g. GitHub). See the Nature Portfolio guidelines for submitting code & software for further information.

Data

Policy information about availability of data

All manuscripts must include a data availability statement. This statement should provide the following information, where applicable:

- Accession codes, unique identifiers, or web links for publicly available datasets
- A description of any restrictions on data availability
- For clinical datasets or third party data, please ensure that the statement adheres to our policy

Source data for Fig. 2–7 and Extended Data Fig. 1–3 have been provided in the Source Data file. All other data supporting the findings of this study are available from the corresponding author on reasonable request.

Human research participants

Policy information about studies involving human research participants and Sex and Gender in Research.

Reporting on sex and gender	Please refer to BioIVT, Champions Oncology, Oncodesign Precision Medicine, Crown Bioscience, and Etablissement Francais du Sang. Information about sex and gender was not collected as no gender/sex analyses were conducted (not considered relevant to this study).
Population characteristics	Please refer to BioIVT, Champions Oncology, Oncodesign Precision Medicine, Crown Bioscience, and Etablissement Francais du Sang. Clinical information about the healthy volunteers and patients with cancer and inflammatory disease (whose plasma samples were used to assess plasma stability of HER2-XPAT protein) is summarized in Supplementary Table 5.
Recruitment	Please refer to BioIVT, Champions Oncology, Oncodesign Precision Medicine, Crown Bioscience, and Etablissement Francais du Sang.
Ethics oversight	Human PBMCs and healthy volunteer/patient serum were purchased by BioIVT, Champions Oncology, Oncodesign Precision Medicine, Crown Bioscience, and Etablissement Francais du Sang, who collected informed consent from the patients.

Note that full information on the approval of the study protocol must also be provided in the manuscript.

Field-specific reporting

Please select the one below that is the best fit for your research. If you are not sure, read the appropriate sections before making your selection.

☒ Life sciences ☐ Behavioural & social sciences ☐ Ecological, evolutionary & environmental sciences

For a reference copy of the document with all sections, see nature.com/documents/nr-reporting-summary-flat.pdf

Life sciences study design

All studies must disclose on these points even when the disclosure is negative.

Sample size	Minimum numbers were determined based on pertinent literature for comparable studies in these models in which desired effect sizes were shown to be statistically significant.<br>The following information was used to determine the sample size for the mouse tumor xenograft models. Maximum human donor PBMC availability needed for the study plus the anticipated variability in response and heterogeneity of tumor growth for the particular tumor model were key determining factors in choosing the amount of animals per group. The following publication gave some insight into proper statistical powering:<br>How to calculate sample size in animal studies? J Pharmacol Pharmacotherapeutics. 2013 Oct-Dec; 4(4): 303–306.<br>Their proposed formula of E = (Total number of animals) – (Total number of groups) stipulates that an E>10 is adequately powered. In the case of the three in-vivo studies performed in this manuscript, the E is calculated as between 25-28, signifying that the studies were adequately powered. The highly significant p-values for the tumor growth inhibition also signify that the group sizes were adequately powered.
Data exclusions	No animals or data points were excluded from data analyses for any of the experiments
Replication	The number of replicates per experiment were sufficient to establish the treatment effects (and statistical significance, where appropriate). Experiments were not further replicated.
Randomization	For tumor xenograft studies, randomization was performed using a tumor volume-stratified randomization method. Each test within an in-vitro experiment was conducted under identical test conditions, with the only variable being the test material. Therefore, randomization was not implemented and there was no need to adjust for any covariates in the data analysis.
Blinding	Blinding was not possible due to the Contract Research Organization needing to identify test articles for reconstitution with the vehicle diluent control, with the same party responsible for treatment allocation and data collection/analysis.

Reporting for specific materials, systems and methods

We require information from authors about some types of materials, experimental systems and methods used in many studies. Here, indicate whether each material, system or method listed is relevant to your study. If you are not sure if a list item applies to your research, read the appropriate section before selecting a response.

Materials & experimental systems

n/a	Involved in the study	
☐	☒	Antibodies
☐	☒	Eukaryotic cell lines
☒	☐	Palaeontology and archaeology
☐	☒	Animals and other organisms
☒	☐	Clinical data
☒	☐	Dual use research of concern

Methods

n/a	Involved in the study	
☒	☐	ChIP-seq
☐	☒	Flow cytometry
☒	☐	MRI-based neuroimaging

Antibodies

Antibodies used

Viobility 405/452 Fixable Dye eq BV421, Miltenyi Biotec, 130-110-205, 1 µL for 107 cells/100 µL
CD45 Viogreen, Miltenyi Biotec, 130-110-803, REA737, mouse, 1:50
CD45 FITC, BD Biosciences, 555482, HI30, human, 1:5
CD4 PE, BD Biosciences, 555347, RPA-T4, human, 1:5
CD8 PE Vio615, Miltenyi Biotec, 130-110-685, REA734, human, 1:50
HLA DR PerCP-Vio700, Miltenyi Biotec, 130-111-793, REA805, human, 1:50
CD25 PE-Vio770, Miltenyi Biotec, 130-116-205, REA945, human, 1:50
CD3 APC, BD Biosciences, 555335, UCHT1, human, 1:5
CD69 APC-vio770, Miltenyi Biotec, 130-112-616, REA824, human, 1:50

Antibodies Clone Fluorophore Channels
CD3 SP34.2 FITC FL1
CD4 L200 APC FL8
CD8 BW135-80 PECy7 FL5
CD16 3G8 PECy7 FL5
CD69 FN50 PE FL2
CD25 M-A251 PerCPCy5.5 FL4

Validation

All antibodies used were validated by the supplier for their application. Validation statements and relevant references for the antibodies used are available on the manufacturers' websites.

Eukaryotic cell lines

Policy information about cell lines and Sex and Gender in Research

Cell line source(s)

SKOV3 (ATCC HTB-77), BT-474 (ATCC HTB-20), MCF-7 (ATCC HTB-22) and HT-29 (ATCC HTB-38) cell lines were purchased from ATCC. HT-55 cell lines were obtained from Cobioer, Nanjing, China, (CBP60012). JURKAT reporter T cells were purchased from Promega (J1625, J1601).

Authentication

IDEXX, STR

Mycoplasma contamination

Cell lines were tested negative for mycoplasma

Commonly misidentified lines
(See ICLAC register)

None of the cell lines used are on the list of known misidentified cell lines.

Animals and other research organisms

Policy information about studies involving animals; ARRIVE guidelines recommended for reporting animal research, and Sex and Gender in Research

Laboratory animals

Female mIce aged 6 to 8 weeks: non-obese diabetic/Shiscid IL2rgammanull (NOG), non-obese diabetic (NOD)-PrkdcscidIl2rgnull (NPSG), and NOD.Cg-Prkdc<scid> Il2rg<tm1Wjl>/SzJ mice
For tumor xenograft experiments, the maximal tumor size/burden was prespecified as 2000 mm3, and individual animals measured with a tumor burden exceeding this volume were humanely euthanized.
Cynomolgus monkeys (Chinese, Cambodian), including both male and female animals, were used in the toxicology studies. The age of the animals at the initiation of dosing was between 35 to 43 months.

Wild animals	The study did not involve wild animals
Reporting on sex	Female mice
Field-collected samples	The study did not involve samples collected from the field
Ethics oversight	All studies involving animals were conducted at specialist research centers, which were accredited by the Association for Assessment and Accreditation of Laboratory Animal Care. The study protocols were approved by The Institutional Animal Care and Use Committees of BioDuro Sundia (IACUC protocol FFS-001), Crown Bioscience (IACUC protocol AN-1903-05-1759) and Explora BioLabs (IACUC protocol EB17-010).

Note that full information on the approval of the study protocol must also be provided in the manuscript.

Flow Cytometry

Plots

Confirm that:

☒ The axis labels state the marker and fluorochrome used (e.g. CD4-FITC).

☒ The axis scales are clearly visible. Include numbers along axes only for bottom left plot of group (a 'group' is an analysis of identical markers).

☒ All plots are contour plots with outliers or pseudocolor plots.

☒ A numerical value for number of cells or percentage (with statistics) is provided.

Methodology

Sample preparation	T-cell activation was assessed in mice bearing tumor xenografts. Mouse blood was collected in tubes with anticoagulant (K2 EDTA) by intra-cardiac puncture, as a terminal procedure under deep isoflurane gas anesthesia. Red blood cells were lysed with Versalyse lysing buffer. Excised mouse tumors were dissected into smaller fragments using scalpels and further dissociated into single cell suspensions in a non-enzymatic cell dissociation buffer, incubated at 37°C for 30 minutes, and mechanically separated through a 70 µm cell strainer. Viable cells were enriched using ficoll-based gradient centrifugation. T-cell surface activation markers, CD25 and CD69 on CD4+ and CD8+ T-cell subsets, were measured by flow cytometry.<br><br>Changes in immune profiles in peripheral blood were analyzed by flow cytometry in the single-dose toxicokinetic studies evaluating XPAT proteins and their equivalent unmasked TCEs in NHPs. Blood was collected at the time points described, and centrifuged to separate the cells and then treated with Lysis buffer to remove RBCS. The samples were then stained with the cocktail of detection antibodies in staining buffer, washed and read on the Flow Cytometer. The flow cytometry experiments were performed by a Contract Research Organization (CRL, Envol Biomedical).
Instrument	Cyan ADP Flow Cytometer; LSR Fortessa X20; BD FACSCanto™ II flow cytometer
Software	Summit Software; FlowJo; BD FACSDiva® software v9.0
Cell population abundance	Cell sorting was not performed
Gating strategy	CD3/CD45/CD4/CD8 gated cell populations were analyzed<br>In the FSC-A v SSC-A plot, debris was excluded to determine cells of interest. These cells were gated for singlet (FSC-H v FSC-A and SSC-H v SSC-A) and viable cells (Viobility 405/452 Fixable Dye negative v SSC-A/FSC-A. The positive signal is determined against unstained cells). All positive immune cell and activation markers were determined by a combination of isotype and FMO controls.

☒ Tick this box to confirm that a figure exemplifying the gating strategy is provided in the Supplementary Information.
